# RNA-seq, de novo transcriptome assembly and flavonoid gene analysis in 13 wild and cultivated berry fruit species with high content of phenolics

**DOI:** 10.1186/s12864-019-6183-2

**Published:** 2019-12-19

**Authors:** Vera Thole, Jean-Etienne Bassard, Ricardo Ramírez-González, Martin Trick, Bijan Ghasemi Afshar, Dario Breitel, Lionel Hill, Alexandre Foito, Louise Shepherd, Sabine Freitag, Cláudia Nunes dos Santos, Regina Menezes, Pilar Bañados, Michael Naesby, Liangsheng Wang, Artem Sorokin, Olga Tikhonova, Tatiana Shelenga, Derek Stewart, Philippe Vain, Cathie Martin

**Affiliations:** 1 Department of Metabolic Biology, John Innes Centre, Norwich Research Park, Norwich, NR4 7UH UK; 2 Department of Plant and Environmental Science, University of Copenhagen, 1871 Frederiksberg, Denmark; 3 Present address: Institute of Plant Molecular Biology, CNRS, University of Strasbourg, 12 Rue General Zimmer, 67084 Strasbourg, France; 4 Department of Crop Genetics, John Innes Centre, Norwich Research Park, Norwich, NR4 7UH UK; 5 Department of Computational and Systems Biology, John Innes Centre, Norwich Research Park, Norwich, NR4 7UH UK; 6 Present address: Tropic Biosciences UK LTD, Norwich Research Park, Norwich, NR4 7UG UK; 7 The James Hutton Institute, Invergowrie, Dundee, DD2 5DA UK; 8 Instituto de Biologia Experimental e Tecnológica, Av. República, Qta. do Marquês, 2780-157 Oeiras, Portugal; 9 CEDOC, NOVA Medical School, Faculdade de Ciências Médicas, Universidade NOVA de Lisboa, Rua Câmara Pestana 6, 1150-082 Lisbon, Portugal; 10 Instituto de Tecnologia Química e Biológica António Xavier, Universidade Nova de Lisboa, Av. da República, 2780-157 Oeiras, Portugal; 11 Facultad De Agronomía e Ingeniería Forestal, Pontificia Universidad Católica de Chile, Av. Vicuña Mackenna Ote, 4860 Macul, Chile; 12 Evolva, Duggingerstrasse 23, 4153 Reinach, Switzerland; 13 Institute of Botany, The Chinese Academy of Sciences, 20 Nanxincun, Xiangshan, Beijing, 100093 China; 14 Fruit Crops Genetic Resources Department, N. I. Vavilov Research Institute of Plant Industry, B. Morskaya Street 42-44, St. Petersburg, 190000 Russia; 15 Institute of Mechanical, Process and Energy Engineering, School of Engineering and Physical Sciences, Heriot Watt University, Edinburgh, UK

**Keywords:** 13 berry fruit species, RNA-seq, de novo assembly, Anthocyanin, Gene expression analysis, Fruit ripening, Transcription factors, MYB, bHLH, WDR

## Abstract

**Background:**

Flavonoids are produced in all flowering plants in a wide range of tissues including in berry fruits. These compounds are of considerable interest for their biological activities, health benefits and potential pharmacological applications. However, transcriptomic and genomic resources for wild and cultivated berry fruit species are often limited, despite their value in underpinning the in-depth study of metabolic pathways, fruit ripening as well as in the identification of genotypes rich in bioactive compounds.

**Results:**

To access the genetic diversity of wild and cultivated berry fruit species that accumulate high levels of phenolic compounds in their fleshy berry(-like) fruits, we selected 13 species from Europe, South America and Asia representing eight genera, seven families and seven orders within three clades of the kingdom *Plantae*. RNA from either ripe fruits (ten species) or three ripening stages (two species) as well as leaf RNA (one species) were used to construct, assemble and analyse de novo transcriptomes. The transcriptome sequences are deposited in the BacHBerryGEN database (http://jicbio.nbi.ac.uk/berries) and were used, as a proof of concept, *via* its BLAST portal (http://jicbio.nbi.ac.uk/berries/blast.html) to identify candidate genes involved in the biosynthesis of phenylpropanoid compounds. Genes encoding regulatory proteins of the anthocyanin biosynthetic pathway (MYB and basic helix-loop-helix (bHLH) transcription factors and WD40 repeat proteins) were isolated using the transcriptomic resources of wild blackberry (*Rubus genevieri*) and cultivated red raspberry (*Rubus idaeus* cv. Prestige) and were shown to activate anthocyanin synthesis in *Nicotiana benthamiana*. Expression patterns of candidate flavonoid gene transcripts were also studied across three fruit developmental stages *via* the BacHBerryEXP gene expression browser (http://www.bachberryexp.com) in *R. genevieri* and *R. idaeus* cv. Prestige.

**Conclusions:**

We report a transcriptome resource that includes data for a wide range of berry(-like) fruit species that has been developed for gene identification and functional analysis to assist in berry fruit improvement. These resources will enable investigations of metabolic processes in berries beyond the phenylpropanoid biosynthetic pathway analysed in this study. The RNA-seq data will be useful for studies of berry fruit development and to select wild plant species useful for plant breeding purposes.

## Background

Berry fruit species span numerous plant families placing considerable demands on the genomics resources required to study fruit development, gene expression and biosynthesis of bioactive compounds. Over the past few years, genome sequences of woodland strawberry (*Fragaria vesca*) [1], highbush blueberry (*Vaccinium corymbosum*) [2, 3], cranberry (*Vaccinium macrocarpon*) [4], grapevine varieties (*Vitis vinifera*) [5, 6], black raspberry (*Rubus occidentalis*) [7] and more recently, wild blackberry (*Rubus ulmifolius*) [8] have been released. Fruit transcriptomes of red raspberry (*Rubus idaeus* cv. Nova) [9], Korean black raspberry (*Rubus coreanus*) [10], blue honeysuckle (*Lonicera caerulea*) [11], highbush blueberry varieties [12–15], cranberry [16], grapevine varieties [17–22], cultivated blackberry (*Rubus* sp. var. Lochness) [23], woodland [24] and cultivated strawberry (*F. × ananassa*) [25] are also available. A wealth of transcriptome information for organs and tissues of berry fruit species has also been reported. Here, we aimed to bridge some of the gaps currently existing in berry fruit RNA-seq resources by generating and analyzing the fruit transcriptomes of 12 species as well as the leaf transcriptome of an additional species as part of the the BacHBerry (BACterial Hosts for production of Bioactive phenolics from bERRY fruits) collaborative project [26].

Plant-based products like fruits and berries are essential parts of the human diet and are considered healthy and nutritious foods (reviewed in [27]). Many berries and fruits are valued for their high content of bioactive compounds, including specialised metabolites of the phenylpropanoid pathway such as flavonoids (flavonols, flavones, isoflavones, anthocyanins and proanthocyanidins). Berries and fruits also contain other beneficial compounds such as carotenoids, vitamins, minerals and terpenoids. Beneficial health effects have been studied in several species that were sequenced here including wild blackberries (*Rubus vagabundus*), blueberries (*V. corymbosum*), honeysuckle (*L. caerulea*), Maqui berry (*Aristotelia chilensis*), strawberry myrtle (*Ugni molinae*), raspberries (*R. idaeus*) [28–34] and crowberry (*Corema album*) [35, 36]. Health benefits have often been attributed to phenolic compounds, which have been shown to possess anti-inflammatory, anti-mutagenic, anti-microbial, anti-carcinogenic, anti-obesity, anti-allergic, antioxidant as well as neuro- and cardioprotective properties (for a review see [37] and references therein). Polyphenols also exhibit valuable functions in plants such as protecting against UV radiation and high light stress, acting as signaling molecules and helping to attract pollinators by means of floral pigments.

The plant species chosen in this study had been shown to contain a diverse profile of phenolic compounds, especially anthocyanins: *A. chilensis* [38–41], *Berberis buxifolia* (Calafate) [42], *C. album* [43], *L. caerulea* [44, 45], *Rubus genevieri* (blackberry) [26], *R. idaeus* [46], *Ribes nigrum* (blackcurrant) [47], *R. vagabundus* [33], *U. molinae* [48], *V. corymbosum* [48] and *Vaccinium uliginosum* (Bog bilberry) [49]. Some of these berries, such as Calafate, Maqui berry and strawberry myrtle, are often referred to as ‘superfruits’ because of their exceptionally high antioxidant capacities. These species were investigated for new bioactive compounds and new bioactivities together with the identification of their polyphenolic compounds such as anthocyanins [26, 50, 51].

The synthesis of phenylpropanoids, specifically anthocyanins and other flavonoids, has been studied in many plant species such as *Arabidopsis thaliana* (thale cress), *Antirrhinum majus* (snapdragon), *Malus x domestica* (apple), *Petunia x hybrida* (petunia), *Solanum lycopersicum* (tomato), *V. vinifera* and *Zea mays* (maize) (reviewed in [52]), although, phenylpropanoid biosynthesis has been less well investigated in berry fruit species. Anthocyanins are water-soluble plant pigments responsible for the red, purple or blue colouring of many plant tissues, especially flowers and fruits. Genes required for the formation of flavonoids are predominantly controlled at the transcriptional level. Members of several protein superfamilies mediate the transcriptional regulation of the flavonoid biosynthetic pathway, namely the MYB transcription factors (TFs), basic helix-loop-helix (bHLH) TFs and conserved WD40 repeat (WDR) proteins [53].

The MYB TFs that regulate flavonol, anthocyanin and proanthocyanidin (PA) biosynthesis harbor a highly conserved N-terminal MYB domain consisting of two imperfect tandem repeats (R2 and R3, R2R3-MYB) that function in DNA binding and protein-protein interactions (reviewed in [54]). Some MYB TFs can interact with bHLH transcriptional regulators and WDR proteins to form a dynamic transcriptional activation complex (MBW complex) that regulates the transcription of genes involved in anthocyanin and PA biosynthesis [55]. R2R3-type MYB TFs such as AtMYB12 from *A. thaliana* act independently of a bHLH cofactor and control the expression of genes encoding enzymes operating early in the flavonol biosynthetic pathway. MYB TFs are often specific for the genes and pathway/pathway branches they target, such as the flavonol-specific activators of the R2R3 MYB subgroup (SG) 7 (*e.g.*, AtMYB12 [56]) whereas others are confined to regulating anthocyanin (MYB SG6, *A. majus* AmROSEA1 [57]) or PA biosynthesis (MYB SG5, *A. thaliana* AtTT2 [58]). Many R2R3-type MYB TFs, for instance MdMYB10 from *M. domestica* [59], activate flavonoid synthesis whereas some others can repress anthocyanin formation (*P. hybrida* MYB27 [60]). In contrast, bHLH proteins may have multiple regulatory targets [61] and can control transcription of several branches of the flavonoid pathway as shown, for instance, by AtTT8 from *A. thaliana* [58] and Noemi from *Citrus medica* [62] in the regulation of both anthocyanin and PA biosynthesis.

Among the large class of bHLH TFs, bHLH transcriptional regulators related to flavonoid synthesis (SG IIIf [63],) consist of a MYB-interacting region (MIR) at their N-terminus, a neighboring WD40/acidic domain (AD) necessary for interaction with WDR proteins and/or RNA polymerase II and a bHLH domain that has been shown to be involved in DNA binding [53]. Both the bHLH domain and the C-terminus of these proteins can mediate homo- or heterodimerization of bHLH proteins. Similar to the C-terminal part of MYB proteins, the N-terminal part of bHLH proteins is more variable.

The third component of the MBW complex, participating in flavonoid/anthocyanin biosynthesis, is the WDR protein. These proteins are generally characterized by WD40 motifs of about 40–60 amino acids that typically end with a WD dipeptide (reviewed by [64, 65]). WDR proteins may assist the formation of stable protein complexes, serve as docking platforms/rigid scaffolds for protein-protein interactions and are thought to have no DNA-binding activity. Similar to the bHLH proteins in the MBW complex, WDR proteins that regulate the flavonoid pathway can also coordinate other regulatory networks, such as *Arabidopsis* AtTTG1 that controls trichome and root hair formation as well as seed coat development [66].

Recent advances in sequencing and computational technologies have greatly facilitated the study of non-model, wild and emerging new crop plants and can play key roles in understanding the biosynthetic pathways for novel bioactive compounds. The genetic resources and tools we have developed are available *via* the web-based transcriptome sequence database BacHBerryGEN [67] and its BLAST portal [68]. Gene expression studies during fruit ripening can be investigated using the newly developed BacHBerryEXP expression browser [69] in two *Rubus* species. As proof of concept, we cloned and conducted the functional analysis of *Myb*, *bHLH* and *WDR* genes involved in regulating anthocyanin biosynthesis in a wild and a cultivated *Rubus* species, using the transcriptomic tools generated in this study. We also investigated transcript expression patterns of genes involved in flavonoid biosynthesis at three fruit developmental stages in wild blackberry (*R. genevieri*) and cultivated red raspberry (*R. idaeus* cv. Prestige).

## Results and discussion

### Transcriptome sequencing and de novo assembly

We conducted de novo assemblies of one leaf and 16 fruit transcriptomes from 13 wild and cultivated berry fruit species. These species belong to eight plant genera and seven families: Berberidaceae (*B. buxifolia*), Caprifoliaceae (*L. caerulea*), Elaeocarpaceae (*A. chilensis*), Ericaceae (*C. album*, *V. corymbosum*, *V. uliginosum*), Grossulariaceae (two cultivars of *R. nigrum*), Rosaceae (three species including two cultivars of *R. idaeus*, *R. genevieri*, *R. vagabundus*) and Myrtaceae (*U. molinae*) that are dispersed over seven orders and three clades in the plant kingdom; Eudicots (three species), Eudicots-Asterids (four species) and Eudicots-Rosids (six species) (Table 1, Additional file 1: Table S1 and Additional file 2: Figure S1). Ploidy levels varied from diploid (*R. idaeus* and *V. corymbosum*) to tetraploid for *B. buxifolia*, *V. uliginosum*, *R. genevieri*. Fruits and leaves utilised for transcriptome analysis were collected by members of the BacHBerry Consortium [26] in Chile, China, Portugal, Russia and the UK (Additional file 1: Table S1). The species that were used for RNA-seq were either woody deciduous shrubs (Asterids: *L. caerulea*, *Vaccinium* spp., Eudicots: *Ribes* spp. and Rosids: *Rubus* spp.), evergreen shrubs (Eudicots: *B. buxifolia* and Rosids: *U. molinae*), an evergreen dioecious tree (Rosids: *A. chilensis*) and a shrub (Asterids: *C. album*). Several berries and fruits such as blueberries, blackcurrants and raspberries are widely cultivated; whereas the distribution of the other species is mostly restricted to their native habitats, for example, *A. chilensis* and *U. molinae* grow in their native terrains, Chile and Argentina, as well as in New Zealand and Australia; *R. genevieri* grows only in its natural habitat, Portugal; *V. uliginosum* grows in cool temperate regions of the Northern Hemisphere and *C. album* grows on the Atlantic coast of France and the Iberian Peninsula.

**Table 1:** Plant species and tissue used for transcriptome sequencing

Latin name	Common name	Plant material	Source^a^
*Aristotelia chilensis*	Maqui berry	fruit (ripe)	PUC, CL
*Berberis buxifolia*	Calafate	fruit (ripe)	PUC, CL
*Corema album*	Portuguese crowberry	leaf	IBET, PT
*Lonicera caerulea* (S322–3)	Blue honeysuckle	fruit (ripe)	VIR, RU
*Ribes nigrum* cv. Ben Hope	Blackcurrant	fruit (ripe)	JHI, UK
*Ribes nigrum* var. *sibiricum* cv. Biryusinka	Blackcurrant	fruit (ripe)	VIR, RU
*Rubus genevieri*	Blackberry (wild)	fruit (three ripening stages)	IBET, PT
*Rubus idaeus* cv. Octavia	Red raspberry	fruit (ripe)	JHI, UK
*Rubus idaeus* cv. Prestige	Red raspberry	fruit (three ripening stages)	JHI, UK
*Rubus vagabundus*	Blackberry (wild)	fruit (ripe)	IBET, PT
*Ugni molinae*	Strawberry myrtle	fruit (ripe)	PUC, CL
*Vaccinium corymbosum*	Blueberry	fruit (ripe)	IBET, PT
*Vaccinium uliginosum*	Bog bilberry	fruit (ripe)	IBCAS, CN

^a^PUC: Pontificia Universidad Católica de Chile, Macul, Chile,(CL); IBET: Instituto de Biologia Experimental e Tecnológica, Oeiras, Portugal (PT); VIR: N. I. Vavilov Research Institute of Plant Industry, Petersburg, Russia (RU); JHI: The James Hutton Institute, Invergowrie, United Kingdom (UK); IBCAS: Institute of Botany, The Chinese Academy of Sciences, Beijing, China (CN)

The majority of the berry fruit species that were used for RNA sequencing and analysis (Table 1) lacked an available reference genome sequence, therefore, de novo assembly of the Illumina reads was carried out for each species using Trinity software. Ten transcriptomes were assembled from RNA-seq data derived from a single cDNA library corresponding to ripe/mature fruits for gene identification purposes. Furthermore, six transcriptomes were assembled from RNA sequences taken at three different stages during fruit development and ripening (green/unripe, immature/intermediate ripe and mature/ripe fruit) of two *Rubus* species, using three cDNA libraries per stage to enable quantitative analysis of gene expression levels. To allow comparisons to vegetative tissues and due to a predicted high content of polyphenols in leaves, a leaf transcriptome was also prepared for a single species (*C. album*) for qualitative analysis. The transcriptome datasets are presented in Table 2 and complementary information is provided in Additional file 3: Table S2 and Additional file 4: Table S3. The online BacHBerryGEN repository database [67] and its BLAST portal [68] were developed to allow mining of the transcriptomic data of the 13 wild and cultivated berry fruit species.

**Table 2:** Summary of RNA-seq and de novo transcriptome assemblies of 13 berry fruit species

Plant species	Total number of raw reads	Total transcripts	Total assembled bases of transcripts	N50 length of transcripts	Overall read mapping rate (%)
*A. chilensis*	397,707,372	110,619	103,522,516	1526	84.6
*B. buxifolia*	444,362,698	736,393	488,614,277	1569	80.3
*C. album*	353,604,932	262,440	224,462,635	1408	91.1
*L. caerulea* (S322–3)	397,214,254	189,029	156,110,849	1345	88.6
*R. nigrum* cv. Ben Hope	336,479,242	145,906	129,471,515	1480	90.4
*R. nigrum* var. *sibiricum* cv. Biryusinka	393,665,630	186,129	141,064,478	1223	86.2
*R. genevieri*	1,040,224,680	286,262	222,576,819	1217	85.5
*R. idaeus* cv. Octavia	505,754,030	290,768	287,835,663	2214	90.5
*R. idaeus* cv. Prestige	1,064,858,518	155,094	149,987,271	1701	92.0
*R. vagabundus*	390,608,452	103,169	105,970,136	1565	85.5
*U. molinae*	405,024,920	138,456	166,588,685	1952	86.7
*V. corymbosum*	373,159,882	128,351	125,401,104	1519	80.4
*V. uliginosum*	375,778,718	703,066	422,097,427	1287	82.6

### Phylogenetic analysis and estimation of species divergence time

**Fig. 1:**
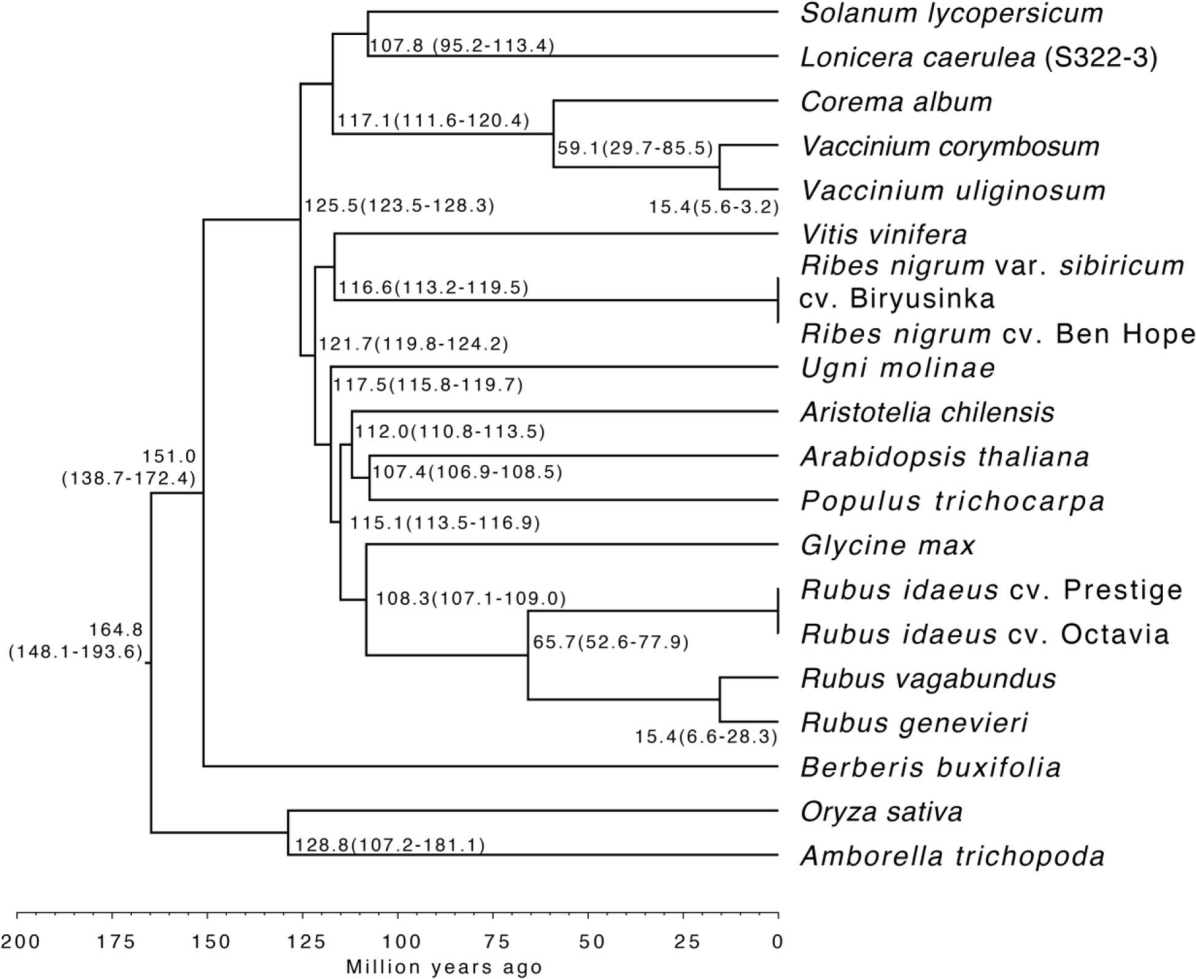
Phylogenetic analysis and estimation of species divergence time among 20 Angiosperm species. The twelve berry fruit transcriptomes and a berry leaf transcriptome were aligned together with the genome sequences of seven reference plant species (*A. thaliana*, *A. trichopoda*, *G. max*, *O. sativa*, *P. trichocarpa*, *S. lycopersicum* and *V. vinifera*) using single-copy gene orthologues (205). The estimated times of divergence are indicated at the tree nodes with the error values in parenthesis in million of years (My). The divergence time line is shown below the tree (in My)

We analysed the phylogenetic relationship of the twelve berry fruit transcriptomes and one leaf transcriptome together with the genome sequences of seven reference species. This included *(i)* four species classified among the Angiosperms/Eudicots/Rosids (*A. thaliana*, *Populus trichocarpa*, *Glycine max* and *V. vinifera*), *(ii)* a berry species that belongs to Angiosperms/Eudicots/Asterids (*S. lycopersicum*), *(iii)* an evergreen shrub that branches out at the base of the flowering plants *(Amborella trichopoda)* and *(iv)* a monocotyledonous species (Angiosperms/Monocots/Commelinids: *Oryza sativa*). In these 20 species, 56,232 gene families were identified using gene family clustering, of which 5387 were shared by all species and 205 of these shared families were single-copy gene families. The single-copy gene orthologues of the 20 species underwent homology searches to produce a super alignment matrix for the assembly of a phylogenetic tree (Fig. 1). The branching order displayed in the tree reflected the expected phylogenetic group classification for the clades, orders and families of the Angiosperms with members of the Rosids clade (*A. chilensis*, *R. genevieri*, *R. idaeus*, *R. vagabundus*, *U. molinae*, *A. thaliana*, *P. trichocarpa*, *G. max* and *V. vinifera*) and the clade of the Asterids (*C. album*, *L. caerulea*, *V. corymbosum*, *V. uliginosum* and *S. lycopersicum*) clustering together with an estimated time of divergence between the two clades of about 125 million years (My). Among the Rosids, *U. molinae* and *A. chilensis* separated from the Brassicales (*A. thaliana*) about 112–117 My ago, whereas the different *Rubus* spp. diverged about 66 My ago from the Fabales (*G. max*). *R. nigrum* spp. (Saxifragales) diverged about 117 My ago from the Vitales (*V. vinifera*), an order that represents an outgroup amongst Rosids. Among the Asterids, the Ericales separated from *L. caerulea* and *S. lycopersicum* approximately 117 My ago, while *Vaccinium* spp. (Ericales) diverged about 59 My ago from *C. album*. *B. buxifolia* (Ranunculales) split approximately 151 My ago from the other Eudicot orders. The monocot *O. sativa* is grouped outside the dicotyledonous species and diverged approximately 165 My ago. *A. trichopoda* represents a basal group of the Angiosperms that diverged about 129 My ago from the flowering plants.

### Homology-based mining of candidate genes encoding enzymes involved in phenylpropanoid biosynthesis, particularly flavonoid biosynthesis

As a proof of concept, we used the transcriptome sequences developed in this study to identify candidate genes involved in phenylpropanoid biosynthesis, a pathway known to be very active in berry fruits. To identify transcripts encoding enzymes involved in the general phenylpropanoid pathway, its flavonoid branch as well as in the modification and decoration of its flavonoid products and to identify candidate regulatory genes, MassBlast [70] and the TBLASTN algorithm-based BacHBerryGEN BLAST server [68] were used with search parameters of ‘expect score cut-off’ of 1e-10, an open reading frame (ORF) length of a minimum of 100 amino acids (aa) and aa identity greater than 40% in the alignments.

Key plant enzymes involved in the general phenylpropanoid biosynthetic pathway and their corresponding sequences (60) including 23 experimentally validated genes from different plant species were used in a targeted search approach to mine the different transcriptomes for homologous transcripts encoding phenylalanine ammonia lyase (PAL), cinnamate 4-hydroxylase (C4H), 4-coumarate CoA ligase (4CL), chalcone synthase (CHS), chalcone isomerase (CHI), flavanone 3-hydroxylase (F3H), flavonoid 3′-hydroxylase (F3′H), flavonoid 3′,5′-hydroxylase (F3′5′H), flavonol synthase (FLS), dihydroflavonol 4-reductase (DFR), anthocyanidin synthase (ANS), anthocyanidin reductase (ANR), leucoanthocyanidin reductase (LAR), flavone synthase (FNS) and stilbene synthase (STS). These BLAST searches are detailed in Additional file 5: Table S4.

Published sequences from a total of 68 regulatory proteins (45 MYB TFs, 18 bHLH TFs and five WDRs) and 120 modifying and decorating enzymes (18 acyltransferases, 31 glucosyltransferases, 29 methyltransferases, 26 hydroxylases, nine reductases, two aurone synthases, two dehydrogenases, two dehydratases and one dirigent protein) from a range of plant species were also used in BLAST searches against the transcriptome sequences of the 13 species. Detailed BLAST search results are presented in Additional file 5: Table S4.

In total, 1248 sequences homologous to regulatory genes and 5150 sequences homologous to enzymes of the general phenylpropanoid pathway and its decoration and modification were identified from the different RNA-seq datasets (Table 3 and Additional file 5: Table S4). Multiple candidates encoding each type of decorating enzyme were identified in each transcriptome. Amongst putative modifying and decorating enzymes, 19 acyltransferases, 96 glucosyltransferases, 39 methyltransferases, 91 hydroxylases, 55 reductases, six aurone synthases, 16 dehydrogenases, 17 dehydratases and two dirigent protein candidate genes were identified on average per species. Generally, at least two to three homologues per decorating/modifying enzyme could be found in every species with glucosyltransferases and hydroxylases being the most abundant decorating enzymes. Different cultivars of *R. idaeus* (cv. Octavia and cv. Prestige) and *R. nigrum* (cv. Ben Hope and var. *sibiricum* cv. Biryusinka) exhibited similar patterns of homologue distribution amongst the transcripts encoding the different types of enzymes. *R. genevieri*, *V. uliginosum*, *B. buxifolia* and to a lesser extent *L. caerulea* and *C. album* exhibited a greater average number of homologues than the other species. This abundance of homologues is likely due to the higher ploidy levels of these accessions.

**Table 3:** Transcriptome analysis of berry fruit species for genes involved in the general phenylpropanoid biosynthetic pathway, its regulation as well as modification and decoration of its products

Plant species	Core pathway, decorating and modifying enzymes^a^	Pathway regulators^a^
*A. chilensis*	337	69
*B. buxifolia*	517	146
*C. album*	465	104
*L. caerulea* (S322–3)	415	112
*R. nigrum* cv. Ben Hope	344	82
*R. nigrum* var. *sibiricum* cv. Biryusinka	371	95
*R. genevieri*	535	130
*R. idaeus* cv. Octavia	348	76
*R. idaeus* cv. Prestige	350	107
*R. vagabundus*	290	50
*U. molinae*	281	71
*V. corymbosum*	320	77
*V. uliginosum*	577	129

^a^Number of candidate genes

Comparison of BLAST search outputs of blackberry, blueberry, Maqui berry and strawberry myrtle also showed that transcripts encoding methyltransferases were the most conserved enzymes, with half to three-quarters of the sequences exhibiting high aa similarity levels, with the exception of blueberry (44.8% of genes). Reductases were also highly conserved between these species. In contrast, acyltransferases and glucosyltransferases were rarely detected with high levels of aa similarity. Approximately a third of the hydroxylases and glucosyltransferases were detected with high levels of aa similarity.

Amongst candidate regulatory genes controlling flavonol, anthocyanin or PA biosynthesis, on average, 85 *Myb*, five *bHLH* and four *WDR* candidate regulatory genes related to the phenylpropanoid pathway were detected per species.

In addition to the gene mining of the phenylpropanoid pathway, protein-coding sequences were predicted and functionally annotated in the transcriptomes of all the 13 species. The annotated ORFs for the transcriptomes of *R. genevieri* and *R. idaeus* cv. Prestige are shown in Additional file 6: Table S5.

### Regulatory genes of the anthocyanin biosynthetic pathway isolated from *R. genevieri* and *R. idaeus* cv. Prestige

Using the transcriptomic data of *R. genevieri* (abbreviated as Rg) and *R. idaeus* cv. Prestige (abbreviated to Ri), several candidate regulatory genes of the anthocyanin biosynthetic pathway were identified in both species, cloned and characterised. The protein query sequences used for mining the fruit transcriptomic data were *(1) M. domestica* MdMYB10 as a representative member of the R2R3-type MYB gene subgroup 6 (SG6) family, responsible for the regulation of anthocyanin and PA biosynthesis [54, 71] which led to the isolation of *RgMyb10* and *RiMyb10*; *(2) A. thaliana* AtMYB12 as a member of the R2R3-type MYB TFs of SG7 that control the activation of flavonol and flavone synthesis [54] which resulted in the isolation of *RgMyb12* and *RiMyb12*; *(3)* bHLH TF homologues of *P. hybrida* ANTHOCYANIN1 (SG IIIf-1; PhAN1-type bHLHs) and *A. majus* DELILA (SG IIIf-2; AmDEL-type bHLHs) involved in the flavonoid/anthocyanin biosynthesis and epidermal cell fate [63] which generated the cloned RT-PCR products of *RgAn1/RiAn1* and *RgDel/RiDel* respectively; as well as *(4) M. domestica* TRANSPARENT TESTA GLABRA 1 (MdTTG1) as a WD40 protein homologue which led to the cloning of *RgTTG1* and *RiTTG1* (Table 4, Additional file 7: Table S6 and Additional file 8: Table S7).

**Table 4:** Cloning and functional analysis of regulatory genes of the phenylpropanoid pathway in *R. genevieri* and *R. idaeus* cv. Prestige

Species	Gene function (Subgroup)	Cloned gene^a^	Transient / Stable transformation^b^
*R. genevieri*	R2R3-type MYB TF (SG6)	*RgMyb10* (654 nt/217 aa; KY111315)	T / S
	R2R3-type MYB TF (SG7)	*RgMyb12* (1296 nt/431 aa; KY111316)	T / S
	PhAN1-like bHLH TF (SG IIIf-1)	*RgAn1-1* (2100 nt/699 aa; KY123749)	T / -
	PhAN1-like bHLH TF (SG IIIf-1)	*RgAn1-2* (2103 nt/700 aa; KY123750)	T / S
	PhAN1-like bHLH TF (SG IIIf-1)	*RgAn1-3* (2100 nt/699 aa; KY123751)	T / S
	AmDEL-like bHLH TF (SG IIIf-2)	*RgDel* (1929 nt/642 aa; KY111317)	T / S
	WD40-repeat protein	*RgTTG1-1* (1041 nt/346 aa; MH460860)	T / S
	WD40-repeat protein	*RgTTG1-2* (1041 nt/346 aa; MH460861)	T / -
*R. idaeus* cv. Prestige	R2R3-type MYB TF (SG6)	*RiMyb10* (654 nt/217 aa; KY111313)	T / S
	R2R3-type MYB TF (SG7)	*RiMyb12* (1272 nt/423 aa; KY111314)	T / S
	PhAN1-like bHLH TF (SG IIIf-1)	*RiAn1* (2100 nt/699 aa; KY111320)	T / S
	AmDEL-like bHLH TF (SG IIIf-2)	*RiDel-1* (1926 nt/641 aa; KY111318)	T / -
	AmDEL-like bHLH TF (SG IIIf-2)	*RiDel-2* (1929 nt/642 aa; KY111319)	T / -
	WD40-repeat protein	*RiTTG1* (1035 nt/344 aa; MH460862)	T / -

^a^Cloned gene name (nucleotide / amino acid length; GenBank accession number)

^b^Transient assays (T) / stable transformation (S) were conducted in *N. benthamiana*

The cloned *Myb* genes were analysed for the presence of sequences encoding several known conserved aa motifs of R2R3-type MYB TFs (Additional file 9: Figure S2). The MYB domain consisting of the imperfect repeats R2 and R3 with regularly spaced tryptophan residues (R2 [−W-(x_19_)-W-(x_19_)-W-] … R3 [−F/I-(x_18_)-W-(x_18_)-W-] [54]) was highly conserved in the N-terminus of the four *Rubus* MYB TFs. Several regulators of the anthocyanin and PA pathways have been shown to contain an additional aa signature motif for bHLH interaction ([D/E]Lx_2_[R/K]x_3_Lx_6_Lx_3_R [61]) within the R3 repeat. The bHLH interaction motif and the anthocyanin-related SG6 MYB motif were present in the putative SG6 members RgMYB10 and RiMYB10 (Additional file 9: Figure S2) but were not present in the predicted SG7 homologues, RiMYB12 and RgMYB12. RgMYB10 and RiMYB10 also possessed domains present in other anthocyanin promoting MYB TFs such as the anthocyanin-related SG6 MYB motif of [R/K]Px[P/A/R]x_2_[F/Y] which lies downstream of the MYB domain as well as the small conserved ‘box A’ motif ([A/S/G]NDV) in the R3 repeat of the DNA binding domain [72]. In contrast, the SG7 homologues RiMYB12 and RgMYB12 contained a ‘box A’ motif ([D/E]N[E/D][I/V] [72]) characteristic of SG7 regulators in their R3 repeat. The conserved motif of flavonol synthesis-related SG7 R2R3-type MYBs (GRTxRSxMK [71] or [K/R][R/x][R/K]xGRT[S/x][R/G]x_2_[M/x]K [73]) was modified slightly in RiMYB12 (KxRx_3_GRTSRx_2_MK) and RgMYB12 (KRRx_3_GRNSRx_2_MK) (Additional file 9: Figure S2). This SG7 motif is also only partially conserved in the tomato SlMYB12 and grapevine VvMYB12 homologues [73]. The motif designated SG7-2 ([W/x][L/x]LS [73]) was fully conserved at the C-terminal ends of both RgMYB12 and RiMYB12 (Additional file 9: Figure S2). No motifs associated with MYBs that act as transcriptional repressors such as members of SG4 that contain an EAR (ethylene response factor-associated amphiphilic repression) motif (LxLxL or DLNxxP [74]) or the TLLLFR repression motif were found amongst the RgMYB and RiMYB SG6 TFs.

The *Rubus Myb10* homologues (Table 4) were very similar with 92%/94% aa identity/similarity between RgMYB10 and RiMYB10. RiMYB10 was identical to a homologue characterized from another *R. idaeus* cultivar, cv. Latham (Accession no. EU155165) [72]. Another *Myb10* homologue cloned from a *Rubus* hybrid cultivar (Accession no. JQ359611) has an aa identity/similarity of 89–91%/94% with Rg/RiMYB10. RuMYB1 from a cultivated blackberry (*Rubus* sp. var. Lochness) [75] shared 97% aa identity with RgMYB10 from wild blackberry and an aa identity/similarity of 93%/96% with the RiMYB10 from cultivated red raspberry. The *Myb12* homologues of both *Rubus* species (Table 4) were also closely related (aa identity/similarity of 89%/91%). Phylogenetic analysis of the *Rubus* and several other R2R3-type MYB TFs showed clear separation of the flavonoid MYB regulators into two distinct clades (equivalent to SG6 and SG7 in *A. thaliana* [71]; Additional file 9: Figure S2).

Of the seven *bHLH* homologues cloned (Table 4), three encoded isoforms of *RgAn1* (termed *RgAn1-1*, *RgAn1-2*, *RgAn1-3* with 99% aa identity among the isoforms), *RiAn1*, *RgDel* and two isoforms of *RiDel* (named *RiDel-1* and *RiDel-2* that shared 99% identity at the aa level) had the general structure of flavonoid bHLH TFs (being about 600 aa in length, reviewed by [53]) (Additional file 10: Figure S3). The bHLH TFs each contained a N-terminal MYB-interacting region (MIR, aa 1 to approximately aa 200), a domain of interaction with WD40 and/or with the RNA polymerase II *via* the acidic domain (AD) (WD40/AD, extending from approximately aa 200 to aa 400) and a bHLH domain (approximately 60 aa, basic[~ 17 aa]-Helix 1[~ 16 aa]-Loop[~ 6–9 aa]-Helix 2[~ 15 aa]). The characteristic H-E-R aa motif (−H-(x_3_)-E-(x_3_)-R- [63]) within the basic part of the bHLH domain is preserved in all cloned bHLH TFs of the two *Rubus* species. The AmDEL homologues RgDEL and RiDEL-1/RiDEL-2 (SG IIIf-2) were closely related with a pairwise aa identity of 98% while the SG IIIf-1 PhAN1 homologues of *R. genevieri* (RgAN1-1 to RgAN1-3) and *R. idaeus* cv. Prestige (RiAN1) were slightly more diverged showing a 96–97% pairwise aa identity. Phylogenetic analysis showed clustering of the different *Rubus* bHLH TFs together with other plant bHLH homologues in two conserved clades of bHLH regulatory proteins (SGIIIf-1: PhAN1/AtTT8 clade and SGIIIf-2: AmDEL/PhJAF13 clade; Additional file 10: Figure S3).

When analysing *WDR* homologues, *RgTTG1* (two isoforms named *RgTTG1-1* and *RgTTG1-2* that share a 99% identity at the aa level) and *RiTTG1* were identified. These contained seven WD40 repeats (36–54 aa) as predicted using the WDSPdb database for WD40-repeat proteins [76, 77] (Additional file 11: Figure S4). Among these, four WD40 repeats corresponded to the domains previously identified in WDR proteins associated with anthocyanin biosynthesis [78]. The characteristic ‘WD’ dipeptide motif at the C-terminus of each WD40 repeat as well as the GH dipeptide delimiting the N terminus of several WD40 motifs were not fully conserved in many plant WDR homologues including those identified from *Rubus* (Additional file 11: Figure S4). Similarly, a D-H-[S/T]-W tetrad motif involved in the hydrogen bond network stabilising the propeller-like structure of certain WD40 proteins (reviewed by [79]) was conserved only partially between different WD40 proteins expressed in berry fruits. *RiTTG1* was closely related to AtTTG1 (aa identity/similarity of 80%/88%) and MdTTG1 (aa identity/similarity of 92%/96%) whereas the two *RgTTG1* isoforms were more distantly related (aa identity/similarity of 61%/78% with MdTTG1 and aa identity/similarity of 64%/79% with AtTTG1). The aa sequence of *RiTTG1* was identical to that of another cultivar (*R. idaeus* cv. Moy TTG1, Accession no. HM579852). The phylogenetic analysis of RiTTG1 and RgTTG1 with other plant WDR homologues is shown in Additional file 11: Figure S4.

Candidate transcripts that are highly homologous to the *Myb*, *bHLH* and *WDR* regulatory genes cloned and functionally characterized in this study (Table 4) were identified in all the 13 berry fruit species and are listed in Additional file 12: Table S8.

### Functional characterisation of regulatory genes of the anthocyanin biosynthetic pathway isolated from *R. genevieri* and *R. idaeus* cv. Prestige

**Fig. 2:**
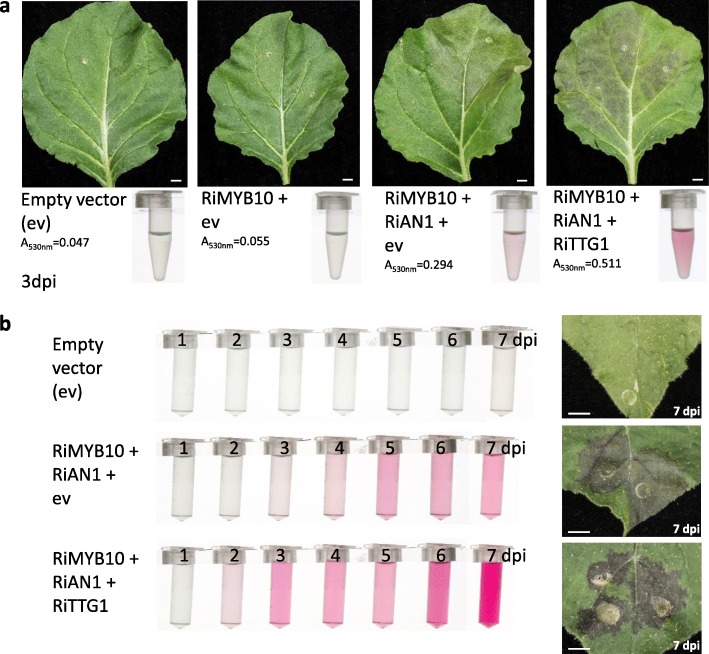
Production of anthocyanins in leaves of *N. benthamiana* cv. NT following transient overexpression of *Rubus Myb* and *bHLH* regulatory genes in the presence or absence of a WDR component (TTG1). **a** Transient overexpression of flavonoid regulatory genes in *N. benthamiana* leaves at 3 days post infiltration (dpi) in comparison to the empty vector (ev) construct. The methanol extracts from each infiltration combination are presented below the infiltrated leaf used for extraction (i.e., 1.8-cm diameter leaf disc in 2 ml methanol: water: HCl (80:20:1, v/v/v). Bar = 1 cm. **b** Methanol extracts from *N. benthamiana* leaves (1.8-cm diameter leaf disc in 2 ml methanol: water: HCl (80:20:1, v/v/v) transiently expressing *Rubus* flavonoid regulatory genes with or without a WDR co-factor from 1 to 7 dpi. Extracts represent average absorbance values at 530 nm from eight leaf discs per time point. Leaf expression is shown at 7 dpi. Bar = 0.5 cm

To characterise the MYB, bHLH and WDR proteins functionally (Table 4), transient and stable expression studies were carried out in two accessions of *N. benthamiana*, a laboratory isolate (JIC-LAB) and an ecotype from the Australian Northern Territory (NT) [80]. Agroinfiltrations were performed with the candidate regulatory genes from *Rubus* on their own and in combinations with putative partners (Additional file 13: Figure S5). The anthocyanin biosynthetic pathway is generally not active in leaves of *N. benthamiana*, although colourless flavonols are produced. Inoculated on their own, *Rubus Myb10*, *Myb12*, *bHLH* and *WDR* genes (Fig. 2 and Additional file 13: Figure S5) did not induce red-purple pigmentation observable visually in agroinfiltrated leaf patches of *N. benthamiana*. The lack of anthocyanin production in the infiltrated *N. benthamiana* leaves infiltrated with these genes was confirmed by analysing the methanol: water: HCl (80:20:1, v/v/v) extracts of leaf discs from infiltrated areas (Additional file 13: Figure S5).

However, when combined with most of the cloned *Rubus* bHLH TFs, inoculation of the *Rubus Myb10* genes induced a strong red-purple colouration in infiltrated leaf patches to a level easily detectable by the naked eye (Fig. 2 and Additional file 13: Figure S5). For example, the three *RgAn1*-type *bHLH* isoforms from *Rubus* gave rise to similar pigmentation intensities when co-infiltrated with *RgMyb10*. RgAN1-2 was often the most effective bHLH partner among the RgAN1 isoforms. In contrast, the AmDEL-type RgDEL TF did not induce visual anthocyanin production in *N. benthamiana* leaves in combination with RgMYB10 or RiMYB10 (Additional file 13: Figure S5) suggesting that RgDEL might not be functional in activating anthocyanin biosynthesis or might have another regulatory role. Mixes of *RgMyb10* and *RgDel* supplemented with either *RgMyb12* and/or *RgTTG1* also did not lead to visual pigmentation in leaves nor in methanol extracts of leaves (Additional file 13: Figure S5). In contrast, RiDEL was able to interact with RiMYB10 and RgMYB10 to induce anthocyanin biosynthesis and appeared to be as effective as RiAN1 in this partnership (Additional file 13: Figure S5). These results suggested that the DEL proteins from different species of *Rubus* that share a 98% aa identity (11 aa variations) have differential abilities to induce anthocyanin biosynthesis. Among the aa differences, only a few occur in (highly) conserved regions of plant bHLH TF (Additional file 10: Figure S3). For example, RgDEL that is unable to initiate anthocyanin production with Ri/RgMYB10 in contrast to RiDEL, contains an arginine at position 150 compared to lysine within the MIR domain and, in the WD40/AD domain, differences at aa positions 247, 248 and 251 (Additional file 10: Figure S3). These differences could be responsible for the lack of anthocyanin synthesis in *RgDel* and *Ri/RgMyb10* co-infiltrated leaves.

RiMYB10 interacted with both the PhAN1-like RiAN1 and the two AmDEL-like bHLH homologues, RiDEL-1 and RiDEL-2, with the two RiDEL proteins producing similar pigmentation levels in combination with RiMYB10 (Additional file 13: Figure S5). However, there were noticeable differences in the intensity of pigmentation accumulating over time in these assays; anthocyanin production induced by RiMYB10 co-expressed with RiAN1 was weak early after infiltration and peaked 5 days post infiltration (dpi). In contrast, anthocyanin accumulation of RiMYB10 plus RiDEL peaked at 4 dpi at which time the leaf tissue often started to deteriorate in the highly anthocyanin-enriched areas. Similarly, RgMYB10 produced strong red pigmentation earlier with RiDEL-2 (at 3–4 dpi) than when co-infiltrated with *RgAn1*. This suggested that RiMYB10 and RgMYB10 might interact preferentially with the different bHLH homologues in a time/phase-dependent manner or that bHLH TFs possess different binding affinities towards their MYB partner leading to differences in the rate of forming the MBW complex. Alternatively, these phylogenetically distinct bHLH TFs might operate *via* a hierarchical mechanism, as has been suggested in regulating anthocyanin biosynthesis [60, 81]. For example, an AmDEL-type bHLH homologue (SG IIIf-2) might activate the expression of a PhAN1-type bHLH homologue (SG IIIf-1) for subsequent MBW complex formation, and analysis in *N. benthamiana* has provided experimental evidence to support this model [60, 81].

It has been suggested that anthocyanin promoting MYB TFs display selectivity in their interactions with different bHLH partners [82]. In several anthocyanin regulatory systems, it has been shown that a MYB10-like TF alone can stimulate anthocyanin production in *N. tabacum* and/or *N. benthamiana* [83–85], although always to a lesser extent than when co-expressed with a bHLH TF partner. However, R2R3-type MYB TFs from Rosaceous species, including a RiMYB10 homologue [72], three peach *Myb10* genes [86] as well as a strawberry MYB10 homologue [87] could trigger pigmentation in *N. tabacum* and/or *N. benthamiana* leaves only in combination with an added bHLH partner. Overall, the most parsimonious explanation seems to be that where MYB SG6 proteins can stimulate anthocyanin production on their own in transient assays in *N. tabacum* and/or *N. benthamiana,* they use endogenous bHLH TFs and WD40 proteins expressed in *N. tabacum* or *N. benthamiana* leaves as partners in the MBW complex(es). Those SG6 TFs that require an added bHLH for anthocyanin induction likely require specific interacting bHLH partners for pigment formation, either in a hierarchical regulatory cascade or directly in the MBW complex that activates the expression of the genes encoding the enzymes of anthocyanin biosynthesis. Anthocyanin regulatory systems might vary between plant families/orders as they do for monocot and dicot species (reviewed by [88]) and might also involve selective binding to regulatory elements in the promoters of their target genes [89].

Agroinfiltration of *Rubus Myb12* TF genes, *RgMyb12* or *RiMyb12*, together with *Rg/RiMyb10* and a *bHLH* gene (*Rg/RiAn1* or *RiDel*) generally enhanced anthocyanin production in leaves of *N. benthamiana* (Additional file 13: Figure S5), as seen in earlier studies with AtMYB12, AmRos1 and AmDEL in tomato [90].

HPLC analysis of methanol: water: HCl extracts (80:20:1, v/v/v) from leaves of the *N. benthamiana* JIC-LAB isolate infiltrated with different combinations of *Rubus Myb* and *bHLH* homologues showed that the main anthocyanin compound produced corresponded to delphinidin-3-rutinoside, with maximum absorption at 530 nm. Flavonoids and other phenolics detected at about 350 nm included the flavonol myricetin-3-O-rutinoside (MyrRut; generally found in extracts from *Rubus* MYB12 co-expressing samples), myricetin (glucose)_2_ rhamnose (Myr(Glc)_2_Rha), kaempferol-3-O-rutinoside (KaeRut), kaempferol (glucose)_2_ rhamnose (Kae(Glc)_2_Rha), rutin (quercetin-3-O-rutinoside) and chlorogenic acids (CGA1 and CGA2). Delphinidin-3-rutinoside was also found to be the major product synthesized in *N. benthamiana* leaf tissues transiently overexpressing SG6 MYB (AmROS1) and bHLH (AmDEL) TFs [85].

To investigate the role of WDR proteins from *Rubus* in the MBW complex, transient assays in *N. benthamiana* leaves were carried out with the putative components of the *R. idaeus* MBW complex, RiMYB10, RiAN1 and RiTTG1. To score the amount of anthocyanins accumulated in infiltrated leaf patches in the presence or absence of a WDR co-factor over time, methanol: water: HCl extracts of leaf samples were analysed by absorbance at 530 nm. Anthocyanin accumulation increased approximately 4.3-fold between 4 and 7 dpi in the presence of a WDR component compared to an approximately 1.8-fold increase without a WDR co-factor and therefore, the addition of the WDR co-factor RiTTG1 almost doubled the anthocyanin content in *N. benthamiana* (JIC-LAB isolate) leaves infiltrated with RiMYB10 and RiAN1.

To examine the effect of a WDR co-factor in the infiltration mixes from very early stages post inoculation, anthocyanin accumulation was observed over 7 days, from 1 to 7 dpi in the response to agroinfiltration with *RiMyb10* and *RiAn1* in the NT accession of *N. benthamiana* (Fig. 2). At 1 dpi, anthocyanin formation was not visible by the naked eye nor detectable in methanol extracts of infiltrated leaves (eight leaf discs per treatment) and equalled to the one of the mock infiltrated leaves of avgA_530nm_ of 0.06 (+/− SE of 0.00) that remained largely unchanged (avgA_530nm_ of 0.05 to 0.07 (+/− SE of 0.00 to 0.01) over the time course. At 2 dpi, the effect of the addition of a WDR protein was already evident, as anthocyanin formation could be observed by the naked eye in *RiMyb10*, *RiAn1* and *RiTTG1*-co-infiltrated leaves and pigment formation was estimated in methanol extracts of leaf discs as avgA_530nm_ of 0.20 (+/− SE of 0.02). In contrast, samples lacking a WDR protein had similar anthocyanin levels to the control treatment (avgA_530nm_ of 0.07 (+/− SE of 0.00) at 2 dpi and required 3–4 dpi to reach similar pigmentation levels as those leaves infiltrated with the WDR co-factor had exhibited at 2 dpi. At 3 dpi, the MBW co-expression mixes showed up to 3–5-fold stronger anthocyanin production that plateaued around 3–4 dpi and accurate scoring of anthocyanin accumulation after 7 dpi became very hard. Visual observation of pigment formation also suggested that incorporating the *RgTTG1* isoforms in *RgMyb10* and *RgAn1-2* co-infiltration mixes increased (early) anthocyanin production. Overall, transient overexpression of a WDR protein enhanced the early accumulation of anthocyanins, leading to faster synthesis of pigments in vegetative tissues of *N. benthamiana* that normally do not synthesize coloured flavonoids (Fig. 2). The rapid formation of an ectopic MBW complex, when endogenous WDR homologues might not be accessible or are present at limiting levels might, therefore, boost the early induction of anthocyanin biosynthesis. Our data confirm a report on the induction of anthocyanin production in *N. tabacum* transient assays by the *Myrica rubra* MBW complex which was earlier and enhanced by the WDR component [91].

The wild accession of *N. benthamiana* from the Australian Northern Territories (NT) has been suggested to be particularly well-suited for anthocyanin-related studies [80]. Generally, anthocyanin pigmentation using berry fruit genes was visible in the NT accession well before (at 2 dpi) pigmentation could be observed by the naked eye in the JIC-LAB isolate (3–4 dpi). The yield of anthocyanins produced in the infiltrated leaves, as predicted by A_530nm_ absorbance values of methanol: water: HCl (80:20:1, v/v/v) extracts from leaf discs, was also far higher (minimally 2–3-fold higher) in the NT isolate than in the JIC-LAB accession. Use of the NT accession for infiltrations confirmed all our observations using the JIC-LAB strain of *N. benthamiana*.

### Functional analysis of candidate regulatory proteins in stable transformations of *N. benthamiana*

Stable (co)-transformations of *N. benthamiana* leaf and stem explants with *RiMyb10* or *RgMyb10* under the control of the constitutive CaMV 35S promoter led to anthocyanin induction with and without bHLH and/or WDR co-factors from the same species. Different levels of red pigmentation (varying from light red to dark red/purple) were initially observed in callus sectors of explants grown on selection media (Additional file 14: Figure S6). Anthocyanin pigmentation continued also into later stages of regeneration in leaves and stems of developing shoots. High levels of pigmentation were often associated with a severe delay in shoot development, shoot stunting and deformation as well as the absence of root formation. In the past, tomato plants overexpressing the grapevine R2R3 MYBs VvMYB5a and VvMYB5b showed also phenotypic alterations including dwarfism [92]. Similar to the transient expression studies, anthocyanin production was greatly enhanced in stably co-transformed tissue with *Rubus Myb10*, *bHLH* with or without *WDR* genes while a limited amount of red-purple pigmentation could be detected in tissues transformed with *Rg/RiMyb10* alone. Our data indicated that transient assays do not always reflect the metabolic changes observed in stable transformations, the latter being more sensitive indicators (at specific stages during regeneration) of the ability of regulatory genes to ectopically induce anthocyanin production. This could reflect some inherent suppression mechanism of anthocyanin biosynthesis in maturing leaves of *N. benthamiana*. *N. benthamiana* leaf and stem explants transformed with *RgMyb12* or *RiMyb12* alone, developed calli and shoots with a wild-type appearance as observed in AtMYB12 ectopic overexpression studies [56].

### Differential gene expression during fruit development and ripening in *R. genevieri* and *R. idaeus* cv. Prestige and expression patterns of genes related to anthocyanin biosynthesis

**Fig. 3:**
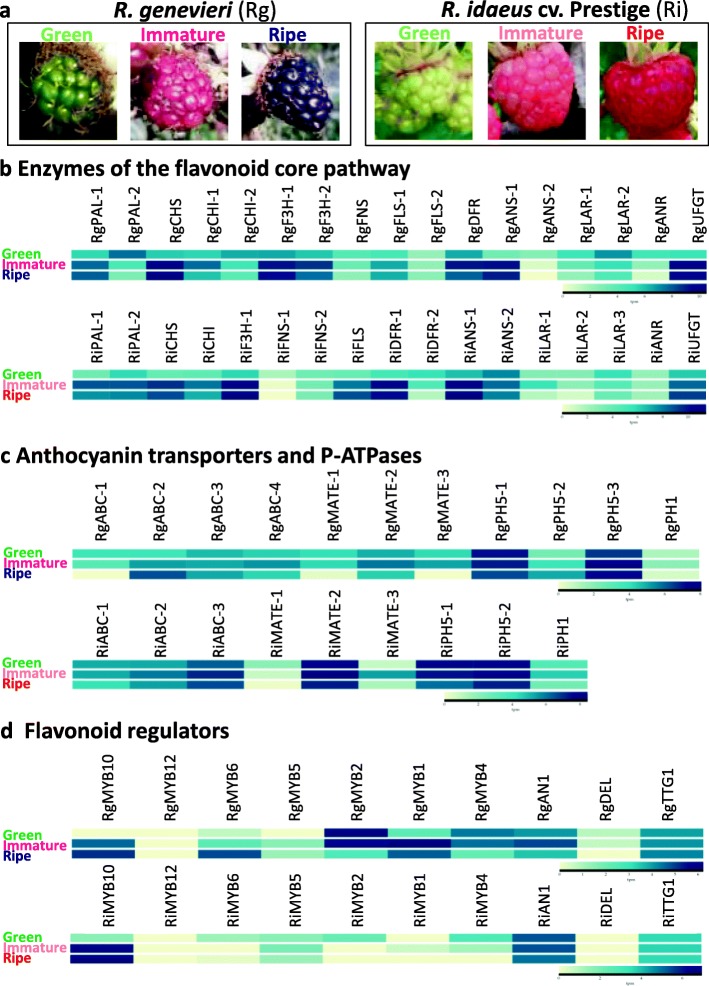
Transcriptome profiling of candidate genes encoding enzymes of the flavonoid core pathway, anthocyanin transporters, P-ATPases and flavonoid regulatory proteins during fruit maturation in *R. genevieri* (Rg) and *R. idaeus* cv. Prestige (Ri). **a** Gene expression was analysed in three developmental stages (unripe, immature and ripe fruits). **b** to **d** Candidate genes were identified *via* homology-based gene mining (BacHBerryGEN [68]) and expression patterns were visualized using the BacHBerryEXP expression browser (tpm and log_2_ values) [69]. **b** Candidate genes encoding enzymes of the phenylpropanoid core pathway and modifying proteins: *Top panel - R. genevieri* homologues: RgPAL-1 (TR124859|c0_g1_i1), RgPAL-2 (TR119394|c2_g1_i1), RgCHS (TR121228|c1_g2_i1), RgCHI-1 (TR87748|c0_g1_i2), RgCHI-2 (TR109085|c3_g1_i1), RgF3H-1 (TR65548|c1_g1_i2), RgF3H-2 (TR99162|c0_g1_i1), RgFNS (TR117515|c0_g1_i1), RgFLS-1 (TR82651|c1_g1_i1), RgFLS-2 (TR89606|c1_g1_i1), RgDFR (TR23878|c0_g1_i1), RgANS-1 (TR79533|c1_g1_i1), RgANS-2 (TR85881|c0_g1_i1), RgLAR-1 (TR97331|c0_g1_i1), RgLAR-2 (TR79474|c0_g3_i1), RgANR (TR77419|c0_g1_i1), RgUFGT (TR99106|c0_g1_i1); *Lower panel - R. idaeus* cv. Prestige homologues: RiPAL-1 (TR17637|c0_g1_i1), RiPAL-2 (TR60786|c0_g2_i1), RiCHS (TR38621|c0_g1_i3), RiCHI (TR60776|c0_g1_i1), RiF3H-1 (TR22747|c0_g2_i1), RiFNS-1 (TR17254|c0_g1_i1), RiFNS-2 (TR31274|c0_g1_i2), RiFLS (TR76353|c0_g1_i1), RiDFR-1 (TR26907|c0_g1_i1), RiDFR-2 (TR25484|c0_g1_i1), RiANS-1 (TR24906|c0_g1_i1), RiANS-2 (TR19248|c0_g2_i2), RiLAR-1 (TR24256|c0_g1_i1), RiLAR-2 (TR8288|c0_g1_i2), RiLAR-3 (TR58287|c1_g1_i1), RiANR (TR6460|c0_g1_i1), RiUFGT (TR3455|c0_g1_i1). **c** Candidate anthocyanin ABC and MATE transporters as well as P-ATPases. *Top panel - R. genevieri* homologues: RgABC-1 (TR71618|c2_g1_i1), RgABC-2 (TR72263|c2_g1_i5), RgABC-3 (TR114784|c2_g1_i2), RgABC-4 (TR73971|c3_g1_i3), RgMATE-1 (TR99523|c1_g1_i1), RgMATE-2 (TR81657|c3_g1_i1), RgMATE-3 (TR86341|c0_g1_i1), RgPH5-1 (TR72443|c2_g1_i1), RgPH5-2 (TR113411|c2_g1_i2), RgPH5-3 (TR72105|c0_g1_i1), RgPH1 (TR107023|c1_g2_i2); *Lower panel - R. idaeus* cv. Prestige homologues: RiABC-1 (TR41909|c1_g2_i1), RiABC-2 (TR66334|c1_g1_i2), RiABC-3 (TR27015|c1_g4_i3), RiMATE-1 (TR39949|c0_g1_i3), RiMATE-2 (TR11226|c0_g1_i1), RiMATE-3 (TR10226|c0_g1_i1), RiPH5-1 (TR570|c0_g1_i1), RiPH5-2 (TR41268|c0_g1_i1), RiPH1 (TR52475|c0_g1_i6). **d** Cloned and candidate *Rubus* regulatory proteins: *Top panel - R. genevieri* homologues: RgMYB10 (TR103098|c0_g1_i1), RgMYB12 (TR71550|c1_g1_i1), RgMYB6 (TR86812|c0_g1_i1), RgMYB5 (TR80732|c0_g11_i7), RgMYB2 (TR36560|c0_g1_i1), RgMYB1 (TR111295|c2_g2_i1), RgMYB4 (TR32557|c0_g1_i1), RgAN1 (TR110272|c1_g1_i1), RgDEL (TR110629|c1_g1_i1), RgTTG1 (TR29409|c0_g1_i1); *Lower panel - R. idaeus* cv. Prestige homologues: RiMYB10 (TR49283|c2_g2_i2), RiMYB12 (TR1036|c0_g1_i2), RiMYB6 (TR67691|c0_g1_i2), RiMYB5 (TR48317|c3_g1_i6), RiMYB2 (TR817|c0_g1_i2), RiMYB1 (TR75558|c0_g1_i1), RiMYB4 (TR16747|c0_g1_i1), RiAN1 (TR75681|c0_g1_i1), RiDEL (TR16024|c0_g1_i1) and RiTTG1 (TR7065|c0_g2_i1)

To study the gene expression levels during fruit ripening in the wild blackberry *R. genevieri* and the cultivated red raspberry *R. idaeus* cv. Prestige, the BacHBerryEXP expression browser [69] was developed using the RNA-seq data analysis and a visualization platform expVIP (expression Visualization and Integration Platform) [93]. The BacHBerryEXP browser uses the transcriptome data from three stages of fruit maturation for the two *Rubus* species (green fruit, intermediate/immature fruit and ripe fruit; Fig. 3a) and calculates gene expression levels by using the pseudoalignment tool Kallisto [94]. It displays either the expression units as raw counts or transcripts per million (tpm) and their log_2_ values that can be represented as heatmaps. BacHBerryEXP also contains a BLAST tool [95] to identify candidate transcript homologues for differential expression analysis of the two *Rubus* species.

To illustrate the transcriptional expression levels of key structural and regulatory genes related to the general phenylpropanoid pathway and anthocyanin biosynthesis, we conducted a homology-based search of candidate genes using the BacHBerryGEN BLAST server [68] and a set of characterised plant protein homologues. We identified a range of candidate transcripts encoding proteins associated with phenylpropanoid metabolism as well as the modification, decoration and transport of its flavonoid products in the two *Rubus* species. For most candidate genes, up to five transcripts, with the highest homology scores (but not necessarily a full-length transcript), were selected and their expression profiles were analysed during fruit maturation using BacHBerryEXP [69] (Fig. 3 and Additional file 15: Table S9). For anthocyanin regulators such as the *Rubus* R2R3-type MYB, bHLH and WDR homologues cloned in this study, the expression levels of the cloned transcripts were assessed (Fig. 3 and Additional file 15: Table S9).

The formation of anthocyanin pigments in ripening fruits involves the coordinated expression of genes encoding a series of enzymes in the phenylpropanoid pathway. Heatmaps representing the transcriptomic profiling of key flavonoid pathway and anthocyanin biosynthetic enzymes (PAL, CHS, CHI, F3H, FLS, FNS, DFR, ANS, ANR and LAR), modifying enzymes (*e.g.*, UFGT) and transcription (co-) factors (MYB, bHLH and WDR) obtained using BacHBerryEXP [69] is shown in Fig. 3 and transcript expression levels are presented in Additional file 15: Table S9. In some cases, gene transcript profiles exhibited a consensus trend, although for many genes, transcript-to-transcript profile variations were observed allowing us to group and select homologues according to their expression patterns. Expression of genes encoding enzymes involved in the phenylpropanoid pathway during fruit development has already been reported in *Rubus* sp. var. Lochness [75]. Transcription of genes encoding different isoforms of PAL either increased strongly from green to intermediate ripe fruits (RgPAL-1, RiPAL-1/- 2) or declined throughout fruit ripening stages (RgPAL-2) (Fig. 3b); presumably the isoforms induced during ripening are most closely associated with anthocyanin accumulation. Transcription of CHS homologues was mainly upregulated from green to the intermediate fruit stages (Fig. 3b). Transcripts encoding RgCHI-1 and RiCHI were upregulated until the intermediate ripening stage and then downregulated in ripe fruit, whereas RgCHI-2 was downregulated during fruit maturation (Fig. 3b) suggesting that it may not make a major contribution to anthocyanin biosynthesis. Coinciding with pigment accumulation in fruits, RgF3H-1 and RiF3H-1 were upregulated from green to immature fruits, with the highest levels at the ripe fruit stage (Fig. 3b) as previously observed for F3H in blackberry [75], while the transcript expression of RgF3H-2 and RiF3H-2 peaked at the intermediate ripe fruit stage and declined in ripe fruit (Fig. 3b and Additional file 15: Table S9). FLS homologues, RgFLS-1 and RiFLS, showed increased transcript levels from green to ripe fruits, whereas other FNS/FLS transcript levels, such as RgFNS and RiFNS-1, declined during fruit ripening confirming previous observations [75] or remained mainly unchanged (RiFNS-2 and RgFLS-2) during fruit ripening (Fig. 3b). The RiDFR-1 orthologue showed upregulation from green to intermediate ripe fruits, while transcript levels in the wild *Rubus* species (RgDFR) peaked at the immature ripening stage and declined in ripe fruits (Fig. 3b). Other DFR transcript isoforms (*e.g.*, RiDFR-2, Fig. 3b) exhibited steady downregulation from green to ripe fruits. RiANS-1 and RgANS-1 encode candidate ANS isoforms involved in the synthesis of coloured anthocyanidins and were strongly expressed up to the immature fruit stage, whereas other ANS homologues (*e.g.*, RgANS-2 and RiANS-2) were not induced during ripening (Fig. 3b).

Like other genes induced during ripening, transcripts encoding flavonol 3-O-glucosyltransferase (UFGT) in both *Rubus* species (Ri/RgUFGT, Fig. 3b) showed strong induction of transcript levels during ripening in line with the function of UFGT in stabilizing anthocyanidins by glucosylating them on the hydroxyl group of carbon 3, prior to transport to the vacuoles of the cells in coloured ripe fruits. Transcript levels for the PA biosynthesis gene *LAR* increased from green to the intermediate ripe fruits (RiLAR-1) or expression decreased steadily during fruit development (RgLAR-1, RgLAR-2, RiLAR-2 and RiLAR-3, Fig. 3b). For the PA-biosynthetic enzyme ANR, transcript levels decreased (RgANR) similarly to RuANR2 during fruit development [75] or expression levels remained similar during fruit development (RiANR) (Fig. 3b). Overall, it appeared that many flavonoid genes showed a coordinated expression pattern from the early stage to the production of anthocyanins in later stages of fruit development.

During fruit ripening, transport of anthocyanins and PAs is mediated mainly by two families of transporters, the ATP-binding cassette (ABC) transporter family and Multidrug and Toxic Compound Extrusion (MATE) transporters as well as glutathione S-transferases. Transcription of putative *Rubus* anthocyanin ABC transporters varied widely during fruit development (Fig. 3c and Additional file 15: Table S9). Candidates for anthocyanin and PA MATE homologues (*e.g.*, RgMATE-1 and RiMATE-1) were found either to be downregulated in ripening fruits or transcript levels peaked in intermediate ripe fruits (*e.g.*, RgMATE-2, RgMATE-3, RiMATE-2, RiMATE-3) (Fig. 3c). In blueberry, genes involved in vacuolar localization of PA exhibited developmental stage-specific expression patterns such as *ANS*, *UFGT*, *LAR* and *ANR* [2].

The transport of metabolites through endomembranes like the vacuolar tonoplast can be energized through proton pumps generated by P-ATPases such as PH5 whose gene expression is controlled directly by (pro)anthocyanin MBW complexes [96]. PH5 can act alone or in combination with another P-ATPase, PH1 that is absent in many plant species and boosts the activity of PH5 [97]. In *R. genevieri* and *R. idaeus*, some PH5 and PH1 homologues were downregulated (RgPH5-1, RiPH5-1), some upregulated (RgPH5-2) or their expression peaked at the immature fruit stage (RgPH5-3, RiPH5-2, RgPH1 and RiPH1) (Fig. 3c and Additional file 15: Table S9) suggesting roles in fruit hyperacidification like in pigmented grapevine where the expression of VvPH5 and VvPH1 peaked when berries changed colour at véraison [97].

### Expression of genes encoding regulators of flavonoid biosynthesis during fruit development in *Rubus* species

Expression of the regulatory genes such as *RiMyb10* and *RgMyb10* was strongly upregulated during fruit ripening, especially from unripe to intermediate ripe fruits, whereas *RiMyb12*, *RgMyb12*, *RiAn1* and *RiDel* were not differentially expressed at different stages of fruit ripening (Fig. 3d and Additional file 15: Table S9). A positive correlation between MYB10 transcript levels, anthocyanin synthesis and fruit colouration has also been reported in apple [59], blackberry [75], wild and cultivated strawberry as well as in sweet cherry [72]. RgAN1 was slightly upregulated from green to immature red fruits and moderately downregulated in ripe black fruits (Fig. 3d). In contrast, the expression of RubHLH1, a Ri/RgAN1 homologue, was consistently low in all stages of blackberry fruit ripening [75]. Interestingly, RgDEL expression was downregulated from green to ripe fruits (Fig. 3d), indicating that it is unlikely to be directly involved in anthocyanin formation in wild blackberry fruits but might activate the expression of RgAN1 as a part of the MBW complex involved in anthocyanin biosynthesis [60, 81] as also suggested by our expression assays where transient co-expression of RgDEL with RiMYB10 or RgMYB10 did not lead to leaf pigmentation in *N. benthamiana* while RiDEL and Ri/RgMYB10 did (Additional file 13: Figure S5). The transcript levels of Ri/RgTTG1 did not change during fruit development (Fig. 3d), whereas [75] reported that RuTTG1 showed slightly higher expression in green blackberries compared to later fruit ripening stages. Overall, Rg/RiDEL (tpm ≤ 1–2) and Rg/RiMYB12 (tpm ≤ 1) were both expressed at very low levels in fruit, whereas Ri/RgAN1 (up to tpm = 30–40), Rg/RiTTG1 (up to tpm = 10–20) and Rg/RiMYB10 (up to tpm = 35–120) were expressed at substantially higher levels in ripening fruits (Additional file 15: Table S9). This suggests that the highly abundant PhAN1-type bHLH TFs (RgAN1/RiAN1) are the dominant players and partners of the MYB10 TFs regulating anthocyanin production and fruit colouration during berry fruit ripening. The ability of RiDEL to activate anthocyanin biosynthesis in combination with Rg/RiMYB10 compared to the inability of RgDEL from the same genus to activate anthocyanin biosynthesis is noteworthy and suggests that the bHLH partners in the MBW complex may play slightly different roles even between different species in the same genus.

The expression of RgMYB6 (Fig. 3d), the closest homologue of the activators of flavonol biosynthesis in *G. max* (GmMYB12B2) and blackberry (RuMYB6), increased in ripe fruits like RuMYB6 [75]. On the other hand, RiMYB6 decreased with fruit ripening (Fig. 3d). For the RuMYB5 homologues, RiMYB5 and RgMYB5, expression peaked at the intermediate ripe fruit stage (Fig. 3d) which may relate to increases in PA synthesis in developing fruits in both species. This was confirmed by the identification of the PAs catechin and epicatechin in intermediate to ripe fruits (approximately 1/10 of the anthocyanin content) [51]. Fluctuations in MYB5 homologue expression have been reported previously. RuMYB5 from cultivated blackberry, has been predicted to interact with RuTTG1 and RubHLH1 in PA synthesis, and showed decreasing transcript levels during ripening [75]. In strawberry, FaMYB5 transcripts accumulate steadily during fruit development. It has been suggested that FaMYB5 may play a role in fine-tuning both PA biosynthesis during early fruit development and anthocyanin biosynthesis during fruit ripening [98]. Variations in expression patterns could also relate to the fact that another MYB5 homologue, AtMYB5 has been considered to be a general flavonoid pathway activator [73].

Homologues of RuMYB2, a putative PA synthesis activator by analogy to AtTT2, were strongly downregulated (RgMYB2) similarly to RuMYB2 or almost exclusively expressed in green fruits (RiMYB2) (Fig. 3d).

Transcript levels of *RgMyb1* and *RiMyb1*, encoding homologues of FaMYB1 (a transcriptional repressor of anthocyanin/PA biosynthesis in strawberry) were upregulated from green to intermediate ripe fruits and were transcribed abundantly in *R. genevieri* or at low level in *R. idaeus* (Fig. 3d). The AtMYB4 phenylpropanoid repressor homologues in both *Rubus* species (RgMYB4 and RiMYB4, Fig. 3d) were downregulated during ripening as was RuMYB4 in blackberry [75].

## Conclusions

We report transcriptome sequences and analytical tools for gene identification, cloning and functional analysis from 13 berry fruit species coming from Europe, South America and Asia, spanning eight plant genera and seven families. Tools and resources are accessible and searchable online *via* the BacHBerryGEN database [67, 68] and the BacHBerryEXP gene expression browser [69]. These resources will assist gene expression and functional genomic studies in berry fruit species as well as contributing to the understanding of the synthesis of polyphenols, the molecular mechanisms underlying phenylpropanoid, and particularly flavonoid, synthesis and the regulatory processes controlling phenylpropanoid metabolism during fruit ripening. These tools have already contributed to identifying the genes involved in the synthesis of novel biologically active compounds in berry fruits [26]. Ultimately, studies of metabolic pathways should facilitate breeding programmes for fleshy fruits by providing markers for shortening the long process of breeding and by identifying valuable and better varieties, resulting in benefits to both consumers and farmers.

The usefulness of these transcriptomic resources has been demonstrated by the cloning and characterisation of regulators of the anthocyanin pathway from these berry fruit species, namely R2R3-type MYBs, bHLH and WDR homologues, which regulate anthocyanin and PA biosynthesis in two *Rubus* species. Functional validation of *Rubus* homologues of MdMYB10, AtMYB12, PhAN1/AmDEL and AtTTG1/MdTTG1 was undertaken in *N. benthamiana* leaves. The regulators Rg/RiMYB10, Rg/RiAN1, RiDEL and Ri/RgTTG1, are likely to be part of red raspberry (*R. idaeus*) and wild blackberry (*R. genevieri*) MBW complexes, respectively regulating the expression of flavonoid genes late in anthocyanin biosynthesis. However, the DEL homologue from wild blackberry was unable to induce pigment formation with Ri/RgMYB10 perhaps as a result of the few aa differences found between RgDEL and RiDEL.

There is a growing interest in the exploitation of wild fruits and berries as part of the rising demand for novel health promoting foods. In berries, high antioxidant activity is most often associated with berries from wild species, together with a broad variety of anthocyanins and high total polyphenol contents compared to cultivated varieties. In commercial cultivars, the flavonoid content has often been altered and reduced during domestication with an accompanying increase in susceptibility towards pests/insects [99]. In this study, we investigated a wide range of wild and cultivated berry fruit species representing diverse plant families (Berberidaceae, Caprifoliaceae, Elaeocarpaceae, Ericaceae, Grossulariaceae, Rosaceae and Myrtaceae) to provide a broad platform for classification of genes and their products and to establish fruit-specific expression patterns to gain new insights into the complex regulation of metabolic pathways during berry fruit development.

The demand for varied, nutritious and healthy food has been growing in both the developed and developing world. Diets that include berries and fruits are rich in polyphenols including monolignols, flavonoids (anthocyanins, PAs, flavonols, flavones, flavanones, isoflavonoids and phlobaphenes), various phenolic acids and stilbenes. All these polyphenols have been shown to play important roles in plant growth and development, biotic and abiotic defence mechanisms as well as conferring benefits for human health.

## Methods

### Plant materials and isolation of total RNA

Plant tissues from 13 berry fruit species were collected by partners of the BacHBerry Consortium [26] in Chile, China, United Kingdom, Portugal and Russia (Table 1 and Additional file 1: Table S1). Plants were grown either in their natural habitat or under cultivated conditions (Additional file 1: Table S1). Fruits were harvested at different developmental ripening stages (unripe, intermediate and/or ripe fruits) and leaf material was collected from fully developed leaves between January to August 2014 and July to August 2015 (Additional file 1: Table S1).

RNA was extracted from 13 berry fruit species using *(i)* ripe fruits of ten species, *i.e.*, *A. chilensis*, *B. buxifolia*, *L. caerulea*, *R. nigrum* cv. Ben Hope, *R. nigrum* var. *sibiricum* cv. Biryusinka (also described as *R. nigrum* subsp. *sibiricum* cv. Biryusinka), *R. idaeus* cv. Octavia, *R. vagabundus*, *U. molinae*, *V. corymbosum* and *V. uliginosum*; *(ii)* unripe (green), intermediate (pale red) and ripe (dark red) fruits of *R. idaeus* cv. Prestige; *(iii)* unripe (green), intermediate (red) and ripe (black) fruits of *R. genevieri* and *(iv)* leaf material of *C. album* (Table 1 and Additional file 1: Table S1). Plant material was frozen in liquid nitrogen immediately after harvest, stored at -80 °C and transported in dry ice prior to RNA extraction. Leaves as well as deseeded whole berries and fruits were ground to a fine powder with liquid nitrogen. Total RNA of *A. chilensis*, *B. buxifolia*, *R. idaeus* cv. Octavia, *R. vagabundus, V. corymbosum*, *V. uliginosum* and *U. molinae* was isolated from 200 mg of frozen fruit tissue based on a protocol for plant tissues rich in polyphenols and polysaccharides [100] and included an additional DNase I treatment (RQ1 RNase-Free DNase, Promega) before phenol: chloroform extraction within step III of the protocol. For *C. album*, *L. caerulea*, *R. nigrum* cv. Ben Hope*, R. nigrum* var*. sibiricum* cv. Biryusinka, *R. genevieri* and *R. idaeus* cv. Prestige, total RNA was extracted from 200 mg of frozen fruit tissue and leaves, respectively by using the Spectrum Plant Total RNA kit (Sigma) following the manufacturer’s guidelines and protocol A (using 750 μl binding solution). The optional step of on-column DNase digestion was performed and total RNA was eluted with 60 μl elution buffer. For each sample, the total RNA of 10–15 fruits was pooled. The RNA concentration and quality were evaluated *via* spectrophotometric analysis and the RNA integrity was also analysed by gel electrophoresis. Overall, 17 total RNA samples were produced for the 13 species: ten species (ripe fruit), two species (three fruit ripening stages) and one species (leaf).

### Synthesis of cDNA and RNA sequencing

cDNA library preparation and sequencing were carried out by the Earlham Institute, formerly The Genome Analysis Centre (TGAC) Norwich, UK. The libraries were constructed on the Sciclone NGS Workstation (PerkinElmer) following the Illumina TruSeq RNA sample preparation v2 guide and using the TruSeq RNA Library Preparation Kit v2 (Illumina). The library preparation involved several quality control analysis steps, including the use of the Quant-iT™ RNA Assay Kit (Life Technologies) for RNA quantification, the Quant-iT™ dsDNA Assay Kit (Life Technologies) for double-stranded DNA quantification as well as the LabChip GX Automated Electrophoresis System (PerkinElmer) and High Sensitivity DNA kit (Agilent) for RNA/dsDNA quantification and verification of the cDNA library insert size. Shortly, the RNA-seq workflow included *(1)* purification and fragmentation of mRNA from 1 μg of total RNA with a poly(A)-pull down using oligo-dT attached magnetic beads; (*2*) first strand cDNA synthesis with random hexamer primers and SuperScript II reverse transcriptase (Invitrogen); *(3)* second strand cDNA synthesis using DNA polymerase I and RNase H; *(4)* cDNA end repair/blunting; *(5)* cDNA fragment 3′ end adenylation; *(6)* ligation of multiple indexing adapters to cDNA fragments and purification of ligated products *via* bead-based size selection using AMPure XP beads (Beckman Coulter); *(7)* PCR enrichment of adapter-ligated cDNA fragments with a PCR primer cocktail that anneals to the adapter ends; *(8)* quantitative and qualitative validation of cDNA library; *(9)* normalisation and equimolar pooling of indexed DNA libraries; *(10)* dilution of library pool to a final concentration of 10 pM and spiking of each library pool with 1% PhiX Control v3 (Illumina); *(11)* flow cell clustering using the TruSeq PE Cluster Kit v3-cBot-HS (Illumina); and *(12)* sequencing of flow cell using the Illumina HiSeq™ 2000 platform with the TruSeq SBS Kit v3-HS (Illumina) and HiSeq Control Software 2.2.58 and RTA 1.18.64. Reads in bcl format were de-multiplexed based on the 6 bp Illumina index by the CASAVA 1.8 package allowing for a one base-pair mismatch per library and converted to FASTQ format by bcl2fastq.

### De novo assembly of transcriptomes

Illumina data from total RNA samples of the 13 berry fruit species were assembled by the Earlham Institute (Norwich, UK) into individual de novo transcriptomes using Trinity [101] and these assemblies were then used as a reference for mapping, quantification of expression and functional annotation. The alignment of RNA-seq reads to a transcriptome reference was performed using TopHat2 [102] with a minimum anchor length of 12 and a maximum of 20 multihits. Adapter/primer sequences were clipped, and low-quality reads were removed. Quality control of the raw data was performed using FastQC [103] and the contamination screening and filtering tool Kontaminant [104]. Gene/isoform expression was quantified using Cufflinks [105]. Transdecoder [106] was used to extract ORFs from the de novo transcriptome assemblies. Peptides of these ORFs were annotated *via* an in-house pipeline (AnnotF [107]) that compares the results of Blast2GO and InterProSCAN (Additional file 6: Table S5). Transcriptome sequences of *R. genevieri* and *R. idaeus* cv. Prestige fruits at three different stages of maturation (with three biological replicates per ripening stage) were either kept separate to undertake subsequent differential expression studies or pooled together to generate a consensus fruit sequence.

### Mining of fruit transcriptomes, BLAST searches, expression profiling and phylogenetic analysis

The BacHBerryGEN database [67] was created to deposit the transcriptomic data of the 13 berry fruit species. A BLAST search engine [68] was developed to conduct homology-based searches of candidate genes. The workflow MassBlast [70, 108] was also used to identify homologues and orthologues of enzymes responsible for the synthesis, decoration and regulation of phenylpropanoid compounds.

The BacHBerryEXP expression browser [69] was established based on [93] to facilitate the differential expression analysis of candidate genes of two *Rubus* species, *R. genevieri* and *R. idaeus* cv. Prestige, during three developmental stages of fruit ripening (green/immature/ripe). Six transcriptome sequences of *R. genevieri* and *R. idaeus* cv. Prestige (two species x three fruit ripening stages) were uploaded in the BacHBerryEXP expression browser [69] and used to analyse differential transcription of genes involved in the anthocyanin biosynthetic pathway. The transcript identification ID can be retrieved either in the BacHBerryGEN database [68] or by using the built-in BLAST search module of the BacHBerryEXP expression browser [69].

The phylogenetic analysis of the transcriptomes of the 13 berry fruit species together with the genome sequences of seven reference species (*A. thaliana* [109], *P. trichocarpa* [110], *G. max* [111], *V. vinifera* [5], *S. lycopersicum* [112], *O. sativa* [113] and *A. trichopoda* [114]) was carried out following the comparative genomic analysis detailed in [115]. EvidentialGene [116] was used to translate and validate the ORFs. The longest translated ORFs from each gene with at least 100 aa were aligned with BLAST 2.26 [117] against each other and orthologue groups were identified using OrthoMCL 2.0.9 [118]. A multiple alignment for each of the 214 single-copy gene families was produced using MUSCLE v3.8.1551 [119]. The longest contiguous block of each aa sequence, defined as more than 20 aa with less than five contiguous positions with a gap in any sequence, were concatenated to produce a super alignment matrix containing 205 gene families (with nine gene families filtered out due to the lack of a single contiguous block) using a BioRuby script [120] (Additional file 16). ProtTest 3.4.2 [121] was used to find the best fitting model to produce the phylogenetic tree which was JTT + I + G + F. The phylogenetic tree was assembled using RAxML 8.2.12 [122] with the option PROTGAMMAJTT and declaring *O. sativa* and *A. trichopoda* as outgroups. The divergence time among the 20 species was estimated using MCMCtree 4.8a from the PAML software [123] (Additional file 16). The species divergence time was predicted using the calibration points of *(i) A. thaliana* and *P. trichocarpa* (107–109 million years (My) ago), *(ii) A. thaliana* and *G. max* (107–109 My ago), *(iii) S. lycopersicum* and *P. trichocarpa* (107–125 My ago), *(iv) O. sativa* and *A. thaliana* (140–200 My ago) and *(v) V. vinifera* and *A. thaliana* (113–114 My ago) [124]. The Additional file 16 details the programme execution.

### Identification and cloning of regulatory genes from *R. genevieri* and *R. idaeus* cv. Prestige

Orthologues of *(i)* R2R3-type MYB transcription factors (TFs): MdMYB10 (*M. domestica* cv. Maypole MYB10, Accession no. AB744002.1) and AtMYB12 (*A. thaliana* MYB domain protein 12, Accession no. NM_130314.4) TFs; *(ii)* bHLH TFs: PhAN1 (*P. hybrida* ANTHOCYANIN 1, Accession no. AF260919) and AmDEL (*A. majus* DELILA, Accession no. M84913.1) TFs and *(iii)* a WD40-repeat gene: MdTTG1 (*M. domestica* TRANSPARENT TESTA GLABRA1; Accession no. GU173814.1) were identified in *R. genevieri* and *R. idaeus* cv. Prestige by mining the fruit transcriptomic data deposited in the BacHBerryGEN database [67] using the TBLASTN programme of the BacHBerryGEN BLAST server [68] and a protein sequence query (Additional file 7: Table S6).

Total RNA (1 μg) extracted from ripe fruits of both *Rubus* species was used for first strand cDNA synthesis with oligo (dT)_18_ primers (Sigma) using SuperScript III reverse transcriptase (Invitrogen) and RNaseOUT (Recombinant Ribonuclease Inhibitor, Invitrogen) following the manufacturer’s instructions. First strand cDNAs were amplified using primers specific to the 5′ and 3′ ends of each gene (Additional file 8: Table S7) and PfuUltra® II Fusion HS DNA Polymerase (Agilent Genomics). The PCR amplification was carried out with an initial denaturation of 2 min at 94 °C followed by 40 cycles of 94 °C for 30 s, 60 °C for 30 s and 72 °C for 1 min (for MdMYB10 homologues), for 1.5 min (for AtMYB12 and MdTTG1 homologues) or for 2 min (for bHLH homologues) and a final elongation of 3 min at 72 °C.

To facilitate the cloning of the various TF RT-PCR products, the CaMV 35S promoter (pro) and soybean poly(A) (SPA) terminator (ter) sequences [125] were cloned into three basic vectors *(i)* pGreenII0029 (containing a NOS-pro::*nptII*::NOS-ter plant selectable marker gene) [126], *(ii)* pGreenII00179 (harbouring a CaMV35S-pro::*hpt*::CaMV35S-ter plant selectable marker gene) [126] and *(iii)* pGreenII00229 (possessing a NOS-pro::*bar*::NOS-ter plant selectable marker gene) [126]. The RT-PCR fragments of the *Rubus* regulatory genes were inserted between the CaMV 35S-pro and SPA-ter sequences as blunt-end fragments or *Bam*HI/*Xba*I-*Pst*I/*Sma*I/*Xho*I/*Nsi*I-digested fragments (Additional file 7: Table S6 and Additional file 8: Table S7). The TF RT-PCR fragments and vector combinations are indicated in Additional file 7: Table S6.

### *Agrobacterium*-mediated transient expression and stable transformation of regulatory genes in *N. benthamiana*

The different pGreen-based vectors were introduced together with pSoup [126] or a pSoup derivative containing the viral suppressor of gene silencing P38 from Turnip Crinkle Virus (pBOOST-S; CaMV35S-pro::P38::SPA-ter cassette in vector pCLEAN-S161 [125]) into the *Agrobacterium tumefaciens* strain AGL1 *via* a freeze-thaw method [127]. The presence of the different regulatory genes in the *Agrobacterium* strains was confirmed by PCR amplification of the full-length genes from *Agrobacterium* plasmid preps and sequencing of the PCR products. Transient assays in *N. benthamiana* were carried out as described in [125] in two accessions of *N. benthamiana*, in the Australian ecotype Northern Territory (NT; seeds kindly provided by Prof. Peter Waterhouse, Queensland University of Technology, Brisbane, Australia) [80] and the John Innes Centre laboratory isolate (JIC-LAB; predicted to be of the same origin as the LAB isolate of [80]). For co-infiltration assays using *Agrobacterium* strains harbouring *Myb*, *bHLH* or *WDR* homologues of *R. genevieri* and *R. idaeus* cv. Prestige, *Agrobacterium* strains (OD_600_ = 1.0) were mixed equally. A so-called empty vector strain without a gene of interest (pGreenII00179 + pBOOST-S in AGL1) was used as a negative control or as a component of the co-infiltration mixes to complement for co-factors (*e.g.*, WDR). Four- to five-week old *N. benthamiana* plants were used for patch infiltration to monitor the production of polyphenolic compounds (mainly anthocyanins and flavonols). The abaxial side of three to four leaves per plant were infiltrated, the leaves were observed from 1 to 14 days post infiltration (dpi) and samples were harvested from 1 to 7 dpi.

Regulatory genes were stably transformed into *N. benthamiana* (JIC-LAB isolate) using either leaf or stem explants following the *Agrobacterium*-mediated transformation protocol of *Moricandia arvensis* [128]. Single selection based on kanamycin (100 mg/l) was used for the transformation with *Myb* genes. Dual selection based on kanamycin (30 mg/l) and hygromycin (10 mg/l) was applied for the co-transformation with *Myb* and *bHLH* genes. Triple selection based on kanamycin (30 mg/l), hygromycin (10 mg/l) and DL-phosphinothricin (PPT, 3 mg/l) was used for the co-transformation with *Myb*, *bHLH* and *WDR* genes.

### Detection and analysis of polyphenolic compounds

Leaf discs were cut from patch-infiltrated *N. benthamiana* (JIC-LAB isolate and NT accession) leaves using a standard cork borer (diameter of 1.8 cm). The top two to three infiltrated leaves were sampled avoiding main veins. In time course experiments eight leaf discs were collected from two different plants (*i.e.*, four to six infiltrated leaves from two plants) from 1 dpi to up to 7 dpi per time point. Each infiltration mix was tested several times in both *N. benthamiana* isolates. The leaf discs with a fresh weight of 36.89 ± 0.92 mg (JIC-LAB isolate) or 51.29 ± 1.47 mg (NT) were cut into quarters and immediately merged into 2 ml extraction solution (methanol: water: HCl, 80:20:1, v/v/v), quickly vortexed and incubated with gentle shaking overnight (16 h) at 4 °C in the dark. Following 3 h of moderate rocking at room temperature, extracts were centrifuged at 13,000 rpm for 15 min and the supernatants were analysed using a spectrophotometer at A_530nm_ for their anthocyanin content. The absorption at 530 nm was averaged for the eight leaf discs (avgA_530_) and the standard error was calculated for each treatment and time point.

Leaf disc samples from *Agrobacterium*-infiltrated leaf patches of *N. benthamiana* (JIC-LAB isolate) were taken 5 dpi and analysed by HPLC/photodiode array detector (PDA) and mass spectrometry (MS) in comparison to chlorogenic acid and rutin standards (serial dilutions in 20% methanol) using the Shimadzu IT-ToF and a Kinete× 2.6 μm EVOC18, 100 Å pore size LC column (100-× 2.1-mm) according to [90]. Polyphenolic compounds (mainly anthocyanins and flavonols) were identified based on their mass and mass of their fragments, respectively.

## Supplementary information

**Additional file 1: Table S1.** Classification and description of berry fruit species.

**Additional file 2: Figure S1.** Schematic representation of the phylogenetic relationship among the 13 berry fruit species studied.

**Additional file 3: Table S2.** RNA-seq descriptors for 13 berry fruit species.

**Additional file 4: Table S3.** RNA-seq descriptors for two *Rubus* species at three fruit ripening stages: green, immature and ripe.

**Additional file 5: Table S4.** BLAST search output summaries for transcript candidates involved in the phenylpropanoid pathway for 13 berry fruit species: Enzymes of the core pathway and its decorating, modifying and regulatory proteins.

**Additional file 6: Table S5.** Peptide annotation for the transcriptomes of *R. genevieri* and *R. idaeus* cv. Prestige.

**Additional file 7: Table S6.** Identification and cloning of regulatory genes of the phenylpropanoid pathway from *R. genevieri* (A) and *R. idaeus* cv. Prestige (B).

**Additional file 8: Table S7.** Primers used for the cloning of regulatory genes of the phenylpropanoid pathway from *R. genevieri* (A) and *R. idaeus* cv. Prestige (B).

**Additional file 9: Figure S2.** Phylogenetic relationship and protein sequence alignment of a subset of R2R3-type MYB transcription factor homologues.

**Additional file 10: Figure S3.** Phylogenetic relationship and protein sequence alignment of a subset of bHLH transcription factor homologues.

**Additional file 11: Figure S4.** Phylogenetic relationship, prediction of WD40 motifs and protein sequence alignment of a subset of WDR homologues.

**Additional file 12: Table S8.** Homologues of the regulatory genes cloned in this study in the collection of the 13 berry fruit species.

**Additional file 13: Figure S5.** Production of anthocyanins in leaves of two accessions of *N. benthamiana*, JIC-LAB strain and cv. NT, following transient overexpression of regulatory genes from *R. genevieri* and *R. idaeus* cv. Prestige at various time points after infiltration (4 dpi to 14 dpi) alone or in combination.

**Additional file 14: Figure S6.** Examples of anthocyanin formation in kanamycin, hygromycin and/or PPT-resistant *N. benthamiana* calli and shoots transformed with *Rubus Myb*, *bHLH* and *WDR* regulatory genes.

**Additional file 15: Table S9.** Differential expression of candidate transcripts homologous to enzymes involved in the phenylpropanoid pathway of *R. genevieri* and *R. idaeus* cv. Prestige during three fruit ripening stages: Examples of candidate enzymes of the general phenylpropanoid pathway, flavonoid regulatory enzymes, transporters, decorating and modifying enzymes.

**Additional file 16: **Step-by-step guide of the phylogenetic analysis of the transcriptomes of the 13 berry fruit species together with the reference genomes of *A. thaliana*, *P. trichocarpa*, *G. max*, *V. vinifera*, *S. lycopersicum*, *O. sativa* and *A. trichopoda*.

## Data Availability

The transcriptome sequences generated during this current study are available from the BacHBerryGEN repository (http://jicbio.nbi.ac.uk/berries). BLAST and expression analyses can be performed using the BacHBerryGEN database (http://jicbio.nbi.ac.uk/berries/blast.html) and BacHBerryEXP gene expression browser (http://www.bachberryexp.com), respectively. Nucleotide sequences of *Rubus* flavonoid regulatory genes cloned in this study are deposited in GenBank. Additional data generated and/or analysed during this study are included either in this published article as supplementary information or can be requested from the authors (e.g., transcriptome ORF/peptide annotations and Trinotate annotations of secondary metabolite biosynthesis /phenylpropanoid pathway for each transcriptome). Metabolomics datasets of the 13 berry fruit species studied in this manuscript are available at https://ics.hutton.ac.uk/germinate-berrybase/# (Dr. Alexandre Foito and Prof. Derek Stewart; The James Hutton Institute, Invergowrie, UK). Berry fruit material can be requested from the institutions listed in Table 1 and Additional file 1: Table S1.

## Abbreviations

4CL: 4-coumarate CoA ligase
aa: amino acid
ABC transporter: ATP-binding cassette transporter
Am: *Antirrhinum majus*
ANR: Anthocyanidin reductase
ANS: Anthocyanidin synthase
At: *Arabidopsis thaliana*
avg: average
*bar*: phosphinothricin acetyl transferase
bHLH: basic helix-loop-helix protein
C4H: Cinnamate 4-hydroxylase
CaMV35S: Cauliflower Mosaic Virus 35S
CHI: Chalcone isomerase
CHS: Chalcone synthase
DFR: Dihydroflavonol 4-reductase
dpi: days post infiltration
F3’5’H: Flavonoid 3’,5’-hydroxylase
F3’H: Flavonoid 3’-hydroxylase
F3H: Flavanone 3-hydroxylase
Fa: *Fragaria × ananassa*
FLS: Flavonol synthase
FNS: Flavone synthase
Gm: *Glycine max*
*hpt*: hygromycin phosphotransferase
LAR: Leucoanthocyanidin reductase
MATE transporter: Multidrug and Toxic Compound Extrusion transporter
MBW: MYB, bHLH and WDR protein complex
Md: *Malus x domestica*
My: Million years
MYB: MYB-domain containing protein
NOS: Nopaline synthase
*nptII*: neomycin phosphotransferase II
nt: nucleotide
ORF: Open reading frame
PA: Proanthocyanidin
PAL: Phenylalanine ammonia lyase
Ph: *Petunia x hybrida*
Rg: *Rubus genevieri*
Ri: *Rubus idaeus* cv. Prestige
Ru: *Rubus* sp. var. Lochness
SG: subgroup
Sl: *Solanum lycopersicum*
SPA: Soybean poly(A) signal
STS: Stilbene synthase
TF: Transcription factor
tpm: transcripts per million
UFGT: UDP glucose: flavonol 3-O-glucosyltransferase
Vv: *Vitis vinifera*
WDR: WD40 motif containing protein
