# Supplementary material for: RNA-seq, de novo transcriptome assembly and flavonoid gene analysis in 13 wild and cultivated berry fruit species with high content of phenolics
Source: BMC Genomics. 2019 Dec 19;20:995. doi: 10.1186/s12864-019-6183-2 (PMC6924045; doi:10.1186/s12864-019-6183-2)
Supplement: Supplementary file 2 — Additional file 2: Figure S1. Schematic representation of the phylogenetic relationship among the 13 berry fruit species studied. [file 12864_2019_6183_MOESM2_ESM.pdf]

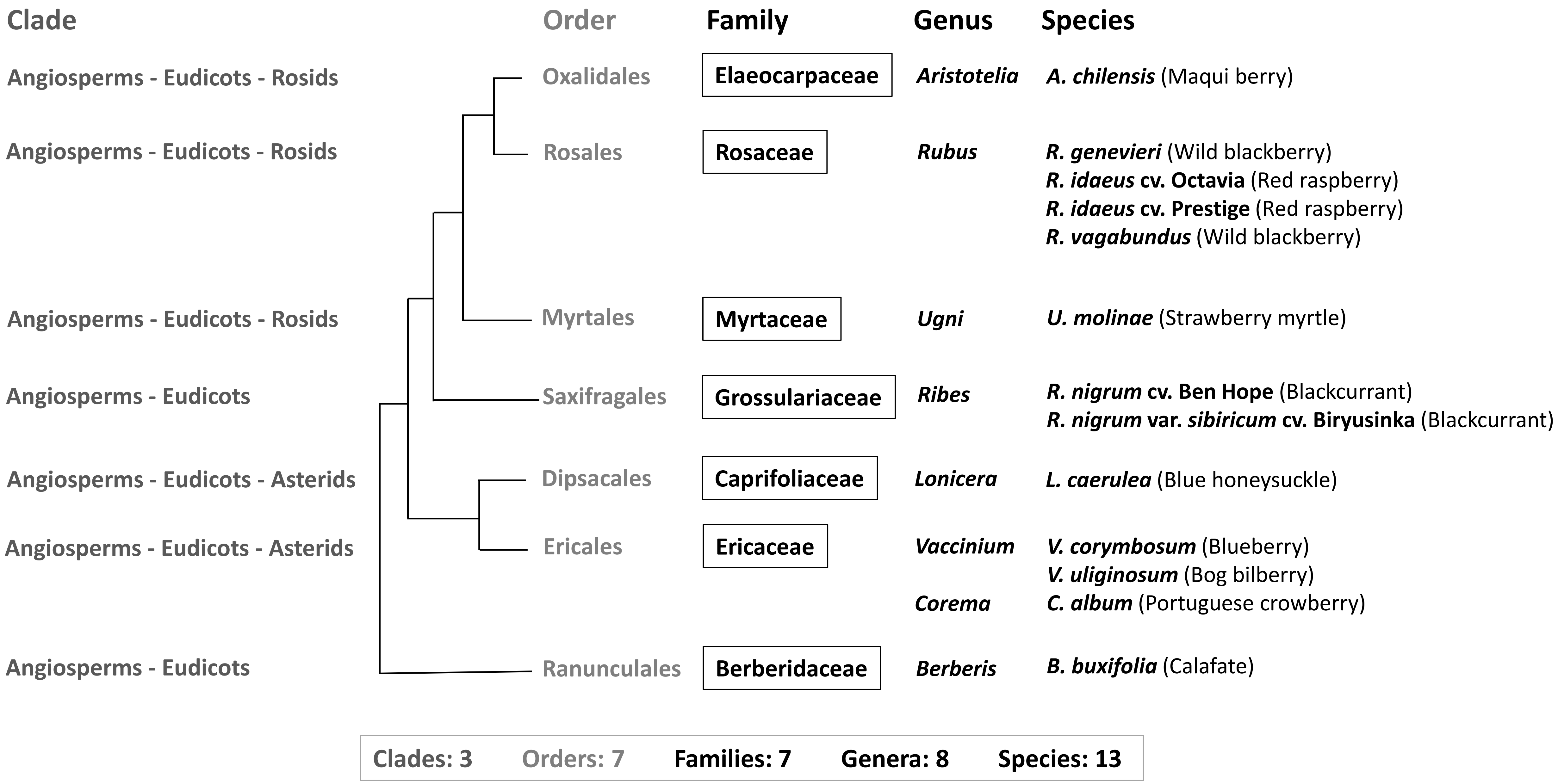

Additional file 2: Fig. S1. Schematic representation of the phylogenetic relationship among the 13 berry fruit species studied.
