# Supplementary material for: RNA-seq, de novo transcriptome assembly and flavonoid gene analysis in 13 wild and cultivated berry fruit species with high content of phenolics
Source: BMC Genomics. 2019 Dec 19;20:995. doi: 10.1186/s12864-019-6183-2 (PMC6924045; doi:10.1186/s12864-019-6183-2)
Supplement: Supplementary file 7 — Additional file 7: Table S6. Identification and cloning of regulatory genes of the phenylpropanoid pathway from R. genevieri (A) and R. idaeus cv. Prestige (B). [file 12864_2019_6183_MOESM7_ESM.docx]

**Additional file 7: Table S6.** Identification and cloning of regulatory genes of the phenylpropanoid pathway from *R. genevieri* (A) and *R. idaeus* cv. Prestige (B).

| Gene function | Orthologue used for gene  identification* | Transcript identified in fruit transcriptome* | Cloned gene  name | Cloned  gene (nt) /  protein (aa) | | Expression vector** | | GenBank Accession  No. | |
| --- | --- | --- | --- | --- | --- | --- | --- | --- | --- |
| (A) *R. genevieri* (Rg) | | | | | | | | |  |
| R2R3-type MYB TF (SG6) | *Malus x domestica* cv. Maypole MYB10  (AB744002.1)  (Umemura *et al.*, 2013) | [TR103098_c0_g1_i1](http://jicbio.nbi.ac.uk/cgi-bin/trinity_extract.pl?db=rubus_genevieri.fa&ref=TR103098_c0_g1_i1) | *RgMyb10* | 654 nt  217 aa | | pGreenII0029 derivative | | KY111315 | |
| R2R3-type MYB TF (SG7) | *Arabidopsis thaliana* MYB domain protein 12 (NM_130314.4)  (Mehrtens *et al.*, 2005) | [TR71550_c1_g1_i1](http://jicbio.nbi.ac.uk/cgi-bin/trinity_extract.pl?db=rubus_genevieri.fa&ref=TR71550_c1_g1_i1) | *RgMyb12* | 1296 nt  431 aa | | pGreenII0029 derivative | | KY111316 | |
| PhAN1-like bHLH TF  (SG IIIf-1) | *Petunia x hybrida* ANTHOCYANIN 1 (AN1)  (AF260919)  (Spelt *et al.*, 2000) | [TR110272_c1_g1_i1](http://jicbio.nbi.ac.uk/cgi-bin/trinity_extract.pl?db=rubus_genevieri.fa&ref=TR110272_c1_g1_i2) | *RgAn1-1* | 2100 nt  699 aa | | pGreenII00179 derivative | | KY123749 | |
|  |  | [TR110272_c1_g1_i1](http://jicbio.nbi.ac.uk/cgi-bin/trinity_extract.pl?db=rubus_genevieri.fa&ref=TR110272_c1_g1_i2) | *RgAn1-2* | 2103 nt  700 aa | | pGreenII00179 derivative | | KY123750 | |
|  |  | [TR110272_c1_g1_i1](http://jicbio.nbi.ac.uk/cgi-bin/trinity_extract.pl?db=rubus_genevieri.fa&ref=TR110272_c1_g1_i2) | *RgAn1-3* | 2100 nt  699 aa | | pGreenII00179 derivative | | KY123751 | |
| AmDEL-like bHLH TF  (SG IIIf-2) | *Antirrhinum majus* DELILA (DEL) (M84913.1)  (Goodrich *et al.*, 1992) | [TR110629_c1_g1_i1](http://jicbio.nbi.ac.uk/cgi-bin/trinity_extract.pl?db=rubus_genevieri.fa&ref=TR110629_c1_g1_i1) | *RgDel* | 1929 nt  642 aa | | pGreenII00179 derivative | | KY111317 | |
| WD40-repeat protein | *M. domestica* cv. Rewena TRANSPARENT TESTA GLABRA1 (TTG1) homologue  (GU173814.1) (Brueggemann *et al.*, 2010) | [TR29409_c0_g1_i1](http://jicbio.nbi.ac.uk/cgi-bin/trinity_extract.pl?db=rubus_genevieri.fa&ref=TR29409_c0_g1_i1) | *RgTTG1-1* | 1041 nt  346 aa | | pGreenII0229 & pGreenII00179 derivatives | | MH460860 | |
|  |  | [TR29409_c0_g1_i1](http://jicbio.nbi.ac.uk/cgi-bin/trinity_extract.pl?db=rubus_genevieri.fa&ref=TR29409_c0_g1_i1) | *RgTTG1-2* | 1041 nt  346 aa | | pGreenII0229 derivative | | MH460861 | |
| (B) *R. idaeus* cv. Prestige (Ri) | | | | | | | | |  |
| R2R3-type MYB TF (SG6) | *M. domestica* cv. Maypole MYB10  (AB744002.1)  (Umemura *et al.*, 2013) | [TR49283_c2_g2_i2](http://jicbio.nbi.ac.uk/cgi-bin/trinity_extract.pl?db=LIB19419-LIB19427_Trinity_NR.fasta&ref=TR49283_c2_g2_i2) | *RiMyb10* | 654 nt  217 aa | | pGreenII0029 derivative | | KY111313 | |
| R2R3-type MYB TF (SG7) | *A. thaliana* MYB domain protein 12  (NM_130314.4) (Mehrtens *et al.*, 2005) | [TR1036_c0_g1_i2](http://jicbio.nbi.ac.uk/cgi-bin/trinity_extract.pl?db=LIB19419-LIB19427_Trinity_NR.fasta&ref=TR1036_c0_g1_i2) | *RiMyb12* | 1272 nt  423 aa | | pGreenII0029 derivative | | KY111314 | |
| PhAN1-like bHLH TF  (SG IIIf-1) | *P. hybrida* ANTHOCYANIN 1 (AN1)  (AF260919)  (Spelt *et al.*, 2000) | [TR75681_c0_g1_i1](http://jicbio.nbi.ac.uk/cgi-bin/trinity_extract.pl?db=LIB19419-LIB19427_Trinity_NR.fasta&ref=TR75681_c0_g1_i1) | *RiAn1* | 2100 nt  699 aa | | pGreenII00179 derivative | | KY111320 | |
| AmDEL-like bHLH TF  (SG IIIf-2) | *A. majus* DELILA (DEL) (M84913.1)  (Goodrich *et al.*, 1992) | [TR16024_c0_g1_i1](http://jicbio.nbi.ac.uk/cgi-bin/trinity_extract.pl?db=LIB19419-LIB19427_Trinity_NR.fasta&ref=TR16024_c0_g1_i1) | *RiDel-1* | 1926 nt  641 aa | | pGreenII0029 derivative | | KY111318 | |
|  |  | [TR16024_c0_g1_i1](http://jicbio.nbi.ac.uk/cgi-bin/trinity_extract.pl?db=LIB19419-LIB19427_Trinity_NR.fasta&ref=TR16024_c0_g1_i1) | *RiDel-2* | 1929 nt  642 aa | | pGreenII00179 derivative | | KY111319 | |
| WD40-repeat protein | *M. domestica* cv. Rewena TRANSPARENT TESTA GLABRA1 (TTG1) homologue  (GU173814.1)  (Brueggemann *et al.*, 2010) | [TR7065_c0_g2_i1](http://jicbio.nbi.ac.uk/cgi-bin/trinity_extract.pl?db=LIB19419-LIB19427_Trinity_NR.fasta&ref=TR7065_c0_g2_i1) | *RiTTG1* | 1035 nt  344 aa | pGreenII0229 & pGreenII00179 derivatives | | MH460862 | |  |

* BLAST searches were undertaken using the BacHBerryGEN BLAST portal (<http://jicbio.nbi.ac.uk/berries/blast.html>).

** The original vectors pGreenII0029, pGreenII00179 and pGreenII0229 are described in Hellens *et al.*, 2000 *Plant Mol. Biol.*42:819-832. Their derivatives contain a CaMV 35S-pro and SPA-ter cassette (described in Thole *et al.*, 2007 *Plant Physiol.* 145-1211-1219) and were cloned in this study.

**References:**

Brueggemann J, Weisshaar B, Sagasser M. A WD40-repeat gene from *Malus x domestica* is a functional homologue of *Arabidopsis thaliana* TRANSPARENT TESTA GLABRA1. Plant Cell Rep. 2010; 29(3):285-294.

Goodrich J, Carpenter R, Coen ES. A common gene regulates pigmentation pattern in diverse plant species. Cell. 1992; 68(5):955-964.

Mehrtens F, Kranz H, Bednarek P, Weisshaar B. The *Arabidopsis* transcription factor MYB12 is a flavonol-specific regulator of phenylpropanoid biosynthesis. Plant Physiol. 2005; 138:1083-1096.

Spelt C, Quattrocchio F, Mol JNM, Koes R. Anthocyanin1 of petunia encodes a basic Helix-Loop-Helix protein that directly activates transcription of structural anthocyanin genes. Plant Cell 2000; 12:1619-1631.

Umemura H, Otagaki S, Wada M, Kondo S, Matsumoto S. Expression and functional analysis of a novel MYB gene, MdMYB110a_JP, responsible for red flesh, not skin color in apple fruit. Planta 2013; 238(1):65-76.
