# Supplementary material for: RNA-seq, de novo transcriptome assembly and flavonoid gene analysis in 13 wild and cultivated berry fruit species with high content of phenolics
Source: BMC Genomics. 2019 Dec 19;20:995. doi: 10.1186/s12864-019-6183-2 (PMC6924045; doi:10.1186/s12864-019-6183-2)
Supplement: Supplementary file 8 — Additional file 8: Table S7. Primers used for the cloning of regulatory genes of the phenylpropanoid pathway from R. genevieri (A) and R. idaeus cv. Prestige (B). [file 12864_2019_6183_MOESM8_ESM.docx]

**Additional file 8: Table S7.** Primers used for the cloning of regulatory genes of the phenylpropanoid pathway from *R. genevieri* (A) and *R. idaeus* cv. Prestige (B).

| Gene function | Transcript identified in fruit transcriptome* | | Cloned gene  name | | Forward primer** | | Reverse primer** | |
| --- | --- | --- | --- | --- | --- | --- | --- | --- |
| (A) *R. genevieri* (Rg) | | | | | | | |  |
| R2R3-type MYB TF (SG6) | [TR103098_c0_g1_i1](http://jicbio.nbi.ac.uk/cgi-bin/trinity_extract.pl?db=rubus_genevieri.fa&ref=TR103098_c0_g1_i1) | | *RgMyb10* | | 5’gcg**GGATCC**AAGGAGATAT***AACA*ATG**GAGGTGAGAAAAGGTGCATGGACGAA | | 5’cggc**CTGCAGTTA**ttgtctcgcttcttcttgtataaa | |
| R2R3-type MYB TF (SG7) | [TR71550_c1_g1_i1](http://jicbio.nbi.ac.uk/cgi-bin/trinity_extract.pl?db=rubus_genevieri.fa&ref=TR71550_c1_g1_i1) | | *RgMyb12* | | 5’gcgacg***TCTAGA***AAGGAGATAT***AACA*ATG**GGGAGGGCTCCGTGCTGTGACAAAGTT | | 5’cggc**CTCGAGTCA**AGATAAAAGCCAAGCCAGCAAAGCA | |
| PhAN1-like bHLH TF  (SG IIIf-1) | [TR110272_c1_g1_i1](http://jicbio.nbi.ac.uk/cgi-bin/trinity_extract.pl?db=rubus_genevieri.fa&ref=TR110272_c1_g1_i2) | | *RgAn1-1* | | 5’gta**GGATCC**AAGGAGATAT***AACA*ATG**GCTGCACCGCCACCGAGTAGTA | | 5’gtg**CCCGGGTTA**AGAGTCAGATTGGGGTATGACTT | |
|  |  |  | *RgAn1-2* | |  |  |  |  |
|  |  |  | *RgAn1-3* | |  |  |  |  |
| AmDEL-like bHLH TF (SGIIIf-2) | [TR110629_c1_g1_i1](http://jicbio.nbi.ac.uk/cgi-bin/trinity_extract.pl?db=rubus_genevieri.fa&ref=TR110629_c1_g1_i1) | | *RgDel* | | 5’gct**GGATCC**AAGGAGATAT***AACA*ATG**GGTACTAGGCTCCAGAACCAGGA | | 5’cggt**CTGCAGTCA**ACAATTCCTGGCGATTCTCTGAA | |
| WD40-repeat gene | [TR29409_c0_g1_i1](http://jicbio.nbi.ac.uk/cgi-bin/trinity_extract.pl?db=rubus_genevieri.fa&ref=TR29409_c0_g1_i1) | | *RgTTG1-1*  *RgTTG1-2* | | 5’gta**GGATCC**AAGGAGATAT***AACA*ATG**GGCGCAAGTAGCGATCCGAATCAGGA | | 5’gcg**ATGCATTCA**TACCCTGAGTATCTGAAGCTTGGAGGAGAA | |
| (B) *R. idaeus* cv. Prestige (Ri) | |  | |  | |  | |  |
| R2R3-type MYB TF (SG6) | [TR49283_c2_g2_i2](http://jicbio.nbi.ac.uk/cgi-bin/trinity_extract.pl?db=LIB19419-LIB19427_Trinity_NR.fasta&ref=TR49283_c2_g2_i2) | | *RiMyb10* | | 5’gcg**GGATCC**AAGGAGATAT***AACA*ATG**GAGGTGAGAAAAGGTGCATGGACGAA | | 5’cggc**CTGCAGTTA**TCTCGCTTCTTCTTGAAA | |
| R2R3-type MYB TF (SG7) | [TR1036_c0_g1_i2](http://jicbio.nbi.ac.uk/cgi-bin/trinity_extract.pl?db=LIB19419-LIB19427_Trinity_NR.fasta&ref=TR1036_c0_g1_i2) | | *RiMyb12* | | 5’gcgacg***TCTAGA***AAGGAGATAT***AACA*ATG**GGGAGGGCTCCGTGCTGTGAGAAAGTT | | 5’cggc**CTCGAGTCA**AGATAAAAGCCAAGCCAGCATAGCA | |
| PhAN1-like bHLH TF  (SG IIIf-1) | [TR75681_c0_g1_i1](http://jicbio.nbi.ac.uk/cgi-bin/trinity_extract.pl?db=LIB19419-LIB19427_Trinity_NR.fasta&ref=TR75681_c0_g1_i1) | | *RiAn1* | | 5’gta**GGATCC**AAGGAGATAT***AACA*ATG**GCTGCACCGCCACCGAGTAGTA | | 5’gtg**CCCGGGTTA**AGAGTCAGATTGGGGTATGACTT | |
| AmDEL-like bHLH TF (SGIIIf-2) | [TR16024_c0_g1_i1](http://jicbio.nbi.ac.uk/cgi-bin/trinity_extract.pl?db=LIB19419-LIB19427_Trinity_NR.fasta&ref=TR16024_c0_g1_i1) | | *RiDel-1* | | 5’gct**GGATCC**AAGGAGATAT***AACA*ATG**GGTACTAGGCTCCAGAACCAGGA | | 5’cggt**CTGCAGTCA**ACAATTCCTGGCGATTCTCTGAA | |
|  |  |  | *RiDel-2* | |  |  |  |  |
| WD40-repeat gene | [TR7065_c0_g2_i1](http://jicbio.nbi.ac.uk/cgi-bin/trinity_extract.pl?db=LIB19419-LIB19427_Trinity_NR.fasta&ref=TR7065_c0_g2_i1) | | *RiTTG1* | | 5’gcg**GGATCC**AAGGAGATAT***AACA*ATG**GACAACTCCACACAAGAATCCCATCT | | 5’gcg**ATGCATTCA**AACCTTCAAAAGCTGCATCTTGTTAGTAA | |

***** BLAST searches were undertaken using the BacHBerryGEN BLAST portal (<http://jicbio.nbi.ac.uk/berries/blast.html>).

****** Introduced restriction enzyme sites are indicated as follows: *Bam*HI (in black bold), *Xba*I (in bold italics), *Xho*I (bold and highlighted in green), *Pst*I (bold and highlighted in grey), *Nsi*I (in blue bold), *Sma*I (in red bold). Start and stop codons are highlighted in red. The gene-specific sequences are highlighted in yellow. The conserved prokaryotic ribosome binding site and plant translation initiation context sequence (Luetcke sequence) are highlighted in blue (as described in Haseloff *et al*. 1997 *PNAS* 94:2122-2127).
