# Supplementary material for: RNA-seq, de novo transcriptome assembly and flavonoid gene analysis in 13 wild and cultivated berry fruit species with high content of phenolics
Source: BMC Genomics. 2019 Dec 19;20:995. doi: 10.1186/s12864-019-6183-2 (PMC6924045; doi:10.1186/s12864-019-6183-2)
Supplement: Supplementary file 9 — Additional file 9: Figure S2. Phylogenetic relationship and protein sequence alignment of a subset of R2R3-type MYB transcription factor homologues. [file 12864_2019_6183_MOESM9_ESM.docx]

**Additional file 9: Fig. S2.** Phylogenetic relationship and protein sequence alignment of a subset of R2R3-type MYB transcription factor homologues.

**1. Phylogenetic tree of a subset of R2R3-type MYB transcription factor (TF) homologues**

**Gene name / bootstrap**

**
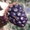

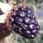

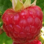

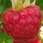
**
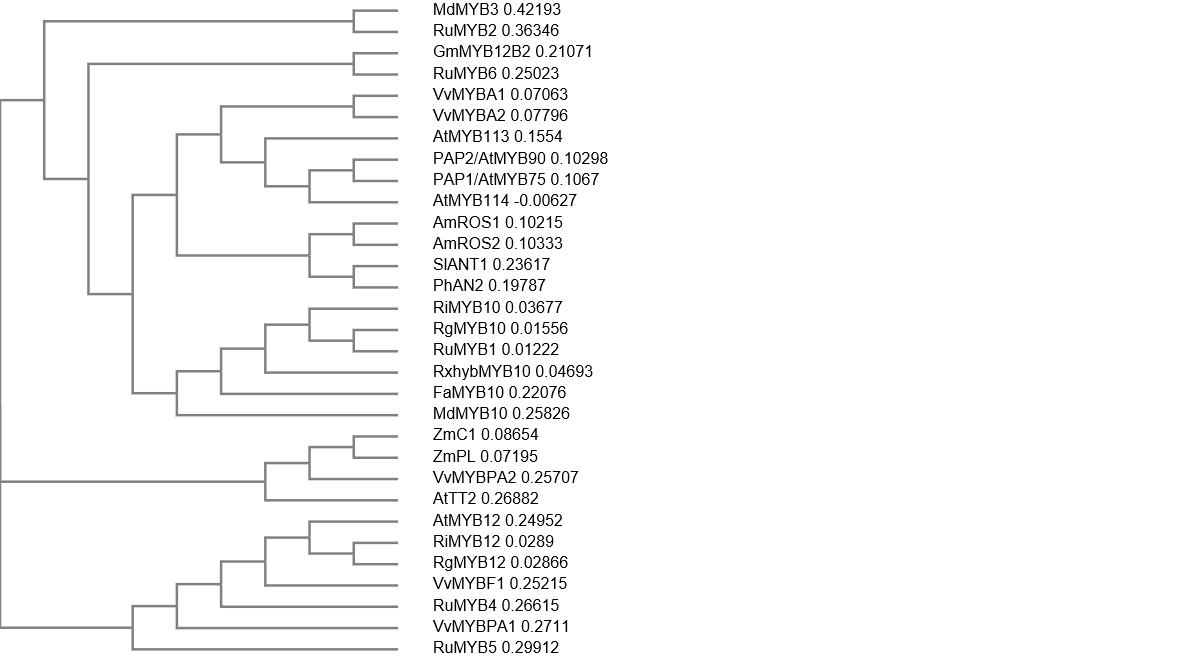


**SG6: Regulation of anthocyanin and pro-anthocyanin biosynthesis**

**SG7: Activation of flavonol and flavone biosynthesis**

The phylogenetic tree was generated using CLUSTAL OMEGA multiple sequence alignment of protein sequences. The four *Rubus* R2R3-type MYB TF homologues isolated in this study are indicated by a fruit icon. The MYB subgroups SG6 and SG7 are indicated by purple and orange boxes, respectively. The accession number or reference of each R2R3 MYB TFs is the following: *Antirrhinum majus* AmROS1 (DQ275529.1; Schwinn *et al.* (2006) Plant Cell 18:831-835), *A. majus* AmROS2 (DQ275530.1; Schwinn *et al.* (2006) Plant Cell 18:831-835), *Arabidopsis thaliana* AtMYB12 (NM_130314.4; Mehrtens *et al.* (2005) Plant Physiol. 138:1083-1096), *A. thaliana* AtMYB113 (NM105308), *A. thaliana* AtMYB114 (NM105309), *A. thaliana* AtTT2 (NM_122946.2; Nesi *et al.* (2001) Plant Cell 13:2099-2114), *Fragaria × ananassa* FaMYB10 (EU155162; Lin-Wang *et al.* (2010) BMC Plant Biol. 10:50), *Glycine max* GmMYB12B2 (JF510467.1), *Malus x domestica* MdMYB3 (HM122621), *M. domestica* MdMYB10 (AB744002; Umemura *et al.* (2013) Planta 238:65-76), *A. thaliana* PAP1/AtMYB75 (AF325123.1; Borevitz *et al.* (2000) Plant Cell 12:2383-2394), *A. thaliana* PAP2/AtMYB90 (NM_105310.4; Borevitz *et al.* (2000) Plant Cell 12:2383-2394), *Petunia x hybrida* PhAN2 (AF146702; Quattrocchio *et al.* (1999) Plant Cell 11:1433-1444), *R. genevieri* RgMYB10 (KY111315; this manuscript), *R. genevieri* RgMYB12 (KY111316; this manuscript), *R. idaeus* cv. Prestige RiMYB10 (KY111313; this manuscript), *R. idaeus* cv. Prestige RiMYB12 (KY111314; this manuscript), *Rubus* sp. var. Lochness RuMYB1 (Garcia-Seco *et al*. (2015) PLoS ONE 10(11):e0142639), *Rubus* sp. var. Lochness RuMYB2 (Garcia-Seco *et al*. (2015) PLoS ONE 10(11):e0142639), *Rubus* sp. var. Lochness RuMYB4 (Garcia-Seco *et al*. (2015) PLoS ONE 10(11):e0142639), *Rubus* sp. var. Lochness RuMYB5 (Garcia-Seco *et al*. (2015) PLoS ONE 10(11):e0142639), *Rubus* sp. var. Lochness RuMYB6 (Garcia-Seco *et al*. (2015) PLoS ONE 10(11):e0142639), *Rubus* hybrid cultivar isolate 09100351 RxhybMYB10 (JQ359611; Chen *et al.* (2012) Genet. Mol. Res. 11:1773-1782), *Solanum lycopersicum* SlANT1 (NM_001247488; Kiferle *et al.* (2015) PLoS ONE 10(8):e0136365), *Vitis vinifera* VvMYBA1 (AB242302; Kobayashi *et al.* (2004) Science 304:982), *V. vinifera* VvMYBA2 (AB097924; Kobayashi *et al.* (2004) Science 304:982), *V. vinifera* VvMYBF1 (GQ423423; Czemmel *et al.* (2009) Plant Physiol. 151:1513-1530), *V. vinifera* VvMYBPA1 (NM_001281231.1; Bogs *et al.* (2007) Plant Physiol. 143 (3), 1347-1361), *V. vinifera* VvMYBPA2 (EU919682.1; Terrier *et al.* (2009) Plant Physiol. 149:1028-1041), *Zea mays* ZmC1 (AF320614.3), *Z.mays* ZmPL (NM_001112415.1; Pilu *et al.* (2003) Plant J. 36:510-521).

**2. Protein sequence alignments of a subset of R2R3-type MYB TF homologues in comparison to the *Rubus* MYB10 and MYB12 homologues isolated in this study**

**2a. Anthocyanin- or proanthocyanidin-specific R2R3-type MYB TFs**

**List of motifs:**

**- Conserved R2 and R3 repeats**: **R2 [-W-**(x_19_)**-W-(**x_19_**)-W-]… R3 [-F/I-**(x_18_)**-W-**(x_18_)**-W-]** (Dubos *et al.*, 2010)

**- Motif for bHLH interaction**: **[D/E]L**x_2_**[R/K]**x_3_**L**x_6_**L**x_3_**R** (Zimmermann *et al.*, 2004)

**- Anthocyanin-related SG6 MYB motif:** [**R/K**]**P**x[**P/A/R**]x_2_[**F/Y**] (Lin-Wang *et al.*, 2010)

**- ‘box A’ motif of anthocyanin regulators: [A/S/G]NDV** (Lin-Wang *et al.*, 2010)

**- ‘box A’ motif of non-anthocyanin regulators: [D/E]N[E/D][I/V]** (Lin-Wang *et al.*, 2010)

**- SG5 motif: DE**x**WRL**xx**T** (Stracke *et al*., 2001)

**CLUSTAL O(1.2.4) multiple sequence alignment**

AtTT2 MGKRATTSVRREELNRGA**W**TDHEDKILRDYITTHGEGK**W**STLPNQAGLKRCGKSCRLR**W**K 60

AtMYB113 ------MGESPKGLRKGT**W**TTEEDILLRQCIDKYGEGK**W**HRVPLRTGLNRCRKSCRLR**W**L 54

PAP1/AtMYB75 ------MEGSSKGLRKGA**W**TTEEDSLLRQCINKYGEGK**W**HQVPVRAGLNRCRKSCRLR**W**L 54

PAP2/AtMYB90 ------MEGSSKGLRKGA**W**TAEEDSLLRLCIDKYGEGK**W**HQVPLRAGLNRCRKSCRLR**W**L 54

AmROS1 ------MEKNCRGVRKGT**W**TKEEDTLLRQCIEEYGEGK**W**HQVPHRAGLNRCRKSCRLR**W**L 54

AmROS2 ------MQKNPRGVRKGT**W**TKEEDILLMECIDKYGEGK**W**HQVPLKAGLNRCRKSCRLR**W**L 54

VvMYBA1 --------MESLGVRKGA**W**IQEEDVLLRKCIEKYGEGK**W**HLVPLRAGLNRCRKSCRLR**W**L 52

VvMYBA2 --------MKSLGVRKGA**W**TQEEDVLLRKCIEKYGEGK**W**HLVPLRAGLNRCLKSCRLR**W**L 52

MdMYB10 ----MEGYNENLSVRKGA**W**TREEDNLLRQCVEIHGEGK**W**NQVSYKAGLNRCRKSCRQR**W**L 56

FaMYB10 --------MEGFGVRKGA**W**TKEEDELLKQFIEIHGEGK**W**HHVPLKSGLNRCRKSCRLR**W**L 52

RiMYB10 -----------MEVRKGA**W**TKEEDHLLRNYIEKHGEGR**W**HKVPLQAGLNRCRKSCRMR**W**L 49

RxhybMYB10 -----------MEVRKGA**W**TKEEDHLLRNYIEKNGEGK**W**HNVPLNAGLNRCRKSCRMR**W**L 49

RgMYB10 -----------MEVRKGA**W**TKEEDHLLRNYIEKHGEGR**W**HKVPLEAGLNRCRKSCRMR**W**L 49

RuMYB1 -----------MEVRKGA**W**TKEEDHLLRNYIEKHGEGR**W**HKVPLEAGLNRCRKSCRMR**W**L 49

:.:*:***** .** :* : ***:***** : .:**:** **** ******

AtTT2 NYLRPGIKRGN**I**SSDEE**EL**II**R**LHN**L**LGNR**W**S**L**IAG**R**LPGRT**DNEI**KNH**W**NSNLRKRLPK 120

AtMYB113 NYLKPSIKRGKLCSDEV**DL**VL**R**LHK**L**LGNR**W**S**L**IAG**R**LPGRT**ANDV**KNY**W**NTHLSKKHDE 114

PAP1/AtMYB75 NYLKPSIKRGKLSSDEV**DL**LL**R**LHR**L**LGNR**W**S**L**IAG**R**LPGRT**ANDV**KNY**W**NTHLSKKHE- 113

PAP2/AtMYB90 NYLKPSIKRGRLSNDEV**DL**LL**R**LHK**L**LGNR**W**S**L**IAG**R**LPGRT**ANDV**KNY**W**NTHLSKKHES 114

AmROS1 NYLRPNIKRGR**F**SRDEV**DL**IV**R**LHK**L**LGNK**W**S**L**IAG**R**IPGRT**ANDV**KNF**W**NTHVGKNLGE 114

AmROS2 NYLRPNIKRGC**F**SKDEV**DL**IV**R**LHK**L**LGNK**W**S**L**IAG**R**IPGRT**ANDV**KNF**W**NTHVGKNLGV 114

VvMYBA1 NYLKPDIKRGE**F**ALDEV**DL**MI**R**LHN**L**LGNR**W**S**L**IAG**R**LPGRT**ANDV**KNY**W**HSHHFKKEV- 111

VvMYBA2 NYLKPDIKRGE**F**ALDEV**DL**MI**R**LHN**L**LGNR**W**S**L**IAG**R**LPGRT**ANDV**KNY**W**HGHHLKKKV- 111

MdMYB10 NYLKPNIKRGD**F**KEDEV**DL**II**R**LHR**L**LGNR**W**S**L**IAR**R**LPGRT**AN**A**V**KNY**W**NTRLRIDSRM 116

FaMYB10 NYLKPNIKRGE**F**AEDEV**DL**II**R**LHK**L**LGNR**W**S**L**IAG**R**LPGRT**ANDV**KNY**W**NTYQRKKDQK 112

RiMYB10 NYLKPNIKRGD**F**AEDEV**DL**MI**R**LRK**L**LGNR**W**S**L**IAG**R**LPGRT**SNDV**KNY**W**SARQRRKID- 108

RxhybMYB10 NYLKPNIKKGD**F**SENEV**DL**MI**R**LRK**L**LGNR**W**S**L**IAG**R**LPGRT**SNDV**KNY**W**SARRRRKID- 108

RgMYB10 NYLKPNIKRGD**F**AEDEV**DL**MI**R**LRK**L**LGNR**W**S**L**IAG**R**LPGRT**SNDV**KNY**W**SARRRRKTD- 108

RuMYB1 NYLKPNIKRGD**F**AEDEV**DL**MI**R**LRK**L**LGNR**W**S**L**IAG**R**LPGRT**SNDV**KNY**W**SARRRRKID- 108

***:*.**:* : :* **:***::******:.****:************* *****:**** * :**.*****

AtTT2 TQTKQP----KRIKHSTNNENNVCVI**R**TK**A**IRCSKTLLFSDLSLQKKSSTSPLPLKEQEM 176

AtMYB113 RCCKTKMINKNITSHPTSSAQKIDVL**KP**R**P**RS**F**SDKNSCNDVNILPKVDVVPLHLGLNNN 174

PAP1/AtMYB75 PCCKIKMKKRDITPIPTTPALKNNVY**KP**R**P**RS**F**TVNNDCNHLNAPPKVDVNPPCLGLNIN 173

PAP2/AtMYB90 SCCKSKMKKKNIISPPTTPVQKIGVF**KP**R**P**RS**F**SVNNGCSHLNGLPEVDLIPSCLGLKKN 174

AmROS1 DGERCR-----KNVMNTKTIKLTNIV**RP**R**A**RT**F**TGLHVTWPREVG-KTDEF--------S 160

AmROS2 DGERRK-----KNVMNTKNSKETNII**RP**R**A**RT**F**NGLHVTWPREHG-KNDAF--------S 160

VvMYBA1 --QFQE----EGRDKPQ-THSKTKAI**KP**H**P**HK**F**SKALPRFELKTT-AVDTF--------D 155

VvMYBA2 --QFQE----EGRKKPQ-THSKTKAI**KP**H**P**HK**F**SKALPRFELKTT-AVDTF--------D 155

MdMYB10 KT---------VKNKSQ-EMRETNVI**RP**Q**P**QK**F**NRSSYYLSSKEP-ILDHI--------Q 157

FaMYB10 TASYAK----KLKVKPRENTIAYTIV**RP**R**P**RT**F**IKRFNFTERYAN-IEHNH--------S 159

RiMYB10 ---FGI----PKDNEPPKITTKTTII**RP**R**P**RT**F**TKSLHHLSAKAT-TSKH---------S 151

RxhybMYB10 ---FGV----PKDNEPPKIT-KTTII**RP**R**P**RT**F**TKSLYHLSAGTA-TSLH---------S 150

RgMYB10 ---FGV----TKDNEPPKIT-KATII**RP**R**P**RT**F**TKSLHHLSTEAA-TSMH---------S 150

RuMYB1 ---FGV----PKDNEPPKIT-KTTII**RP**R**P**RT**F**TKSLHHLSAEAA-TSMH---------S 150

: :

AtTT2 DQGGSSLMGDLEFDFDRIHSEFHFPDLMDFDGLDCGNVTSLVSSNEILGELVPAQGNLDL 236

AtMYB113 YVCESSI--------------------------TCNKDEQK------------------- 189

PAP1/AtMYB75 NVCDNSI--------------------------IYNKDKKK------------------- 188

PAP2/AtMYB90 NVCENSI--------------------------TCNKDDEK------------------- 189

AmROS1 NVRLTTD-EIP----------------------DCEKQTQF------------------Y 179

AmROS2 NVRITSTTENL----------------------DYEKQKPF------------------H 180

VvMYBA1 TQVSTSSK---------------------------------------------------- 163

VvMYBA2 TQVSTSSK---------------------------------------------------- 163

MdMYB10 S---AEDL---------------------------------------------------- 162

FaMYB10 EVSYTSSL---------------------------------------------------- 167

RiMYB10 T-VENNSL---------------------------------------------------- 158

RxhybMYB10 S-VQNNSG---------------------------------------------------- 157

RgMYB10 S-EQNNSW---------------------------------------------------- 157

RuMYB1 S-EQNNSW---------------------------------------------------- 157

AtTT2 NRPFTSCHHRGD**DE**D**WLR**DF**T**C-------------------------------------- 258

AtMYB113 -DKLININLLDGDNMWWESLLE----ADVL--GPEATETAKGVTLPLDF----------- 231

PAP1/AtMYB75 -DQLVN-NLIDGDNMWLEKFLEESQEVDIL--VPEATTTEKGDTLAFDV----------- 233

PAP2/AtMYB90 -DDFVN-NLMNGDNMWLENLLGENQEADAI--VPEATTAEHGATLAFDV----------- 234

AmROS1 NDVASPQDEVEDCIQWWSKLLETTEDGELGNLFEEAQQI---GN---------------- 220

AmROS2 NNVASTPEEVDESIRWWSNLLETTED-ELENLFEDVQQT---GKMSEW------------ 224

VvMYBA1 PSS--TSPQRNDDIIWWESLLAEHAPMDQETDFSASG---EMLIASLRTEETATQKKGPM 218

VvMYBA2 PSS--TSPQPNDDIIWWESLLAEHAQMDQETDFSASG---EMLIASLWTEETATQKKGTH 218

MdMYB10 STPPQTSSSTKNGNDWWETLLEGED-TFERAAYPSIE-LEEELFTSFWFDDRLSPRSCAN 220

FaMYB10 PTEPPQTLQLENVTDWWKDFSEDSTESIDRTMCSGLGLEDHDFFTNFWVEDMLLSASN-- 225

RiMYB10 PSSPPP---IENGIDEWKT------------------LMLEDVFTNFWVEDMASLTGT-- 195

RxhybMYB10 PSASPP---IENGIDEWKT------------------LMLEDVFTNFWVEDVASLTGT-- 194

RgMYB10 PSSSPP---IENGIDEWKT------------------LMLEDVFTNFWVEDVASLTGT-- 194

RuMYB1 PSSSPP---IENGIDEWKT------------------LMLEDVFTNFWVEDVASLTGT-- 194

AtTT2 ------------------------------------------------ 258

AtMYB113 -----------------EQIWARFDEETLELN---------------- 246

PAP1/AtMYB75 -----------------DQLWSLFDGETVKFD---------------- 248

PAP2/AtMYB90 -----------------EQLWSLFDGETVELD---------------- 249

AmROS1 ------------------------------------------------ 220

AmROS2 ------------------------------------------------ 224

VvMYBA1 DGMIEQIQGGEGDFPFDVGFWDTP-------NTQ---VNHLI------ 250

VvMYBA2 SKTKA-IKPHPHKFSKALPRFELKTTAVDTFDTQVSTSSKLIHVTTTE 265

MdMYB10 --FPE--GQSRSEFSFSTDLWNHSKEE--------------------- 243

FaMYB10 -------DLVNISYV--------------------------------- 233

RiMYB10 -------GVNSAEQGFEMDLWHFFQEEAR------------------- 217

RxhybMYB10 -------GVNSAEQGFETDLWHFLQEEAR------------------- 216

RgMYB10 -------GVNSAEQGFEMDLWHFIQEEARQ------------------ 217

RuMYB1 -------GVNSAEQGFETDLWHFLQEEAR------------------- 216

**2b. Flavonol-specific R2R3-type MYB TFs**

**List of motifs:**

**- Conserved R2 and R3 repeats**: **R2 [-W-**(x_19_)**-W-(**x_19_**)-W-]… R3 [-F/I-**(x_18_)**-W-**(x_18_)**-W-]** (Dubos *et al.*, 2010)

**- Motif for bHLH interaction**: **[D/E]L**x_2_**[R/K]**x_3_**L**x_6_**L**x_3_**R** (Zimmermann *et al.*, 2004)

**- ‘box A’ motif of non-anthocyanin regulators: [D/E]N[E/D][I/V]** (Lin-Wang *et al.*, 2010)

**- R2R3-MYBs SG7 motif**: [**K/R**][**R**/x][**R/K**]x**GRT**[**S**/x][**R/G**]x_2_[**M**/x]**K** (Czemmel *et al.*, 2009)

**- SG7-2 motif:** [**W**/x][**L**/x]**LS** (Czemmel *et al.*, 2009)

**CLUSTAL O(1.2.4) multiple sequence alignment**

AtMYB12 MGRAPCCEKVGIKRGR**W**TAEEDQILSNYIQSNGEGS**W**RSLPKNAGLKRCGKSCRLR**W**INY 60

VvMYBF1 MGRAPCCEKVGLKKGR**W**TAEEDEVLVKYIQANGEGS**W**RSLPKNAGLLRCGKSCRLR**W**INY 60

RiMYB12 MGRAPCCEKVGLKKGR**W**TTEEDQTLINYIHANGEGS**W**RSLPKNAGLLRCGKSCRLR**W**INY 60

RgMYB12 MGRAPCCDKVGLKKGR**W**TTEEDQTLINYIHAHGEGS**W**RSLPKNAGLLRCGKSCRLR**W**INY 60

*******:***:*:********:***: * :**:::****************** *************

AtMYB12 LRSDLKRGN**I**TPEEE**EL**VV**K**LHSTLGNR**W**S**L**IAGHLPGRT**DNEI**KNY**W**NSHLSRKLHNFI 120

VvMYBF1 LRADLKRGN**F**SEEEE**E**III**K**LHASLGNR**W**SMIAGQLPGRT**DNEI**KNY**W**NSHLSRKVHSFR 120

RiMYB12 LRADLKRGN**I**TPQEE**D**III**K**LHASLGNR**W**S**L**IASQLPGRT**DNEI**KNY**W**NSHLSRKIDTFR 120

RgMYB12 LRADLKRGN**I**TPQEE**D**III**K**LHASLGNR**W**S**L**IAGQLPGRT**DNEI**KNY**W**NSHLSRKIDTFR 120

**:********:**: :**::::***::**********:**.:************************:..*

AtMYB12 RKPSISQDVSAVIM--------------------TNASSAPPPPQA**KRR**L**GRTSR**SA**MK**P 160

VvMYBF1 RLTNEG--------------------------PSMVIDLAKVTTAH**KRK**V**GRTSR**WA**MK**K 154

RiMYB12 RPINLMHMTSAA----TSSTSSSSSCTAAEGQMNMVMELGPPTSKQ**R**G**R**G**GRTSR**WA**MK**K 176

RgMYB12 RPINLMHMTSAAAAAATSSTSSSSSCTAAEGQMNMVMELGPPTSKR**R**G**R**G**GR**N**SR**WA**MK**K 180

* . . . **:** **:** ******.****** *******

AtMYB12 KIHRTKTRKTKK--TSAPPEPNADVAGA--DKEALMVESSGAEAELGRPCDYYGDDCNKN 216

VvMYBF1 NRSDKSIREDVNKSSLEKPKGDDDGNGVIAEKETRSETMTG---DLYA------QV-NEE 204

RiMYB12 NKIPTTGSATVTTSSSCPPPP----AAAIGQKETQT--LGG---GGGP------SD-Q-- 218

RgMYB12 NKITTNGSATVTTSSSCPPPPGPPAAAAIGQKETQT--LGG---GIGP------SD-Q-- 226

: .. . : * .. :**: * . :

AtMYB12 LMSINGDNGVLTFDDDIIDL---------------------------LLDESDPGHLYTN 249

VvMYBF1 ENPELMASRLLGCGGRMFGEGSETFGPFRVPEVEGLCF----------SENMESGVLVDG 254

RiMYB12 ---EFDDGGVVAFNDLMLDAVNEILDPTGVLTLSGKRQSDDEDVMMGVVRDEDSGKLFCG 275

RgMYB12 ---EFDDSGVVAFNDLMLDVTNEILDPTGALTLSGEGQSDDTDV-MGVVRDVDSGKLFCG 282

. :: .. ::. : : * * .

AtMYB12 TTCGGDGELHNIRD--------SEG------ARGFSDTW---------NQGNLDCLLQSC 286

VvMYBF1 GGVVSSGTEER-----------ASGATCSNKTTPFGGDVEGRNLSSDNGDQTVDQW-PSC 302

RiMYB12 PNTQEEEQLDQISPTNLSSSSNSNSISNSNSKSSYGEAA---------GAFDGEDWYNSC 326

RgMYB12 PT-QEQEQLDQISPTNLSSSSNS----NSNSKSSYGEAA---------AAYDGEDWYNSC 328

. .. : :. : **

AtMYB12 PSVE---SFLNYDH-----QVNDASTDEFIDWDCVWQEGSDNNLWHEK--ENPDSMVSWL 336

VvMYBF1 S----------------------SPTSYFDDWNWESSAVNGQELWD-----EKEEMLSWL 335

RiMYB12 SNYSLAAATCNFDDEQKQRDGGNGTLDAELRWDWESDIQRHDYIWNIDEQKDDENMLSWL 386

RgMYB12 SNYSLAAATCNFDDEQKQRDGGNGTLDAELRWDWESDIQRHDYIWNIDEQKDDENMLSWL 388

. . *: . : :*. : :.*:***

AtMYB12 LDGDDEATIGNSNCENFG--------EPLDHDDESALVA**WLLS** 371

VvMYBF1 WEDSDGGEVECET---FG--------GDLDCEKQNAMVA**WLLS** 367

RiMYB12 WEHHD------HTNNKFRSEAVVVDDQVPAHEKHNAMLA**WLLS** 423

RgMYB12 WEHDHHHHDHTNTNNKFRSEAVVVDDQVPAHEKHNALLA**WLLS** 431

: . . * :...*::*****
