# Supplementary material for: RNA-seq, de novo transcriptome assembly and flavonoid gene analysis in 13 wild and cultivated berry fruit species with high content of phenolics
Source: BMC Genomics. 2019 Dec 19;20:995. doi: 10.1186/s12864-019-6183-2 (PMC6924045; doi:10.1186/s12864-019-6183-2)
Supplement: Supplementary file 10 — Additional file 10: Figure S3. Phylogenetic relationship and protein sequence alignment of a subset of bHLH transcription factor homologues. [file 12864_2019_6183_MOESM10_ESM.docx]

**Additional file 10: Fig. S3.** Phylogenetic relationship and protein sequence alignment of a subset of bHLH transcription factor homologues.

**1. Phylogenetic tree of a subset of bHLH transcription facyor (TF) homologues**

**Gene name / bootstrap**


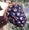

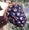

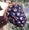

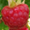

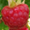

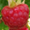

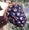

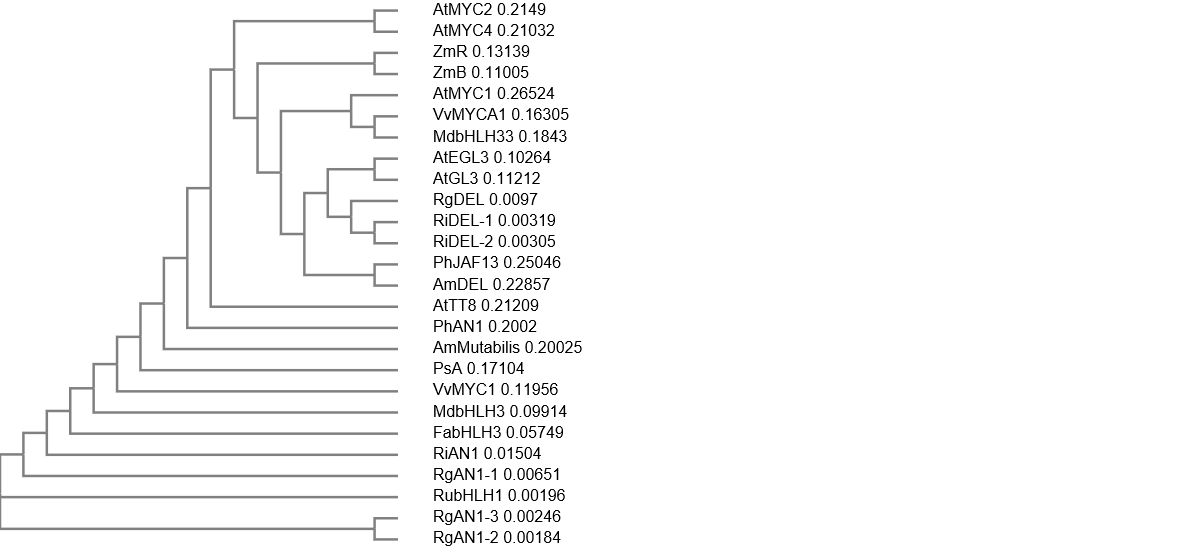


**SGIIIf-2: AmDEL/PhJAF13 clade**

**SGIIIf-1: PhAN1/AtTT8 clade**

***Rubus genevieri***

***Rubus idaeus***

**cv. Prestige**


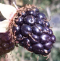

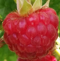


The phylogenetic tree was generated using CLUSTAL OMEGA multiple sequence alignment of protein sequences. The seven *Rubus* bHLH TF homologues isolated in this study are indicated by a fruit icon. The subgroups SGIIIf-1 (PhAN1/AtTT8 clade) and SGIIIf-2 (AmDEL/PhJAF13 clade) are indicated by purple and orange boxes, respectively. The accession number or reference of each bHLH TFs is the following: *Antirrhinum majus* AmDEL (M84913.1; Goodrich *et al.* (1992) Cell 68:955-964), *A. majus* AmMutabilis (Shang *et al*. (2011) New Phytol. 189:602-615), *Arabidopsis thaliana* AtTT8 (NM_117050.2; Nesi *et al.* (2000) Plant Cell 12:1863-1878), *A. thaliana* AtEGL3 (NP_001185302), *A. thaliana* AtGL3 (NP_680372), *A. thaliana* AtMYC1 (D83511.1; Urao *et al.* (1996) Plant Mol. Biol. 32:571-576), *A. thaliana* AtMYC2 (AAM19778.1), *A. thaliana* AtMYC4 (NM_117897), *Fragaria × ananassa* FabHLH3 (AFL02463; Schaart *et al.* (2013) New Phytol. 197:454-467), *Malus x domestica* MdbHLH3 (HM122458.1), *M. domestica* MdbHLH33 (ABB84474; Espley *et al.* (2007) Plant J. 49:414-427), *Petunia x hybrida* PhAN1 (AF260919; Spelt *et al.* (2000) Plant Cell 12:1619-1632), *P. hybrida* PhJAF13 (AF020545.1; Quattrocchio *et al.* (1998) Plant J. 13:475-488), *Pisum sativum* PsA (GU132941; Hellens *et al.* (2010) PLoS ONE 5 (10):e13230), *R. genevieri* RgAN1-1 (KY123749; this manuscript), *R. genevieri* RgAN1-2 (KY123750; this manuscript), *R. genevieri* RgAN1-3 (KY123751; this manuscript), *R. genevieri* RgDEL (KY111317; this manuscript), *R. idaeus* cv. Prestige RiAN1 (KY111320; this manuscript), *R. idaeus* cv. Prestige RiDEL-1 (KY111318; this manuscript), *R. idaeus* cv. Prestige RiDEL-2 (KY111319; this manuscript), *Rubus* sp. var. Lochness RubHLH1 (Garcia-Seco *et al.* (2015) PLoS ONE 10(11):e0142639), *Vitis vinifera* VvMYCA1 (EF193002; Matus *et al.* (2010) Plant Mol. Biol.72:607-620), *V. vinifera* VvMYC1 (EU447172; Hichri *et al*. (2010) Mol. Plant 3:509-523); *Zea mays* ZmB (X57276.1; Radicella *et al.* (1991) Plant Mol. Biol. 17:127-130), *Z. mays* ZmR (X15806.1; Perrot *et al*. (1989) Nucleic Acids Res. 17: 8003).

**2. Protein sequence alignment of a subset of plant bHLH homologues in comparison to the *Rubus* AN1- and DEL-type bHLH homologues isolated in this study**

**List of domains/motifs:**

**- Box 11, Box 18, Box 13** (Heim *et al.*, 2003)

- **bHLH domain**: **basic**[~17 aa]-**Helix 1**[~16 aa]-**Loop**[~ 6-9 aa]-**Helix 2**[~15 aa]

- **H-E-R motif**: -**H**-(x_3_)**-E-**(x_3_)**-R-** (Heim *et al.*, 2003)

- Variations of aa residues (11) between the DEL homologues RgDEL and RiDEL isolated in this study are indicated by a red bold font style.

**CLUSTAL O(1.2.4) multiple sequence alignment**

AtTT8 MDESSIIPAEK--VAGAEKKELQGLLKTAVQSVDWTYSVFWQFCP-QQRVLVWGNGYYNG 57

PhAN1 -------------------MQLQTMLRNAVQSVQWTYSLFWQLCP-QQGVLVWRDGYYNG 40

PsA -------MTAP--TPENGCNKLQNMLQAAVQSVQWTYSLFWQICP-QQLILVWGDGYYNG 50

VvMYC1 -------MAA----P--PNSRLQSMLQSAVQSVRWTYSLFWQICP-QQGILVWGDGYYNG 46

MdbHLH3 -------MAAP--PP--SSSRLRGMLQASVQYVQWTYSLFWQICP-QQGILVWSDGYYNG 48

FabHLH3 -------MATP--PP--SSSRLRGMLQSAVQSVQWTYSLFWQICP-QQGMLXXXMXYYNG 48

RiAN1 -------MAAP--PP--SSSRLRSMLQSAVQSVQWTYSLFWQICP-QQGMLIWADGYYNG 48

RgAN1-1 -------MAAP--PP--SSSRLRSMLQSAVQSVQWTYSLFWQICP-QQGMLIWADGYYNG 48

RubHLH1 -------MAAP--PP--SSSRLRSMLQSAVQSVQWTYSLFWQICP-QQGMLIWADGYYNG 48

Box 11

RgAN1-3 -------MAAP--PP--SSSRLRSMLQSAVQSVQWTYSLFWQICP-QQGMLIWADGYYNG 48

RgAN1-2 -------MAAP--PP--SSSRLRSMLQSAVQSVQWTYSLFWQICP-QQGMLIWADGYYNG 48

AtEGL3 -------MATG-ENR-TVPDNLKKQLAVSVRNIQWSYGIFWSVSASQPGVLEWGDGYYNG 51

AtGL3 -------MATG-QNRTTVPENLKKHLAVSVRNIQWSYGIFWSVSASQSGVLEWGDGYYNG 52

PhJAF13 -------MAMGCKDHNGVPDNLREQLAFAVRGIQWSYAILWSTTVSQPGELEWSDSNYNG 53

AmDEL -------MATGIQNQKIVPENLRKQLAIAVRSIQWSYAIFWSNSVAQPGVLEWGDGFYNG 53

RgDEL -------MGTRLQNQERVPENLRKQLALAVRSIQWSYAIFWSISPRQPGVLEWGDGYYNG 53

RiDEL-1 -------MGTRLQNQERVPENLRKQLALAVRSIQWSYAIFWSISPRQPGVLEWGDGYYNG 53

RiDEL-2 -------MGTRLQNQERVPENLRKQLALAVRSIQWSYAIFWSISPRQPGVLEWGDGYYNG 53

VvMYCA1 -------MANGVQNQEGVPENLSKQLAVAVRSIQWSYAIFWSLSTRQQGVLEWSGGYYNG 53

MdbHLH33 ----------MAQNHERVPGNLRKQFAVAVRSIKWSYAIFWSLSTTQQGVLEWGEGYYNG 50

.* : :*: : *:*.::*. * * ***

AtTT8 AIKTRKTTQPAEVTAEEAALERSQQLRELYETLLAGES--------TS--EARACTALSP 107

PhAN1 AIKTRKTVQPMEVSAEEASLHRSQQLRELYESLSAGES--------NQP-TRRPSAALSP 91

PsA AIKTRKTVQPMEVSAEEASLQRSQQLRELYESLSAGET--------NPP-TRRPCASLSP 101

VvMYC1 AIKTRKTVQPMEVSAEEASLQRSQQLRELYESLSAGET--------NQP-ARRPCAALSP 97

MdbHLH3 AIKTRKTVQPMEVSADEASLQRSQQLRELYDSLSAGET--------NQPPARRPCASLSP 100

FabHLH3 AIKTRKTVQPMEVSSEEASLQRSQQLRELYDTLSAGET--------NQP-ARRPCAALSP 99

RiAN1 AIKTRKTVQPMEVSSEEASLQRSQQLRELYDSLSAGET--------NQP-ARRPCAALSP 99

RgAN1-1 AIKTRKTVQPMEVSSEEASLQRSQQLRELYDSLSAGET--------NQP-ARRPCAALSP 99

Box 18

RubHLH1 AIKTRKTVQPMEVSSEEASLQRSQQLRELYDSLSAGET--------NQP-ARRPCAALSP 99

RgAN1-3 AIKTRKTVQPMEVSSEEASLQRSQQLRELYDSLSAGET--------NQP-ARRPCAALSP 99

RgAN1-2 AIKTRKTVQPMEVSSEEASLQRSQQLRELYDSLSAGET--------NQP-ARRPCAALSP 99

AtEGL3 DIKTRKTIQAAEVKIDQLGLERSEQLRELYESLSLAESSASG---SSQVTRRASAAALSP 108

AtGL3 DIKTRKTIQASEIKADQLGLRRSEQLSELYESLSVAESSSSGVAAGSQVTRRASAAALSP 112

PhJAF13 DIKTRKTVQAGEVDEDQLGLQRTEQLRDLYSSLLIGEGEED-----LQPQAKRPSAALSP 108

AmDEL DIKTRKTVQSVELNQDQLGLQRSDQLRELYESLSLGET---------NTQAKRPTAALSP 104

RgDEL DIKTRKTVQAIELNADQMGLQRSEHLRELYESLSAGEA---------SPQARRPSAALSP 104

RiDEL-1 DIKTRKTVQAIELNADQMGLQRSEHLRELYESLSAGEA---------SPQARRPSAALSP 104

RiDEL-2 DIKTRKTVQALELNADQMGLQRSEHLRELYESLSAGEA---------SPQARRPSAALSP 104

VvMYCA1 DIKTRKTVQEMELKADKMGLQRSEQLRELYESLLEGET---------DQQSKRPSAALSP 104

MdbHLH33 DIKTRKKVEGVELKTDKMGLQRNVQLRELYKSLLEGETE-------TEQQAKAPSAVLSP 103

*****. : *: :: .*.*. :* :**.:* .* : ***

AtTT8 EDLTETEWFYLMCVSFSFPPPSGMPGKAYARRKHVWLSGANEVDSKTFSRAILAKSAKIQ 167

PhAN1 EDLTESEWFYLMCVSFSFPAGIGLPGKAYSKKHHIWITGANEVESKVFCRAILAKSARVQ 151

Box 13

PsA EDLTESEWFYLMCVSFSFPPGVGLPGKAYARRQHVWLTGANEVDSKTFSRAILAKSANIQ 161

VvMYC1 EDLTESEWFYLMCVSFSFPPGVGLPGKAYAKRHHIWLAGANEVDSKVFSRAILAKSARVQ 157

MdbHLH3 EDLTESEWFYLMCVSFSFPPGVGLPGKAYARRQHVWLTGANEVDSKTFSRAILAKSARIQ 160

FabHLH3 EDLTESEWFYLMCVSFSFPPGVGLPGKAYSRRQHVWLTGANEVDSKTFSRAILAKSARVQ 159

RiAN1 EDLTESEWFYLMCVSFSFPPGVGLPGKAYTRRQHVWLTGANEVDSKTFSRAILAKSARVQ 159

RgAN1-1 EDLTESEWFYLMCVSFSFPPGVGLPGKAYTRRQHVWLTGANEVDSKTFSRAILAKSARVQ 159

RubHLH1 EDLTESEWFYLMCVSFSFPPGVGLPGKAYTRRQHVWLTGANEVDSKTFSRAILAKSARVQ 159

RgAN1-3 EDLTESEWFYLMCVSFSFPPGVGLPGKAYTRRQHVWLTGANEVDSKTFSRAILAKSARVQ 159

RgAN1-2 EDLTESEWFYLMCVSFSFPPGVGLPGKAYTRRQHVWLTGANEVDSKTFSRAILAKSARVQ 159

AtEGL3 EDLTDTEWYYLVCMSFVFNIGEGIPGGALSNGEPIWLCNAETADSKVFTRSLLAKSASLQ 168

AtGL3 EDLADTEWYYLVCMSFVFNIGEGMPGRTFANGEPIWLCNAHTADSKVFSRSLLAKSAAVK 172

PhJAF13 EDLTDTVWYFLVCMSFVFNVGQGLPGKSLARHETIWLCNAHQAESSVFSRSLIAKSASIQ 168

AmDEL EDLTDAEWFFLVCMSFIFNIGQGLPGRTLARNQAVWLCNAHRADTKVFSRSLLAKSASIQ 164

RgDEL EDLADTEWYYLVCMSFVFNIGQGLPGRTLANGQPIWL**C**NAHYADS**R**VFSRSLLAKSASIQ 164

RiDEL-1 EDLADTEWYYLVCMSFVFNIGQGLPGRTLANGQPIWL**Y**NAHYADS**K**VFSRSLLAKSASIQ 164

RiDEL-2 EDLVDTEWYYLVCMSFVFNIGQGLPGRTLANGQPIWL**Y**NAHYADS**K**VFSRSLLAKSASIQ 164

VvMYCA1 EDLSDAEWYYLVCMSFVFNPGEGLPGRALANGQSIWLCDAQYADSKVFSRSLLAK----- 159

MdbHLH33 EDLTDAEWYYLLCMSFIFNPGEGLPGRALATGQTIWLCNAQHTDSKVFSRSLLAKSASVQ 163

*** :: *::*:*:** * *:** : : . :*: .*. .:: .* *:::**

AtTT8 TVVCIPMLDGVVELGTTKKVREDVEFVELTKSFFYDHC--KTNPKPALSEHSTYEVHEE- 224

PhAN1 TVVCIPLLDGVVELGTTQRIQEDIGFINHVKTFFIEQQ-PPLPPKPALSEHSTSNPTTFS 210

PsA TVVCIPVLDGVVEIGTTDKIQEDLNFIKHVRSFFIDHH--SLPPKPALSEHSTSNPTYST 219

VvMYC1 TVVCIPLMDGVVEFGTTEKVQEDLGFVQHVKSFFTDHHLHNHPPKPALSEHSTSNPATSS 217

MdbHLH3 TVVCIPLLDGVVEFGTTERVPEDHAFVEHVKTFFVDHH-HPPPPKPALSEHSTSNPAASS 219

FabHLH3 TVVCIPLLDGVVELGTTDRVPEDLAFVQHVKTFFVDHH-HLPPPKPALSEHSTSNPATSS 218

RiAN1 TVVCIPILDGVVELGTTDRVPEDLAFVQHVKTFFVDNH-HLPPPKPALSEHSTSNPATSS 218

RgAN1-1 TVVCIPMLDGVVELGTTDRVPEDLAFVQHVKTFFVDHH-HVPPPKPALSEHSTSNPSTSS 218

RubHLH1 TVVCIPMLDGVVELGTTDRVPEDLAFVQHVKTFFVDHH-HVPPPKPALSEHSTSNPSTSS 218

RgAN1-3 TVVCIPMLDGVVELGTTDRVPEDLAFVQHVKTFFVDHH-HVPPPKPALSEHSTSNPSTSS 218

RgAN1-2 TVVCIPMLDGVVELGTTDRVPEDLAFVQHVKTFFVDHH-HVPPPKPALSEHSTSNPSTSS 218

AtEGL3 TVVCFPFLGGVLEIGTTEHIKEDMNVIQSVKTLFLEAPP-YTTI-STRSDYQ--EIF--- 221

AtGL3 TVVCFPFLGGVVEIGTTEHITEDMNVIQCVKTSFLEAPDPYATILPARSDYHIDNVLD-- 230

PhJAF13 TVVCFPYLGGVIELGVTELVVEDPNLIQQIKISILKVDHSIIPKRPNYVSSDAKNDAIGL 228

AmDEL TVVCFPYSEGVVELGATELVPEDLNLIQHIKTSFLDSPATVPK-IPNYVSNSITNNNDLI 223

RgDEL TVVCFPYLRGVIELGVTELVLEDPGLIQHVKTSFLEVPYPLSSKKTNPSAGIYRNEK**D**LA 224

RiDEL-1 TVVCFPYLRGVIELGVTELVLEDPGLIQHVKTSFLEVPYPLSSKKTNPSAGIYRNEK**V**LA 224

RiDEL-2 TVVCFPYLRGVIELGVTELVLEDPGLIQHVKTSFLEVPYPLSSKKTNPSAGIYRNEK**V**LA 224

VvMYCA1 TVVCFPHMGGVIELGVTELVPEDPSLIQHIKACLLELSKPICSEKSSFVPCNTDDDKDRM 219

MdbHLH33 TVVCFPYLGGVVELGVTELVSEDLNLIQHIKASLLDFSKPDCCEKSSSAPHKPDDDSEQI 223

****:* **:*:*.*. : ** .:: : : . :

AtTT8 -------------------------------------------AEDEEEV---------- 231

PhAN1 ELN--FYSSN------------TPPSAGTTPADEHGGVAGDEDEEDEDEEDEDEEQEDDE 256

PsA DHIPAIMYTVA------------DPASTAIPNQ-------DDMDEDEEEDDEDDEVESGS 260

VvMYC1 DH-SRFHSPPIQAAYAAA-----DPPASNNQEEEEEEEEEEEEEEEEEEEEEEEEAESDS 271

MdbHLH3 D-HPHFHSPHLLQAM------CTNPPLNAAQED------EEDEEEDDNQEEDDGGAESDS 266

FabHLH3 DHHPRFHSPHLTAISNNAA-ANNPPPLHAAE-Q------EDDENEDE-------EGESDS 263

RiAN1 DHHPRFHSPNLTDIRNNAAAANNAPPLHAAA-Q------EDEEDEDEEEDDQDDEAESDS 271

RgAN1-1 DHHPRFNSPHLTAIRNNAAAANNPPPLHAAA-Q------EDEEDDDEEEDDQDDEAESDS 271

RubHLH1 DHHPRFNSPHLTAIRNNAAAANNPPPLHAAA-Q------EDEEDDDEEEDDQDDEAESDS 271

RgAN1-3 DHHPRFNSPHLTAIRNNAAAANNPPPLHAAA-Q------EDEEDDDEEEDDQDDEAESDS 271

RgAN1-2 DHRPRFNSPHLTAIRNNAAAANNPPPLHAAA-Q------EDEEDDDEEEDDQDDEAESDS 271

AtEGL3 ------------DPLSDDKYTPVF-----------------------ITEAFPTTST--- 243

AtGL3 ----------PQQILGDEIYAPMF-----------------------STEPFPTASPSRT 257

PhJAF13 C------PKPDHNVLENDAY----------------------------TVEINNSSPHDS 254

AmDEL C------EALEHANIPENDLDQLL-----------------------NCPDTNICSPDNS 254

RgDEL G------SALDQDVIDTKFIPVI------------------------RCEEID**GT**SL**S**NS 254

RiDEL-1 G------SALDQDVIDTKFIPVI------------------------RCEEID**VA**SL**N**NS 254

RiDEL-2 G------SALDQDVIDTKFIPVI------------------------RCEEID**VA**SL**N**NS 254

VvMYCA1 C------AKVDHDIVETMALEKLYPATEEIKFEQE-GMSEL---HGNIHEEHNIGSPDDC 269

MdbHLH33 V------AKVDHDVVDTLPLENLYSPSEEIKFDQR-GINGL---LGN-HEEVNMDSSDEC 272

AtTT8 --------------------------------EEEMTMSEEMRLGSPDDEDVSNQNLHSD 259

PhAN1 EAELDSD------KIAAQVGPADVIAAAEASELMQLDMSEAIRFGSPDDG--SNTNMDSD 308

PsA EDETNQGHN---------QHATSIIEAAEPSELMQIEMPDDIRIGSPNDG--SN-NLDSD 308

VvMYC1 EAETGRNNRRVRTQNTGTEGVAGSHTAAEPSELIQLEMSEGIRLGSPDDG--SN-NLDSD 328

MdbHLH3 EAETGRNGGAVVPAANP---PQVLAAVAEPSELMQLEMSEDIRLGSPDDA--SN-NLDSD 320

FabHLH3 EAETGRNGNGSPLPPA-----GTNVPSAEPSELMQLEMSEDIRLGSPDDA--SN-NLDSD 315

RiAN1 EAETGRN--GRVLVAA-----GSNIPAAEPSELMQLEMSEDIRLGSPDDA--SN-NLDSD 321

RgAN1-1 EAETGRN--GRALVAA-----GPNVPAAEPSELMQLEMSEDIRLGSPDDA--SN-NLDSD 321

RubHLH1 EAETGRN--GRALVAA-----GPNVPAAEPSELMQLEMSEDIRLGSPDDA--SN-NLDSD 321

RgAN1-3 EAETGRN--GRALVAA-----GPNVPAAEPSELMQLEMSEDIRLGSPDDA--SN-NLDSD 321

RgAN1-2 EAETGRN--GRALVAA-----GPNVPAAEPSELMQLEMSEDIRLGSPDDA--SN-NLDSD 321

AtEGL3 -SGFEQE----P-------------------EDHDSFIN---DGGASQVQ--SWQFVGEE 274

AtGL3 TNGFDQEHEQVA-------------------DDHDSFMTERITGGASQVQ--SWQLMDDE 296

PhJAF13 SNGFGANQ-----------------------EVEVSLMVVGVIGETSQAQ--SWKFTDDN 289

AmDEL LDDFADNL-----------------------LIDESNLAEGINGEVPQTQ--SWPFMDDA 289

RgDEL SNGFGP**N**Q-----------------------PAEDSFMVEGMNGGASQVQ--SWQYLDDE 289

RiDEL-1 SNGFGP**D**Q-----------------------PAEDSFMVEGMNGGASQVQ--SWQYLDDE 289

RiDEL-2 SNGFGP**D**Q-----------------------PAEDSFMVEGMNGGASQVQ--SWQYLDDE 289

VvMYCA1 SNGCEDDH-----------------------QTEDSFMLEGINGGASQVQ--SWHFVDDD 304

MdbHLH33 SNGCDHNH-----------------------PTEDSMMLEGTNAVASQVQ--SWHFMDED 307

: : * .

AtTT8 LHIESTHTLDTHM---------------------------------------------DM 274

PhAN1 FHMVGVSQAENPADYQRQAESFKADT----SISWAHFQDLPH-----------LPGGPSY 353

PsA FHLLAVSNQGNPSR---QIDSY---T----TERWGPIEEPLDDSLQIQL--SSSVLHHPL 356

VvMYC1 FHMLAVSQPGSSVDHQRRADSYRAES----ARRWPMLQDPLCSSGLQQPPPQPPTGPPPL 384

MdbHLH3 FHLLAVSQSRNPADQQRQADSYRAES----TRRRPSVQEPLSS-GLQ----PPHTGPLAL 371

FabHLH3 FHMLAVSQSANAADQQRQADSYRAES----ARRWPPVQRPMS--AVQ----PPPSGSLEL 365

RiAN1 FNMLAVSQSADQ--QRQAADSYRAES----TRRWPPVQQPLS--GVE----PPPLGPLAL 369

RgAN1-1 FNMLAVSQSADQ--QRQAADSYRAES----TRRWPPVEQPMS--GVQ----PPPLGSLAL 369

RubHLH1 FNMLAVSQSADQ--QRQAADSYRAES----TRRWPPVEQPMS--GVQ----PPPLGSLAL 369

RgAN1-3 FNMLAVSQSADQ--QRQAADSYRAES----TRRWPPVEQPMS--GVQ----PPPLGSLAL 369

RgAN1-2 FNMLAVSQSADQ--QRQAADSYRAES----TRRWPPVEQPMS--GVQ----PPPLGSLAL 369

AtEGL3 IS-NCIHQSLNSSDCVSQTFV-GTTGRLACDPRKSR----IQ--RLGQIQEQ-SNHVN-- 323

AtGL3 LS-NCVHQSLNSSDCVSQTFVEGAAGRVAYGARKSR----VQ--RLGQIQEQ-QRNVKTL 348

PhJAF13 MS-NGVHNSLNSSDCTSQNYANCEKL--SPLSSGEKETKP----APLDHQEHNQRKLHLL 342

AmDEL IS-NCLNSSMNSSDCISQTHENLESF--APLSDGKGPPETNN--CMHSTQKCNQQ---IE 341

RgDEL LS-NYVHHSMDSSDCISQTLVYPEKV--VSGPKGEKVVS-DH--FLQDNKECNSTKQTS**L** 343

RiDEL-1 LS-NYVHHSMDSSDCISQTLVYPEKV--VSGPKGEKVVS-DH--FLQDNKECNSTKQTS**M** 343

RiDEL-2 LS-NYVHHSMDSSDCISQTLVYPEKV--VSGPKGEKVVS-DH--FLQDNKECNSTKQTS**M** 343

VvMYCA1 FS-NGVQGSMDSSDCISQAFVNQERI--HSSPKGENVNN-V---RLKDLQECNDTKFSSL 357

MdbHLH33 FS-SGVQDSMNSSDSISEAFVNQGKA--HSFAKHENANH-I---HLKELQNFNDTKLSSL 360

:

AtTT8 MNL-MEEGGNYSQTVTTLLMSHPTSLLSDSV-----STSSYIQSSFATWRVENGKEHQ-- 326

PhAN1 DELSQ-EDTHYSQTVSTILEHLSNQSSKFSSTIMGC-ISQTTQSAFTRWPSPSTTVSSPF 411

PsA EDLTQ-EDTHYSQTVTTILQNQ---WIDSP--SIN-YINYSTQSSFTTWTNHH--FHP-P 406

VvMYC1 DELSQ-EDTHYSQTVSTILQHQPNRWSESS--SSGCIAPYSSQSAFAKWTTRCDHHHH-- 439

MdbHLH3 EELTHDDDTHYSETVSTILQGQVTQLMDSS--STD-YTACLTQSAFAKWSSRVDH--H-F 425

FabHLH3 EELNHD-DTHYSETVSTILQTQATRWTDSS--SND-YVTYSIQSAFAKWTNRADH--H-L 418

RiAN1 EELTHD-DTHYSETVSTILQTQATRRTDSS--SND-YVTYSSQSAFAKWTGRGDHH-H-L 423

RgAN1-1 EELTHD-DTHYSETVSTILQNQSTRRTDSS--SND-YVTYSSQSAFAKWTSRGDHH-Q-L 423

RubHLH1 EELTHD-DTHYSETVSTILQNQSTRRTDSS--SND-YVTYSSQSAFAKWTSRGDHH-H-L 423

RgAN1-3 EELTHD-DTHYSETVSTILQNQATRRTDSS--SND-YVTYSSQSAFAKWTSRGDHH-H-L 423

RgAN1-2 EELTHD-DTHYSETVSTILQNQSTRRTDSS--SND-YVTYSSQSAFAKWTSRGDHHHH-L 424

AtEGL3 ----MDDDVHYQGVISTIFKTTHQ------LILGPQFQNFDKRSSFTRWKRSSSV----- 368

AtGL3 SFDPRNDDVHYQSVISTIFKTNHQ------LILGPQFRNCDKQSSFTRWKKSSSSSSG-- 400

PhJAF13 D--HQGDETQYQSVLSTLLKSSDQ------LTLGPYFRNTNKRSCFSGWKNDA----H-- 388

AmDEL NTGVQGDEVHYQGVLSNLLKSSHQ------LVLGPYFRNGNRESSFVSWNKDGSSGTH-- 393

RgDEL EP--QSNDLHYQSVLSSLLKSSHQ------LILGPHFQNG**L**QESSFVSWKKGGSVKCQ-- 393

RiDEL-1 EP--QSNDLHYQSVLSSLLKSSHQ------LILGPHFQNG**H**QESSFVSWKR-GSVKCQ-- 392

RiDEL-2 EP--QSNDLHYQSVLSSLLKSSHQ------LILGPHFQNG**H**QESSFVSWKKGGSVKCQ-- 393

VvMYCA1 DLGA-DDDLHYRRTISTVLRKSHP------LIGNSCFRCYDIKSSFITWKKGGMLDAQ-- 408

MdbHLH33 YLGSVDEHVHYKRTLCTLLGSSMK------LIENPCFCDGESKSSFVKWKKEV-VGSC-- 411

:* .: .:: .*.* *

AtTT8 QVKTAPSSQWVLKQMIFRVPFLHDNTKDK--RL------------------------PRE 360

PhAN1 LDGGATSGQWLLKSILFSVPFLHTKYQTAAEVSPKSRDATTVDSSTASRFRKG-CSITQE 470

PsA PPPDPATSQWLVKYILFTVPYLHTKNHDE--TSPQTRDTAGVNSNDPSARLRG-KGTPQD 463

VvMYC1 PMAVEGTSQWLLKYILFSVPFLHTKYRDE--NSPK-SRD-----GDS-AGRFR-KGTPQD 489

MdbHLH3 LMPVEGTSQWLLKYILFSVPFLHSKYRDE--NSPK-FQE-----GEG-STRLR-KGTPQD 475

FabHLH3 LMPVEGTSQWLLKYILFSVPFLHTKYRDE--NSPKSSHD-----GEG-STRLR-KGTSQD 469

RiAN1 LVPVEGTSQWLLKYILFSVPFLHTKYRDE--NSPK-SHD-----GDG-STRLR-KGTPQD 473

RgAN1-1 LVPVEGTSQWLLKYILFSVPFLHSKYRDE--NSPK-SHD-----GDG-STRLR-KGTPQD 473

RubHLH1 LVPVEGTSQWLLKYILFSVPFLHSKYRDE--NSPK-SHD-----GDG-STRLR-KGTPQD 473

RgAN1-3 LVPVEGTSQWLLKYILFSVPFLHSKYRDE--NSPK-SHD-----GDG-STRLR-KGTPQD 473

RgAN1-2 LVPVEGTSQWLLKYILFSVPFLHSKYRDE--NSPK-SHD-----GDG-STRLR-KGTPQD 474

AtEGL3 -KTLGEKSQKMIKKILFEVPLMNKKEEL------------------------LPDTP--- 400

AtGL3 TATVTAPSQGMLKKIIFDVPRVHQKEKL------------------------MLDSPEAR 436

PhJAF13 I-PSRGTAQKLLKKVLVEVPRMHGSVI-H--KFSREN-----------RKRNGLWRPEVD 433

AmDEL V-PRSGTSQRFLKKVLFEVARMHENSR-L--DAGKQK-----------GNSDCLAKPTAD 438

RgDEL K-QQGGSPQYLLKKILFEVPRMHVVCV-L--ESPEDN-----------GDRNGVWRPEAG 438

RiDEL-1 K-QQGGSPQYLLKKILFEVPRMHVVCV-L--ESPEDN-----------GDRNGVWRPEAG 437

RiDEL-2 K-QQGGSPQYLLKKILFEVPRMHVVCV-L--ESPKDN-----------GDRNGVWRPEAG 438

VvMYCA1 K-PQ--TQQRILKKILFTVPLMHGGCG-F--KSQKEN-----------AGRDGLWKSGSD 451

MdbHLH33 R-PT--VHQKTLKKILFTVPLMYGVHSPM--ATGKEN-----------TGKDLLPNLQGD 455

* :* ::. * :

**Basic Helix 1 Loop Helix 2**

AtTT8 --DLS**H**VVA**E**RRR**R**EKLNEKFITLRSMVPFVTKMDKVSILGDTIAYVNHLRKRVHELENT 418

PhAN1 EPSGN**H**VLA**E**RRR**R**EKLNERFIILRSLVPFVTKMDKASILGDTIEYVKQLRKKVQDLEAR 530

PsA ELSAN**H**VLA**E**RRR**R**EKLNERFIILRSLVPFVTKMDKASILGDTIEYLKQLRRKIQDLETR 523

VvMYC1 ELSAN**H**VLA**E**RRR**R**EKLNERFIILRSLVPFVTKMDKASILGDTIEYVKQLRKKIQDLEAR 549

MdbHLH3 ELSAN**H**VLA**E**RRR**R**EKLNERFIILRSLVPFVTKMDKASILGDTIEYVKQLRNKIQDLEAR 535

FabHLH3 ELSAN**H**VLA**E**RRR**R**EKLNERFIILRSLVPFVTKMDKASILGDTIEYVKQLRKKIKDLEAR 529

RiAN1 ELSAN**H**VLA**E**RRR**R**EKLNERFIILRSLVPFVTKMDKASILGDTIEYVKQLRKKIQDLEAR 533

RgAN1-1 ELSAN**H**VLA**E**RRR**R**EKLNERFIILRSLVPFVTKMDKASILGDTIEYVKQLRKKIQDLEAR 533

RubHLH1 ELSAN**H**VLA**E**RRR**R**EKLNERFIILRSLVPFVTKMDKASILGDTIEYVKQLRKKIQDLEAR 533

RgAN1-3 ELSAN**H**VLA**E**RRR**R**EKLNERFIILRSLVPFVTKMDKASILGDTIEYVKQLRKKIQDLEAR 533

RgAN1-2 ELSAN**H**VLA**E**RRR**R**EKLNERFIILRSLVPFVTKMDKASILGDTIEYVKQLRKKIQDLEAR 534

AtEGL3 EETGN**H**ALS**E**KKR**R**EKLNERFMTLRSIIPSISKIDKVSILDDTIEYLQDLQKRVQELESC 460

AtGL3 DETGN**H**AVL**E**KKR**R**EKLNERFMTLRKIIPSINKIDKVSILDDTIEYLQELERRVQELESC 496

PhJAF13 DTDRSRVIS**E**RRR**R**EKINERFMLLASMLPAGGKVDKISLLDETIEYLKELERRVQDLE-- 491

AmDEL EIDRN**H**VLS**E**RKR**R**EKINERFMILASLVPSGGKVDKVSILDHTIDYLRGLERKVDELESN 498

RgDEL ESLMN**H**VLS**E**RKR**R**EKLNERFSILKSMVP**S**IRKDDKVSILDDAIEYLKDLEKRVAELETS 498

RiDEL-1 ESLMN**H**VLS**E**RKR**R**EKLNERFSILKSMVP**A**IRKDDKVSILDDAIEYLKDLEKRVAELETS 497

RiDEL-2 ESLMN**H**VLS**E**RKR**R**EKLNERFSILKSMVP**A**IRKDDKVSILDDAIEYLKDLEKRVAELETS 498

VvMYCA1 GICKQ**H**ALSDKKR---EKEKFLVLRSMVPSINKIDEVSILGDTIEYLKKLEARVEELETS 508

MdbHLH33 DINRE**H**---DKMR---ENAKLLVLRSMVPSITEVDKASILDDTIKYLKELEARAEEMESC 509

. :: * : :: * .::* : *: *:*..:* *:. *. : ::*

AtTT8 HHEQQHKR--TRT----------------------------------------------- 429

PhAN1 ANQTEATLQ-TKDTG--------------------TVKVLQGRGKRRMKIVEGSVGGGQA 569

PsA NRQMESEKSGVT-------------------------VLVGPTEKKKVRIVEGNGTGGGV 558

VvMYC1 TRQMEVEQR-SRGS----DSVRSKEHRIGSGSVDRNRAVVAGSDKRKLRIVEGSTGAK-- 602

MdbHLH3 NMLVEEDQ-RSRSSGEMQRSNSCKELRSGLTLVERTQGGPPGSDKRKLRIVEGSGGVAIG 594

FabHLH3 NVHLEDDQQHTRSLGEIQRSSSMKELRSALTVTERSRVGSPGSDKRKLRIVEGSGGVAVA 589

RiAN1 NVHLEDDQ-RTRSLGEIQRSSSMKELRSGG--LDRTRTGPPGSDKRKLRIVEGSGGAAVA 590

RgAN1-1 NVLLEDDQ-RTRSLGEIQRSSSMKDLRSGG--MDRPRTGPPGSDKRKLRIVEGSGGAAVA 590

RubHLH1 NVHLEDDQ-RTRSLGEIQRSSSMKELRSGG--LDRSRTAPPGSDKRKLRIVEGSGGAAVA 590

RgAN1-3 NVHLEDDQ-RTRSLGEIQRSSSMKELRSGG--LDRSRTGPQGSDKRKLRIVEGSGGAAVA 590

RgAN1-2 NVHLEDDQ-RTRSLGEIQRSSSMKELRSGG--LDRSRTGPPGSDKRKLRIVEGSGGAAVA 591

AtEGL3 RESADTETRITM-M---KRKKPD-------DEEERASANC---MNSKR----KG---SDV 499

AtGL3 RESTDTETRGTMTM---KRKKPC-------DAGERTSANC---ANNETGNGKKV---SVN 540

PhJAF13 ------------AK---SGRRPN-------DVAEQTSDNC---GTSKFNAIEES---LPN 523

AmDEL KMVKGRGRE---ST---TKTKLH-------DAIERTSDNY---GATRTSNVKKP---LTN 539

RgDEL RES--TDFE---AK---TKR**N**PQ-------ENSERTSDNC---CNSKMSNGKKP---LVY 537

RiDEL-1 RES--TDFE---AK---TKR**K**PQ-------ENSERTSDNC---CNSKMSNGKKP---LVY 536

RiDEL-2 RES--TDFE---AK---TKR**K**PQ-------ENSERTSDNC---CNSKMSNGKKP---LVY 537

VvMYCA1 MDLQ-TELD---AR---ARQKYL-------DMVEQTSDNY---DDKMIDDGKKL---WIN 548

MdbHLH33 MDTV----E---AI---SRGKFL-------NRVEKTSDNY---DKTKKNNVKKS---LVK 546

AtTT8 ------------------------CKRKTSEEVEVSIIENDVLLEMRCEYRDGLLLDILQ 465

PhAN1 KITA--SSPS-------------TTHEEEIVQVEVSIIESDALVELRCPYKEGLLLDVMQ 614

PsA RAKAV----------------------EVVASVQVSIIESDALLEIECLQREGLLLDVMM 596

VvMYC1 -PKVVDSPPAA--------------VEGGTTTVEVSIIESDALLEMQCPYREGLLLDVMQ 647

MdbHLH3 KAKVMEDSPPSPPPPPPQPEPL-PTPMVTGTSLEVSIIESDGLLELQCPYREGLLLDVMR 653

FabHLH3 KPKVVENRHS-SATTAPEP-AP-PMPMLTGTSLEVSIIESDGLLELHCPYREGLLLDVMQ 646

RiAN1 KPKVIEEAPPPPPPQAPEP-SL-PTPMLTGTSLEVSIIESDGLLELQCPYREGLLLDVMQ 648

RgAN1-1 KPKVIEEAPPPPPPQAPEP-SL-PTPMLTGTSLEVSIIESDGLLELQCPYREGLLLDVMQ 648

RubHLH1 KPKVIEEAPPPPPPQAPEP-SL-PTPMLTGTSLEVSIIESDGLLELQCPYREGLLLDVMQ 648

RgAN1-3 KPKVIEEAPPAPPPQAPEP-SL-PTPMLTGTSLEVSIIESDGLLELQCPYREGLLLDVMQ 648

RgAN1-2 KPKVIEEAPPAPPPQAPEP-SL-PTPMLTGTSLEVSIIESDGLLELQCPYREGLLLDVMQ 649

AtEGL3 NVGEDE-----PADIG--------YAGL-TDNLRISSLGNEVVIELRCAWREGILLEIMD 545

AtGL3 NVGEAE-----PADTG--------FTGL-TDNLRIGSFGNEVVIELRCAWREGVLLEIMD 586

PhJAF13 KRKACE-----IVDLEPESRNGLLKGSS-TDSIVINMIDKEVSIKMRCLSSEGLLFKIME 577

AmDEL KRKASD-----TDKIGAVNSRGRLKDSL-TDNITVNITNKDVLIVVTCSSKEFVLLEVME 593

RgDEL KRKACD-----IDETETETNYVVSKNGS-SDNVKVSMNNKGALIEMRFPWREGVLLEVMD 591

RiDEL-1 KRKACD-----IDETETETNYVVSKNGS-SDNVKVSMNNKGALIEMRFPWREGVLLEVMD 590

RiDEL-2 KRKACD-----IDETETETNYVVSKNGS-SDNVKVSMNNKGALIEMRFPWREGVLLEVMD 591

VvMYCA1 KRKACD-----IDETDLEINEIIPKDSLPSSDMKVRINEQEVLIEMRCPWREYLLLDIMD 603

MdbHLH33 KRKACD-----IDETDPYPNMLVSGESLPL-DVKVCVKEQEVLIEMRCPYREYILLDIMD 600

: : . : : : :*:.::

AtTT8 VLHELGIETTAVHTSVNDHDFEAEIRAKVR----GKKASIAEVKRAIHQVIIHDTNL 518

PhAN1 MLRELKVEVVTIQSSLNNGSFFAELRAKVKENIYGRKASILEVKKSIHQLIPRV--- 668

PsA MLRELRIEVIGVQSSLNNGVFVAELRAKVKENGNGKKVSIVEVKRALNQIIPHNNI- 652

VvMYC1 MLRELRLETTTVQSSLTNGVFVAELRAKVKENASGKKASIMEVKRAINQIIPQC--- 701

MdbHLH3 TLRELRIETTVVQSSLNNGFFVAELRAKVKDNVSGKKVSITEVKRVINQIIPQSDS- 709

FabHLH3 TLRDLRIETTVVQSSLNSGTFVAELRAKV-----GKKTTITEVKRAVNQVIPQSDS- 697

RiAN1 TLRDLRIETTVVQSSLNSGTFVAELRAKV-----GKKTSITEVKRAVNQVIPQSDS- 699

RgAN1-1 TLRDLRIETTVVQSSLNSGTFVAELRAKV-----GKKTSITEVKRAVNQVIPQSDS- 699

RubHLH1 TLRDLRIETTVVQSSLNSGTFVAELRAKV-----GKKTSITEVKRAVNQVIPQSDS- 699

RgAN1-3 TLRDLRIETTVVQSSLNSGTFVAELRAKV-----GKKTSITEVKRAVNQVIPQSDS- 699

RgAN1-2 TLRDLRIETTVVQSSLNSGTFVAELRAKV-----GKKTSITEVKRAVNQVIPQSDS- 700

AtEGL3 VISDLNLDSHSVQSSTGDGLLCLTVNCKHKGT---KIATTGMIQEALQRVAWIC--- 596

AtGL3 VISDLHLDSHSVQSSTGDGLLCLTVNCKHKGS---KIATPGMIKEALQRVAWIC--- 637

PhJAF13 ALTGLQMDCHTVQSSNIDGILSISIESKTNVS---KTVSVGTIREALQRVVWKS--- 628

AmDEL AVRRLSLDSETVQSSNRDGMISITIKAKCKGL---KVASASVIKQALQKVTMKS--- 644

RgDEL VTSNLHLDTHSVESSTTDGILSLTIQSKFKGS---TIASAGTIEQALQRIARNC--- 642

RiDEL-1 VTSNLHLDTHSVESSTTDGILSLTIQSKFKGS---TIASAGTIEQALQRIARNC--- 641

RiDEL-2 VTSNLHLDTHSVESSTTDGILSLTIQSKFKGS---TIASAGTIEQALQRIARNC--- 642

VvMYCA1 AINNLHLDCHSVQSSNHDGFLTLTLKSKFRGR---AVASAGMIKQALWRITSKC--- 654

MdbHLH33 AINNLYLDAHSVQSSILDGVLTLSLKSKFRGA---AISPVGMIKQVLWKIAGKC--- 651

* :: :.:* . : :..* :.. : ::
