# Supplementary material for: RNA-seq, de novo transcriptome assembly and flavonoid gene analysis in 13 wild and cultivated berry fruit species with high content of phenolics
Source: BMC Genomics. 2019 Dec 19;20:995. doi: 10.1186/s12864-019-6183-2 (PMC6924045; doi:10.1186/s12864-019-6183-2)
Supplement: Supplementary file 11 — Additional file 11: Figure S4. Phylogenetic relationship, prediction of WD40 motifs and protein sequence alignment of a subset of WDR homologues. [file 12864_2019_6183_MOESM11_ESM.docx]

**Additional file 11: Fig. S4.** Phylogenetic relationship, prediction of WD40 motifs and protein sequence alignment of a subset of WDR homologues.

**1. Phylogenetic tree of a subset of WDR homologues**

**Gene name / bootstrap**

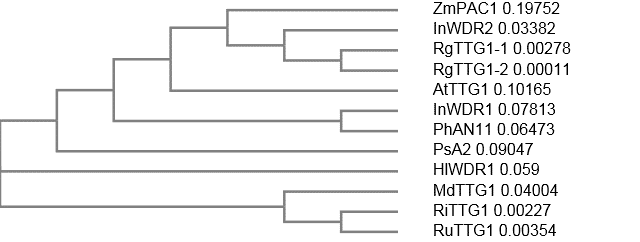

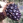

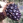

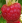


The phylogenetic tree was generated using CLUSTAL OMEGA multiple sequence alignment of protein sequences. The three *Rubus* WDR homologues isolated in this study are indicated by a fruit icon. The accession number or reference of each WDR protein is the following: *Arabidopsis thaliana* AtTTG1 (CAB45372; Walker *et al.* (1999) Plant Cell 11:1337-1350), *Humulus lupulus* HlWDR1 (FN689721.1; Matouŝek *et al.* (2012) BMC Plant Biol. 12: 27), *Ipomoea nil* InWDR1 (AB232779; Morita *et al.* (2006) Plant Cell Physiol. 47:457-470), *I. nil* InWDR2 (AB232780; Morita *et al.* (2006) Plant Cell Physiol. 47:457-470), *Malus x domestica* MdTTG1 (GU173814.1; Brueggemann *et al.* (2010) Plant Cell Rep. 29:285-294), *Petunia x hybrida* PhAN11 (AAC18914; de Vetten *et al.* (1997) Genes Dev. 11:1422-1434), *Pisum sativum* PsA2 (HQ245313; Hellens *et al.* (2010) PLoS ONE 5(10):e13230), *R. genevieri* RgTTG1-1 (MH460860; this manuscript), *R. genevieri* RgTTG1-2 (MH460861; this manuscript), *R. idaeus* cv. Prestige RiTTG1 (MH460862; this manuscript), *Rubus* sp. var. Lochness RuTTG1 (Garcia-Seco *et al.* (2015) PLoS ONE 10(11):e0142639), *Zea mays* ZmPAC1 (AY115485.1; Carey *et al.* (2004) Plant Cell 16:450-464).

**2. Prediction of WD40 repeats in a subset of plant WDR proteins using the WD40-repeat Protein Structure Predictor**

(WDSP, <http://wu.scbb.pkusz.edu.cn/wdsp/predictor.jsp>; Wu *et al.* (2015) Nucleic Acids Res. 43:D339-344)

**Topology of AtTTG1:** The average score of repeats is: 81.82. The estimated Q3 accuracy is: 93±5%

| **Repeats** | **Score** | **Start** | **End** | **Strand_d** | **Loop_da** | **Strand_a** | **Loop_ab** | **Strand_b** | **Loop_bc** | **Strand_c** | **Loop_cd** | **H_bonds** | **Hotspots_on_the_top_face** |
| --- | --- | --- | --- | --- | --- | --- | --- | --- | --- | --- | --- | --- | --- |
| WD1 | 60.79 | 12 | 65 | SETAVT | YDSPYP | L**Y**AMAF | SSLRSSSGH | RIAVGS | FLEDYNNRI | DILSFD | SDSMTV | NA | **Y25** |
| WD2 | 57.06 | 66 | 117 | KPLPNL | SFEHPYP | PTKLMF | SPPSLRRPSSG | DLLASS | GD | FLRLWE | INEDSSTV | NA | **NA** |
| WD3 | 82.88 | 118 | 165 | EPISVL | NNSKTSEFCAP | LTSFDW | NDVEPK | RLGTCS | **I**DT | TCTIWD | IEKS | NA | **I153** |
| WD4 | 119.82 | 166 | 208 | VVETQL | IA**H**DK**E** | V**H**DIAW | GEAR | VFA**S**VS | A**D**G | SVRIFD | LRDKEH | triad | **E177 H179** |
| WD5 | 65.48 | 209 | 254 | STIIYE | SPQPDTP | L**L**RLAW | NKQDLR | YMATIL | **M**DSN | KVVILD | IRSPT | NA | **L223 M240** |
| WD6 | 127.08 | 255 | 303 | MPVAEL | ER**H**QAS | V**N**AIAW | APQSCK | HIC**S**GG | **DD**T | QALI**W**E | LPTVAGPNGI | tetrad | **N268 D285** |
| WD7 | 55.98 | 304 | 340 | DPMSVY | SAGS**E** | I**N**QLQW | SSSQPD | WIGIAF | AN | KMQLLR |  | NA | **E314 N316** |

### Topology of RuTTG1: The average score of repeats is: 81.02. The estimated Q3 accuracy is: 92±5%

| **Repeats** | **Score** | **Start** | **End** | **Strand_d** | **Loop_da** | **Strand_a** | **Loop_ab** | **Strand_b** | **Loop_bc** | **Strand_c** | **Loop_cd** | **H_bonds** | **Hotspots_on_the_top_face** |
| --- | --- | --- | --- | --- | --- | --- | --- | --- | --- | --- | --- | --- | --- |
| WD1 | 54.30 | 13 | 67 | ENSVTY | ESPYP | L**Y**AMAF | SSPRTRHHHHH | RIAVGS | FIEEFSNRV | DILSFD | PDSLTL | NA | **Y25** |
| WD2 | 52.12 | 68 | 120 | KPNPAL | SFDHPYP | PTKLMF | HPNPNTLHKTN | DILASS | GD | YLRLWE | VKDSSVDRL | NA | **NA** |
| WD3 | 79.24 | 121 | 168 | EPISVL | NNSKTSEFCAP | LTSFDW | NEIEPR | RIGTSS | **I**DT | TCTIWD | IEKG | NA | **I156** |
| WD4 | 120.26 | 169 | 212 | VVETQL | IA**H**DK**E** | V**Y**DIAW | GEAR | VFA**S**VS | A**D**G | SVRIFD | LRDKEHS | triad | **E180 Y182** |
| WD5 | 69.49 | 213 | 257 | TIIYES | PQPDTP | L**L**RLAW | NKQDLR | YMATIL | **M**DSN | KVVILD | IRSPT | NA | **L226 M243** |
| WD6 | 126.61 | 258 | 306 | MPVAEL | ER**H**KGS | V**N**AIAW | APQSYR | HIC**S**AG | **DD**S | QALI**W**E | LPTVAGPNGI | tetrad | **N271 D288** |
| WD7 | 64.19 | 307 | 343 | DPMSMY | SAGA**E** | I**N**QLQW | SAAQPD | WIAIAF | TN | KMQLLK |  | NA | **E317 N319** |

**Topology of MdTTG1:** The average score of repeats is: 81.50. The estimated Q3 accuracy is: 92±5%

| **Repeats** | **Score** | **Start** | **End** | **Strand_d** | **Loop_da** | **Strand_a** | **Loop_ab** | **Strand_b** | **Loop_bc** | **Strand_c** | **Loop_cd** | **H_bonds** | **Hotspots_on_the_top_face** |
| --- | --- | --- | --- | --- | --- | --- | --- | --- | --- | --- | --- | --- | --- |
| WD1 | 54.43 | 13 | 68 | ENSVTY | ESPYP | L**Y**AMAF | ASPQTRTRHQHH | RIAVGS | FIEEYSN | RVDILS | FDPDTLSI | NA | **Y25** |
| WD2 | 53.04 | 69 | 118 | KPNPTL | SFDHPYP | PTKLMF | HPNPNALHKTN | DVLASS | GD | YLRLWE | VGDSTV | NA | **NA** |
| WD3 | 80.90 | 119 | 166 | EPIQVL | NNSKTSEFCAP | LTSFDW | NDIEPR | RIGTSS | **I**DT | TCTIWD | IEKG | NA | **I154** |
| WD4 | 120.11 | 167 | 210 | VVETQL | IA**H**DK**E** | V**Y**DIAW | GEAR | VFA**S**VS | A**D**G | SVRIFD | LRDKEHS | triad | **E178 Y180** |
| WD5 | 69.19 | 211 | 255 | TIIYES | PQPDTP | L**L**RLAW | NKQDLR | YMATIL | **M**DSN | KVVILD | IRSPT | NA | **L224 M241** |
| WD6 | 129.32 | 256 | 302 | MPVAEL | ER**H**RGS | V**N**AIAW | APQSCR | HIC**S**AG | **DD**T | QALI**W**D | LPTVAGPN | tetrad | **N269 D286** |
| WD7 | 51.99 | 303 | 341 | GIDPMS | MYSAGA**E** | I**N**QLQW | SAAQPD | WISIAF | SN | KMQLLK |  | NA | **E315 N317** |

**Topology of RiTTG1:** The average score of repeats is: 80.52. The estimated Q3 accuracy is: 92±5%

| **Repeats** | **Score** | **Start** | **End** | **Strand_d** | **Loop_da** | **Strand_a** | **Loop_ab** | **Strand_b** | **Loop_bc** | **Strand_c** | **Loop_cd** | **H_bonds** | **Hotspots_on_the_top_face** |
| --- | --- | --- | --- | --- | --- | --- | --- | --- | --- | --- | --- | --- | --- |
| WD1 | 54.65 | 13 | 67 | ENSVTY | ESPYP | L**Y**AMAF | SSPRTRHHHHH | RIAVGS | FIEEFSNRV | DILSFD | PDTLTL | NA | **Y25** |
| WD2 | 51.85 | 68 | 120 | KPNPAL | SFDHPYP | PTKLMF | HPNPNTLHKTN | DILASS | GD | YLRLWE | VKDSSVDRL | NA | **NA** |
| WD3 | 79.44 | 121 | 168 | EPISVL | NNSKTSEFCAP | LTSFDW | NEIEPR | RIGTSS | **I**DT | TCTIWD | IEKG | NA | **I156** |
| WD4 | 120.26 | 169 | 212 | VVETQL | IA**H**DK**E** | V**Y**DIAW | GEAR | VFA**S**VS | A**D**G | SVRIFD | LRDKEHS | triad | **E180 Y182** |
| WD5 | 69.16 | 213 | 257 | TIIYES | PQPDTP | L**L**RLAW | NKQDLR | YMATIL | **M**DSN | KVVILD | IRSPT | NA | **L226 M243** |
| WD6 | 126.26 | 258 | 306 | MPVAEL | ER**H**KGS | V**N**AIAW | APQSAR | HIC**S**AG | **DD**S | QALI**W**E | LPTVAGPNGI | tetrad | **N271 D288** |
| WD7 | 64.64 | 307 | 343 | DPMSMY | SAGA**E** | I**N**QLQW | SAAQPD | WIAIAF | TN | KMQLLK |  | NA | **E317 N319** |

**Topology of RgTTG1-1:** The average score of repeats is: 82.55. The estimated Q3 accuracy is: 93±5%

| **Repeats** | **Score** | **Start** | **End** | **Strand_d** | **Loop_da** | **Strand_a** | **Loop_ab** | **Strand_b** | **Loop_bc** | **Strand_c** | **Loop_cd** | **H_bonds** | **Hotspots_on_the_top_face** |
| --- | --- | --- | --- | --- | --- | --- | --- | --- | --- | --- | --- | --- | --- |
| WD1 | 61.42 | 16 | 70 | QKRSEI | YTYEAPW**H** | I**Y**AMNW | SVRRDKKY | RLAIAS | LLEQYPN | RVEIVQ | LDDSNGEI | NA | **H29 Y31** |
| WD2 | 59.62 | 71 | 120 | RSDPNL | SFEHPYPP | T**K**TIFI | PDKECQKP | DLLATS | SD | FLRVWS | ISEDSSSV | NA | **K86** |
| WD3 | 80.11 | 121 | 168 | ELKSVL | NGNKNSEFCGP | LTSFDW | NEAEPK | RVGTSS | **I**DT | TCTIWD | IERE | NA | **I156** |
| WD4 | 118.94 | 169 | 211 | AVDTQL | IA**H**DK**E** | V**Y**DIAW | GGVG | VFA**S**VS | A**D**G | SVRVFD | LRDKEH | triad | **E180 Y182** |
| WD5 | 61.78 | 212 | 257 | STIIYE | SSEPDTP | LVRLGW | NKQDPR | YMATII | **M**DSA | KVVVLD | IRFPT | NA | **M243** |
| WD6 | 129.43 | 258 | 306 | LPVVEL | QR**H**QAS | V**N**AVAW | APHSSC | HIC**T**AG | **DD**S | QALI**W**D | LSSMGQPVEG | tetrad | **N271 D288** |
| WD7 | 65.42 | 307 | 345 | GLDPIL | AYTAGA**E** | I**E**QLQW | SSSQPD | WVAIAF | SS | KLQILR |  | NA | **E319 E321** |

**Topology of RgTTG1-2:** The average score of repeats is: 82.85. The estimated Q3 accuracy is: 93±5%

| **Repeats** | **Score** | **Start** | **End** | **Strand_d** | **Loop_da** | **Strand_a** | **Loop_ab** | **Strand_b** | **Loop_bc** | **Strand_c** | **Loop_cd** | **H_bonds** | **Hotspots_on_the_top_face** |
| --- | --- | --- | --- | --- | --- | --- | --- | --- | --- | --- | --- | --- | --- |
| WD1 | 66.68 | 19 | 70 | SEIYTY | EAPW**H** | I**Y**AMNW | SVRRDKKY | RLAIAS | LLEQYPN | RVEIVQ | LDDSNGEI | NA | **H29 Y31** |
| WD2 | 59.87 | 71 | 121 | RSDPNL | SFEHPYPP | T**K**TIFI | PDKECQKP | DLLATS | SD | FLRVWS | ISEDSSSVE | NA | **K86** |
| WD3 | 76.77 | 122 | 168 | LKSVLN | GNKNSEFCGP | LTSFDW | NEAEPK | RIGTSS | **I**DT | TCTIWD | IERE | NA | **I156** |
| WD4 | 118.94 | 169 | 212 | AVDTQL | IA**H**DK**E** | V**Y**DIAW | GGVG | VFA**S**VS | A**D**G | SVRVFD | LRDKEHS | triad | **E180 Y182** |
| WD5 | 64.44 | 213 | 257 | TIIYES | SEPDTP | LVRLGW | NKQDPR | YMATII | **M**DSA | KVVVLD | IRFPT | NA | **M243** |
| WD6 | 129.26 | 258 | 306 | LPVVEL | QR**H**QAS | V**N**AVAW | APHSSC | HIC**T**AG | **DD**S | QALI**W**D | LSSMGQPVEG | tetrad | **N271 D288** |
| WD7 | 65.62 | 307 | 345 | GLDPIL | AYTAGA**E** | I**E**QLQW | SSSQPD | WVAIAF | SS | KLQILR |  | NA | **E319 E321** |

**3. Protein sequence alignment of a subset of plant WDR homologues in comparison to the *Rubus* TTG1 homologues isolated in this study**

Four highly conserved WD-repeat domains found in plant WDR proteins related to anthocyanin biosynthesis are highlighted in light blue (based on Brueggemann *et al.* (2010) Plant Cell Rep. 29:285-294).

CLUSTAL O(1.2.4) multiple sequence alignment

ZmPAC1 MDPPKPPSSVASSSGPETPNPHAFTCELPHSIYALAFSPVAP--------VLASGSFLED 52

InWDR2 MGASSDPNQ--DGSDEQQRRSEIYTYEAPWHIYAMNWSVRKD-----KRYRLAIASLLEQ 53

RgTTG1-1 MGASSDPNQ--DGSDEQQKRSEIYTYEAPWHIYAMNWSVRRD-----KKYRLAIASLLEQ 53

RgTTG1-2 MGASSDPNQ--DGSDEQQKRSEIYTYEAPWHIYAMNWSVRRD-----KKYRLAIASLLEQ 53

AtTTG1 MDNS-------AP-DSLSRSETAVTYDSPYPLYAMAFSSLRSS----SGHRIAVGSFLED 48

InWDR1 MENS--------TQGSNLRSENSVTYESPYPIFAMAVSSFAAAHHGLRRRSVAVGSFLEE 52

PhAN11 MENS-------SQESQHLRSENSVTYDSTYPIYSMAFSSFPT-----PRRRIAVGSFIEE 48

PsA2 MDNS-------TQESHLRSDNNSVTYDSPHPLYAMAFSSNPNP---QHHQRIAVGSFIEE 50

HlWDR1 MENS-------TQESHLRPD-NSVTYDSPYPMYAMALSSTQTR---NRHHRIAVGSLIEE 49

MdTTG1 MENS-------TQESHLRAE-NSVTYESPYPLYAMAFASPQTRT-RHQHHRIAVGSFIEE 51

RiTTG1 MDNS-------TQESHLGPE-NSVTYESPYPLYAMAFSSPRTRH--HHHHRIAVGSFIEE 50

RuTTG1 MDNS-------TQESHLGPE-NSVTYESPYPLYAMAFSSPRTRH--HHHHRIAVGSFIEE 50

* . * : :::: : :* .*::*:

ZmPAC1 LHNRVSLLSFDPVRPSAASFRALPALSFDHPYPPTKLQFNPRAA------APSLLASSAD 106

InWDR2 YPNRVEIVQLDDS---NGEIRSDPNLSFEHPYPPTKVIFIPDKEC----QKPDLIATSSD 106

RgTTG1-1 YPNRVEIVQLDDS---NGEIRSDPNLSFEHPYPPTKTIFIPDKEC----QKPDLLATSSD 106

RgTTG1-2 YPNRVEIVQLDDS---NGEIRSDPNLSFEHPYPPTKTIFIPDKEC----QKPDLLATSSD 106

AtTTG1 YNNRIDILSFDSD---SMTVKPLPNLSFEHPYPPTKLMFSPPSLR--RPSSGDLLASSGD 103

InWDR1 YKNRVEILSFEED---TVTLKTNPGLAFDHPYPPTKLMFHPNPTAS--MKSADLLVSSGD 107

PhAN11 LNNRVELLSFNEE---TLTLNPIPNLSFDHPYPPTKLMFHPNPIKS----NNDILASSGD 101

PsA2 YTNRVDILSFNPD---TLSIKPQPSLSFDHPYPPTKLMFHPATHSSLQKTSSDLLATSGD 107

HlWDR1 FSNRVDLLSFDPE---TLTLNSQPSLSFDHPYPPTKLMFHPS--T--LQKSSDVLASSGD 102

MdTTG1 YSNRVDILSFDPD---TLSIKPNPTLSFDHPYPPTKLMFHPNPNA--LHKTNDVLASSGD 106

RiTTG1 FSNRVDILSFDPD---TLTLKPNPALSFDHPYPPTKLMFHPNPNT--LHKTNDILASSGD 105

RuTTG1 FSNRVDILSFDPD---SLTLKPNPALSFDHPYPPTKLMFHPNPNT--LHKTNDILASSGD 105

**:.::.:: .. * *:*:******* * * .::.:*.*

ZmPAC1 TLRIWHTPLDDLSDTAPAPELRSVLDNRKASSEFCAPLTSFDWNEVEPRRIGTASIDTTC 166

InWDR2 YLRVWRVADDNS-RVEI----KTLL-NNNRNSEFSGPLTSFDWNEAEPKRIGTSSIDTTC 160

RgTTG1-1 FLRLWSISEDSS-SVEL----KSVL-NGNKNSEFCGPLTSFDWNEAEPKRVGTSSIDTTC 160

RgTTG1-2 FLRLWSISEDSS-SVEL----KSVL-NGNKNSEFCGPLTSFDWNEAEPKRIGTSSIDTTC 160

AtTTG1 FLRLWEINEDSS-TVEP----ISVL-NNSKTSEFCAPLTSFDWNDVEPKRLGTCSIDTTC 157

InWDR1 YLRLWEVREASI---EP----VSTL-NNSKTSEYCAPLTSFDWNEVEPRRIGTSSIDTTC 159

PhAN11 YLRLWEVKESSI---EP----LFTL-NNSKTSEYCAPLTSFDWNEVEPKRIGTSSIDTTC 153

PsA2 YLRLWEVRENSV---EA----LSLF-NNSKTSEFCAPLTSFDWNEIEPKRIGTSSIDTTC 159

HlWDR1 YLRLWEVRDNSI---EP----ISVL-NNSKTSEFCAPLTSFDWNEIEPRRIGTSSIDTTC 154

MdTTG1 YLRLWEVGDSTV---EP----IQVL-NNSKTSEFCAPLTSFDWNDIEPRRIGTSSIDTTC 158

RiTTG1 YLRLWEVKDSSVDRLEP----ISVL-NNSKTSEFCAPLTSFDWNEIEPRRIGTSSIDTTC 160

RuTTG1 YLRLWEVKDSSVDRLEP----ISVL-NNSKTSEFCAPLTSFDWNEIEPRRIGTSSIDTTC 160

**:* : * . .**:..********: **:*:**.******

ZmPAC1 TVWDIDRGVVETQLIAHDKAVHDIAWGEAGVFASVSADGSVRVFDLRDKEHSTIVYESPR 226

InWDR2 TIWDIERETVDTQLIAHDKEVYDIAWGGVGVFASVSADGSVRVFDLRDKEHSTIIYESSE 220

RgTTG1-1 TIWDIEREAVDTQLIAHDKEVYDIAWGGVGVFASVSADGSVRVFDLRDKEHSTIIYESSE 220

RgTTG1-2 TIWDIEREAVDTQLIAHDKEVYDIAWGGVGVFASVSADGSVRVFDLRDKEHSTIIYESSE 220

AtTTG1 TIWDIEKSVVETQLIAHDKEVHDIAWGEARVFASVSADGSVRIFDLRDKEHSTIIYESPQ 217

InWDR1 TIWDIEKGVVETQLIAHDKEVYDIAWGEAGVFSSVSADGSVRIFDLRDKEHSTIIYESPQ 219

PhAN11 TIWDVEKGVVETQLIAHDKEVYDIAWGEAGVFASVSADGSVRIFDLRDKEHSTIIYESPT 213

PsA2 TIWDIERGVVETQLIAHDKEVYDIAWGESRVFASVSADGSVRIFDLRDKEHSTIIYESPQ 219

HlWDR1 TIWDIDKGVVETQLIAHDKEVYDIAWGEARVFASVSADGSVRIFDLRDKEHSTIIYESPQ 214

MdTTG1 TIWDIEKGVVETQLIAHDKEVYDIAWGEARVFASVSADGSVRIFDLRDKEHSTIIYESPQ 218

RiTTG1 TIWDIEKGVVETQLIAHDKEVYDIAWGEARVFASVSADGSVRIFDLRDKEHSTIIYESPQ 220

RuTTG1 TIWDIEKGVVETQLIAHDKEVYDIAWGEARVFASVSADGSVRIFDLRDKEHSTIIYESPQ 220

*:**::: .*:******** *:***** **:*********:***********:***

ZmPAC1 PDTPLLRLAWNRSDLRYMAALLMDSSAVVVLDIRAPGVPVAELHRHRACANAVAWAPQAT 286

InWDR2 PDTPLVRLGWNKQDPRYMATIIMDSSKVVVLDIRFPTLPVVELQRHQASVNAVAWAPHSS 280

RgTTG1-1 PDTPLVRLGWNKQDPRYMATIIMDSAKVVVLDIRFPTLPVVELQRHQASVNAVAWAPHSS 280

RgTTG1-2 PDTPLVRLGWNKQDPRYMATIIMDSAKVVVLDIRFPTLPVVELQRHQASVNAVAWAPHSS 280

AtTTG1 PDTPLLRLAWNKQDLRYMATILMDSNKVVILDIRSPTMPVAELERHQASVNAIAWAPQSC 277

InWDR1 PDTPLLRLAWNKQDLRYMATILMDSNKVVILDIRSPAMPVAELERHNASVNAIAWAPQSS 279

PhAN11 PDTPLLRLAWNKQDLRYMATILMDSNKVVILDIRSPAMPVAELERHQASVNAIAWAPQSC 273

PsA2 PDTPLLRLAWNKKDLRYMATILMDSNKVVILDIRSPTTPVAELERHRAGVNAIAWAPRSS 279

HlWDR1 PDTPLLRLAWNKQDLRYMATILMDSNKVVILDIRSPSIPVAELERHRAGVNAIAWAPQSY 274

MdTTG1 PDTPLLRLAWNKQDLRYMATILMDSNKVVILDIRSPTMPVAELERHRGSVNAIAWAPQSC 278

RiTTG1 PDTPLLRLAWNKQDLRYMATILMDSNKVVILDIRSPTMPVAELERHKGSVNAIAWAPQSA 280

RuTTG1 PDTPLLRLAWNKQDLRYMATILMDSNKVVILDIRSPTMPVAELERHKGSVNAIAWAPQSY 280

*****:**.**:.* ****:::*** **:**** * **.**.**.. .**:****::

ZmPAC1 RHLCSAGDDGQALIWELPETAAAVPAEGIDPVLVYDAGAEINQLQWAAAHPDWMAIAFEN 346

InWDR2 CHICTAGDDSQALIWDLSSMGQPI-EGGLDPILAYTAGAEIEQLQWSSSQPDWVAIAFSN 339

RgTTG1-1 CHICTAGDDSQALIWDLSSMGQPV-EGGLDPILAYTAGAEIEQLQWSSSQPDWVAIAFSS 339

RgTTG1-2 CHICTAGDDSQALIWDLSSMGQPV-EGGLDPILAYTAGAEIEQLQWSSSQPDWVAIAFSS 339

AtTTG1 KHICSGGDDTQALIWELPTVAGP---NGIDPMSVYSAGSEINQLQWSSSQPDWIGIAFAN 334

InWDR1 RHISSAGDDGQALIWELPTVAGP---NGIDPMSMYSAGAEINQLQWSAAQPDWIAIAFSN 336

PhAN11 RHICSGGDDGQALIWELPTVAGP---NGIDPMSMYSAGAEINQLQWSPAQRDWIAIAFSN 330

PsA2 KHICSAGDDTQALMWELPTVAGP---NGIDPMSMYSSGYEINQLQWSAAQPDWIAIAFAN 336

HlWDR1 RHICSAGDDSQALIWELPTVAGP---NGIDPISMFSAGSEINQLQWSAAQPDWIAIAFSN 331

MdTTG1 RHICSAGDDTQALIWDLPTVAGP---NGIDPMSMYSAGAEINQLQWSAAQPDWISIAFSN 335

RiTTG1 RHICSAGDDSQALIWELPTVAGP---NGIDPMSMYSAGAEINQLQWSAAQPDWIAIAFTN 337

RuTTG1 RHICSAGDDSQALIWELPTVAGP---NGIDPMSMYSAGAEINQLQWSAAQPDWIAIAFTN 337

*:.:.*** ***:*:* . *:**: : :* **:****: :: **:.*** .

ZmPAC1 KVQLLRV 353

InWDR2 KLQILRV 346

RgTTG1-1 KLQILRV 346

RgTTG1-2 KLQILRV 346

AtTTG1 KMQLLRV 341

InWDR1 KLQMLKV 343

PhAN11 KLQLLKV 337

PsA2 KMQLLRV 343

HlWDR1 KMQLLKV 338

MdTTG1 KMQLLKV 342

RiTTG1 KMQLLKV 344

RuTTG1 KMQLLKV 344

*:*:*:*
