# Supplementary material for: RNA-seq, de novo transcriptome assembly and flavonoid gene analysis in 13 wild and cultivated berry fruit species with high content of phenolics
Source: BMC Genomics. 2019 Dec 19;20:995. doi: 10.1186/s12864-019-6183-2 (PMC6924045; doi:10.1186/s12864-019-6183-2)
Supplement: Supplementary file 13 — Additional file 13: Figure S5. Production of anthocyanins in leaves of two accessions of N. benthamiana, JIC-LAB strain and cv. NT, following transient overexpression of regulatory genes from R. genevieri and R. idaeus cv. Prestige at various time points after infiltration (4 dpi to 14 dpi) alone or in combination. [file 12864_2019_6183_MOESM13_ESM.pdf]

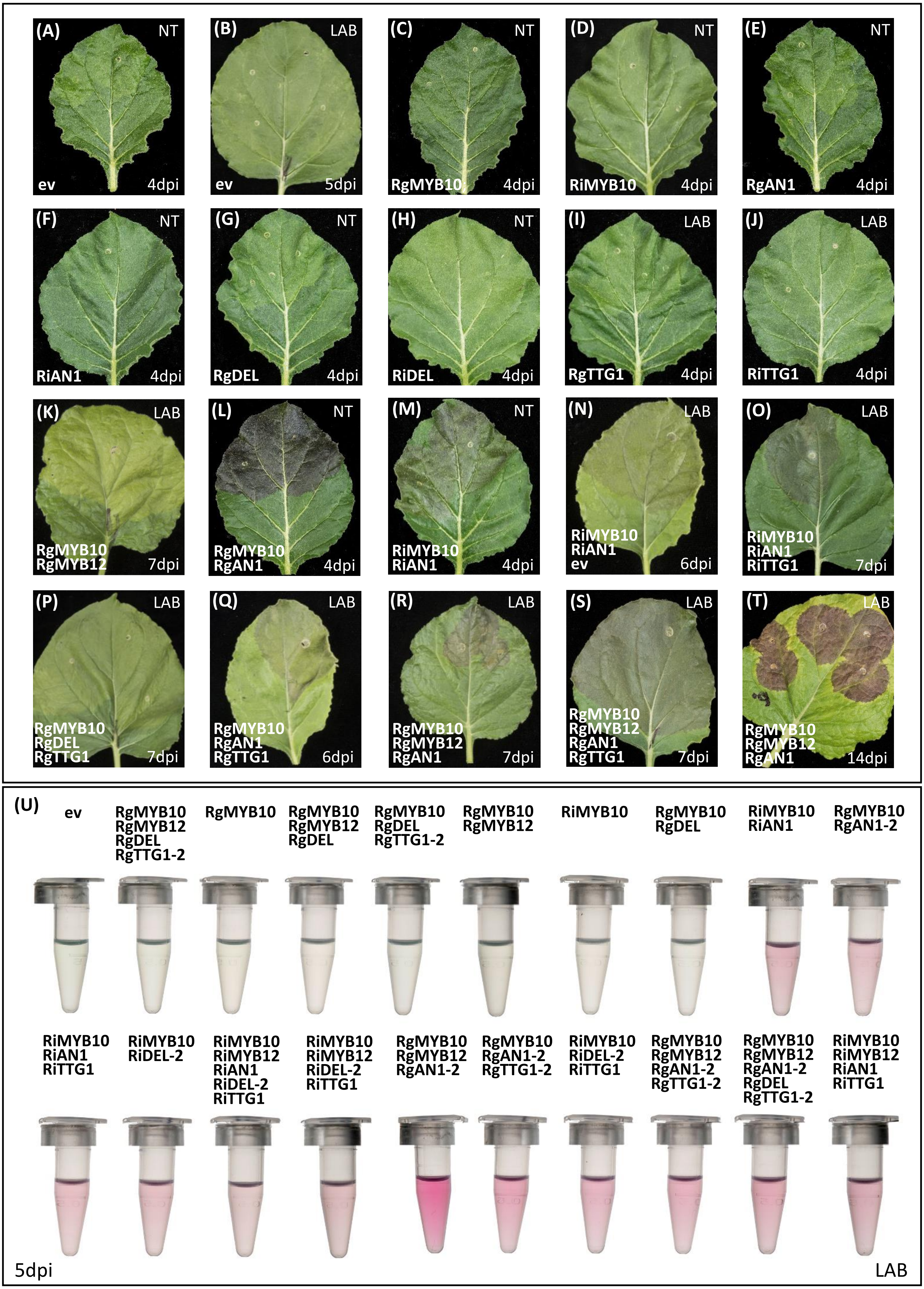
(A) NT ev 4dpi
(B) LAB ev 5dpi
(C) NT RgMYB10 4dpi
(D) NT RiMYB10 4dpi
(E) NT RgAN1 4dpi
(F) NT RiAN1 4dpi
(G) NT RgDEL 4dpi
(H) NT RiDEL 4dpi
(I) LAB RgTTG1 4dpi
(J) LAB RiTTG1 4dpi
(K) LAB RgMYB10 RgMYB12 7dpi
(L) NT RgMYB10 RgAN1 4dpi
(M) NT RiMYB10 RiAN1 4dpi
(N) LAB RiMYB10 RiAN1 ev 6dpi
(O) LAB RiMYB10 RiAN1 RiTTG1 7dpi
(P) LAB RgMYB10 RgDEL RgTTG1 7dpi
(Q) LAB RgMYB10 RgAN1 RgTTG1 6dpi
(R) LAB RgMYB10 RgMYB12 RgAN1 7dpi
(S) LAB RgMYB10 RgMYB12 RgAN1 RgTTG1 7dpi
(T) LAB RgMYB10 RgMYB12 RgAN1 14dpi
(U)
ev
RgMYB10 RgMYB12 RgDEL RgTTG1-2
RgMYB10
RgMYB10 RgMYB12 RgDEL
RgMYB10 RgDEL RgTTG1-2
RgMYB10 RgMYB12
RiMYB10
RgMYB10 RgDEL
RiMYB10 RiAN1
RgMYB10 RgAN1-2
RiMYB10 RiAN1 RiTTG1
RiMYB10 RiDEL-2
RiMYB10 RiMYB12 RiAN1 RiDEL-2 RiTTG1
RiMYB10 RiMYB12 RiDEL-2 RiTTG1
RgMYB10 RgMYB12 RgAN1-2
RgMYB10 RgAN1-2 RgTTG1-2
RiMYB10 RiDEL-2 RiTTG1
RgMYB10 RgMYB12 RgAN1-2 RgTTG1-2
RgMYB10 RgMYB12 RgAN1-2 RgDEL RgTTG1-2
RiMYB10 RiMYB12 RiAN1 RiTTG1
5dpi
LAB

Additional file 13: Fig. S5. Production of anthocyanins in leaves of two accessions of *N. benthamiana,* strain JIC-LAB and cv. NT, following transient overexpression of regulatory genes from *R. genevieri* and *R. idaeus* cv. Prestige at various time points after infiltration (4 dpi to 14 dpi) alone or in combination. Results are shown from independent experiments.

(A-K) *Rubus* MYB10, MYB12 and bHLH homologues as well as WDR alone did not induce red pigmentation observable by the naked eye in agroinfiltrated leaf patches. **(A)** Empty vector construct (ev) infiltrated *N. benthamiana* cv. NT (NT) leaf at 4 dpi; **(B)** ev-infiltrated *N. benthamiana* strain JIC-LAB (LAB) leaf at 5 dpi; **(C)** *RgMyb10* infiltrated *N. benthamiana* cv. NT leaf at 4 dpi; **(D)** *RiMyb10* infiltrated *N. benthamiana* cv. NT leaf at 4 dpi; **(E)** *RgAn1-2* infiltrated *N. benthamiana* cv. NT leaf at 4 dpi; **(F)** *RiAn1* infiltrated *N. benthamiana* cv. NT leaf at 4 dpi; **(G)** *RgDel* infiltrated *N. benthamiana* cv. NT leaf at 4 dpi; **(H)** *RiDel-2* infiltrated *N. benthamiana* cv. NT leaf at 4 dpi; **(I)** *RgTTG1-2* infiltrated *N. benthamiana* cv. NT leaf at 4 dpi; **(J)** *RiTTG1* infiltrated *N. benthamiana* cv. NT leaf at 4 dpi; **(K)** *RgMyb10* and *RgMyb12* co-infiltrated *N. benthamiana* strain JIC-LAB leaf at 7 dpi;

(L-T) Addition of bHLH and/or WDR regulatory proteins to MYB10 TFs increases anthocyanin production in infiltrated *N. benthamiana* leaves. **(L)** *RgMyb10* and *RgAn1-2* co-infiltrated *N. benthamiana* cv. NT leaf at 4 dpi; **(M)** *RiMyb10* and *RiAn1* co-infiltrated *N. benthamiana* cv. NT leaf at 4 dpi; **(N)** *RiMyb10, RiAn1* and ev co-infiltrated *N. benthamiana* strain JIC-LAB leaf at 6 dpi; **(O)** *RiMyb10, RiAn1* and *RiTTG1* co-infiltrated *N. benthamiana* strain JIC-LAB leaf at 7 dpi; **(P)** *RgMyb10, RgDel* and *RgTTG1-2* co-infiltrated *N. benthamiana* strain JIC-LAB leaf at 7 dpi; **(Q)** *RgMyb10*, *RgAn1-2* and *RgTTG1-2* co-infiltrated *N. benthamiana* strain JIC-LAB leaf at 6 dpi; **(R, T)** *RgMyb10*, *RgMyb12* and *RgAn1-2* co-infiltrated *N. benthamiana* strain JIC-LAB leaf at 7 dpi **(R)** and at 14 dpi **(T)**; **(S)** *RgMyb10*, *RgMyb12, RgAn1-2* and *RgTTG1-2* co-infiltrated *N. benthamiana* strain JIC-LAB leaf at 7 dpi;

(U) Methanol extracts from *N. benthamiana* strain JIC-LAB leaves transiently expressing *Rubus* flavonoid regulatory genes (*Myb*, *bHLH* and *WDR*) at 5 dpi. Extracts derived from a 1.8-cm diameter leaf disc per mix that was incubated in 2 ml methanol: water: HCl (80:20:1, v/v/v) overnight.
