# Supplementary material for: RNA-seq, de novo transcriptome assembly and flavonoid gene analysis in 13 wild and cultivated berry fruit species with high content of phenolics
Source: BMC Genomics. 2019 Dec 19;20:995. doi: 10.1186/s12864-019-6183-2 (PMC6924045; doi:10.1186/s12864-019-6183-2)
Supplement: Supplementary file 14 — Additional file 14: Figure S6. Examples of anthocyanin formation in kanamycin, hygromycin and/or PPT-resistant N. benthamiana calli and shoots transformed with Rubus Myb, bHLH and WDR regulatory genes. [file 12864_2019_6183_MOESM14_ESM.pdf]

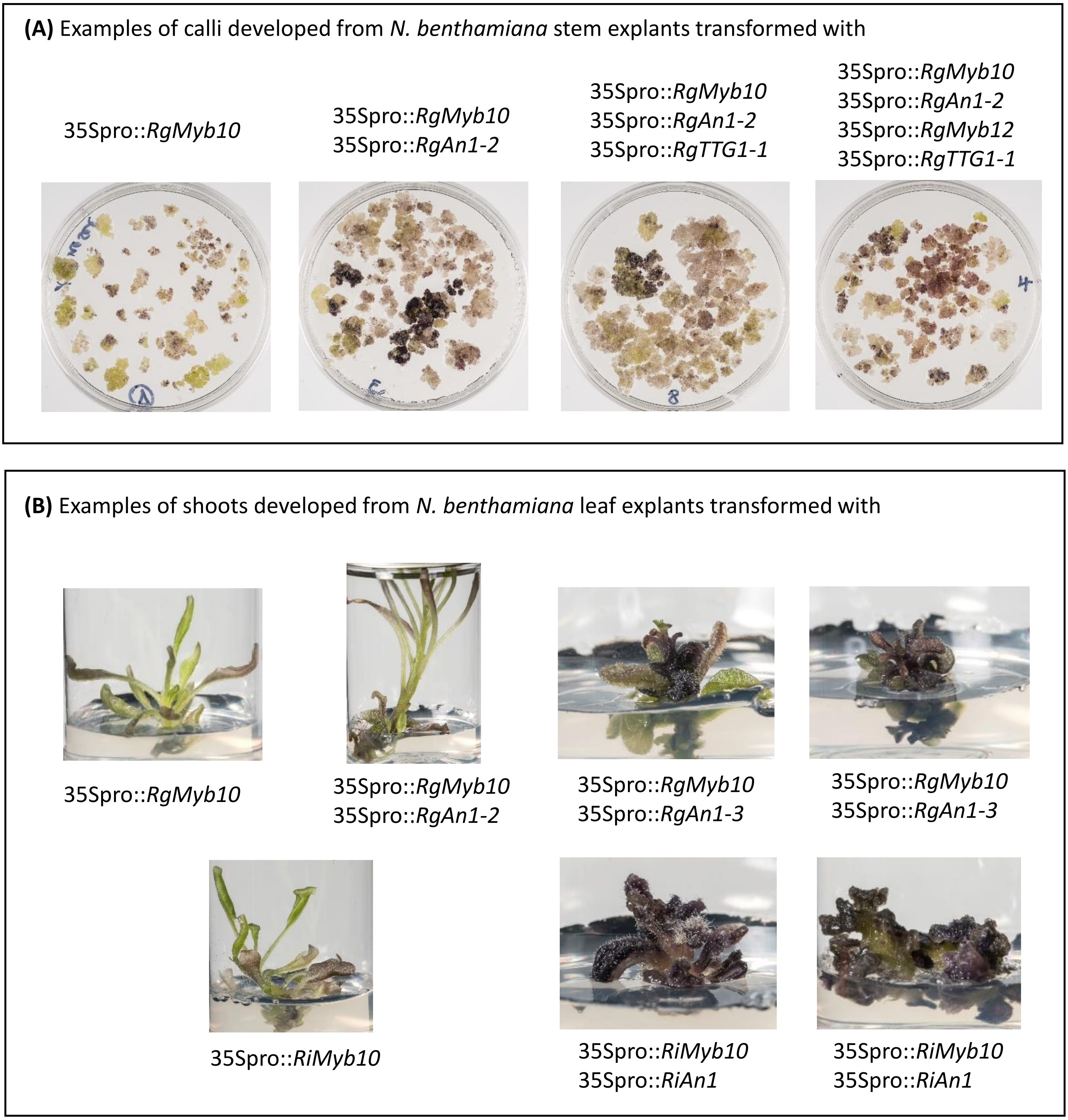

Additional file 14: Fig. S6. Examples of anthocyanin formation in kanamycin, hygromycin and/or PPT-resistant *N. benthamiana* (accession JIC-LAB) calli **(A)** and shoots **(B)** transformed with *Rubus Myb*, *bHLH* and *WDR* regulatory genes.
