# Supplementary material for: RNA-seq, de novo transcriptome assembly and flavonoid gene analysis in 13 wild and cultivated berry fruit species with high content of phenolics
Source: BMC Genomics. 2019 Dec 19;20:995. doi: 10.1186/s12864-019-6183-2 (PMC6924045; doi:10.1186/s12864-019-6183-2)
Supplement: Supplementary file 16 — Additional file 16: Step-by-step guide of the phylogenetic analysis of the transcriptomes of the 13 berry fruit species together with the reference genomes of A. thaliana, P. trichocarpa, G. max, V. vinifera, S. lycopersicum, O. sativa and A. trichopoda. [file 12864_2019_6183_MOESM16_ESM.docx]

**Additional file 16:** Step-by-step guide of the phylogenetic analysis of the transcriptomes of the 13 berry fruit species together with the reference genomes of *A. thaliana*, *P. trichocarpa*, *G. max*, *V. vinifera*, *S. lycopersicum*, *O. sativa* and *A. trichopoda*.

1. The transcriptomes of each species were translated using the script tr2aacds.pl

Bash

#/bin/bash!

ncpu=4

maxmem=12000 # in Megabytes

evigene=$HOME/Documents/software/evigene19jan01

## Required software

#t2ac: app=cd-hit-est, path= echo MISSING_cd-hit-est

#export PATH=$HOME/bio/cdhit/bin:$PATH

##t2ac: app=fastanrdb, path= echo MISSING_fastanrdb

#export fastanrdb=$HOME/bio/exonerate/bin/fastanrdb

##t2ac: app=blastn, path= echo MISSING_blastn

#export PATH=$HOME/bio/ncbi2227/bin:$PATH

berries_path=Berries

ev_out="01.evigene"

#mkdir -p 01.evigene

for fastafile in $berries_path/Transcriptomes/*.fasta.gz; do

filename=$(basename -- "$fastafile")

extension="${filename##*.}"

filename="${filename%.*.*}"

echo $filename

#mkdir $ev_out/$filename

tr_folder=$ev_out/$filename

tr_path=$tr_folder/tr_assembly.fasta

#gunzip -c $fastafile > $tr_path

cd $tr_folder

$evigene/scripts/prot/tr2aacds.pl -NCPU $ncpu -MAXMEM $maxmem -log -cdna $fastafile

done

EvidentialGenes(http://arthropods.eugenes.org/EvidentialGene/trassembly.html, cdhit, blast, ensembl)

Bash

#/bin/bash!

berries_path=Berries

ev_out="01.evigene"

mkdir -p 02.filter_aa

for fastafile in $berries_path/$ev_out/okayset/*.okay.aa; do

filename=$(basename -- "$fastafile")

extension="${filename##*.}"

filename="${filename%.*.*}"

echo $filename

bioawk -c fastx '{ if(length($seq) > 100) { print ">"$name; print $seq }}' $fastafile | gzip -c > 02.filter_aa/${filename}_min100aa.fa.gz

done

2. The short proteins were filtered with the following script:

Bash

#/bin/bash!

berries_path=Berries

ev_out="01.evigene"

mkdir -p 02.filter_aa

for fastafile in $berries_path/$ev_out/okayset/*.okay.aa; do

filename=$(basename -- "$fastafile")

extension="${filename##*.}"

filename="${filename%.*.*}"

echo $filename

bioawk -c fastx '{ if(length($seq) > 100) { print ">"$name; print $seq }}' $fastafile | gzip -c > 02.filter_aa/${filename}_min100aa.fa.gz

done

3. The proteins were renamed to have the species as a prefix as follows:

Bash

#!/bin/bash

out_folder="/Users/ramirezr/Documents/JIC/berries/03.adjusted/"

gunzip -c 02.filter_aa/Aristotelia_chilensis_min100aa.fa.gz | bioawk -c fastx '{ print ">ach|"$name; print $seq }' > $out_folder/ach.fasta

gunzip -c 02.filter_aa/Corema_album_min100aa.fa.gz | bioawk -c fastx '{ print ">cal|"$name; print $seq }' > $out_folder/cal.fasta

gunzip -c 02.filter_aa/Ribes_nigrum_cv_BenHope_min100aa.fa.gz | bioawk -c fastx '{ print ">rnib|"$name; print $seq }' > $out_folder/rnib.fasta

gunzip -c 02.filter_aa/Rubus_genevieri_min100aa.fa.gz | bioawk -c fastx '{ print ">rge|"$name; print $seq }' > $out_folder/rge.fasta

gunzip -c 02.filter_aa/Rubus_idaeus_cv_Prestige_min100aa.fa.gz | bioawk -c fastx '{ print ">ridp|"$name; print $seq }' > $out_folder/ridp.fasta

gunzip -c 02.filter_aa/Ugni_molinae_min100aa.fa.gz | bioawk -c fastx '{ print ">umo|"$name; print $seq }' > $out_folder/umo.fasta

gunzip -c 02.filter_aa/Vaccinium_uliginosum_min100aa.fa.gz | bioawk -c fastx '{ print ">vul|"$name; print $seq }' > $out_folder/vul.fasta

gunzip -c 02.filter_aa/Berberis_buxifolia_min100aa.fa.gz | bioawk -c fastx '{ print ">bbu|"$name; print $seq }' > $out_folder/bbu.fasta

gunzip -c 02.filter_aa/Lonicerea_caerula_S322-3_min100aa.fa.gz | bioawk -c fastx '{ print ">lca|"$name; print $seq }' > $out_folder/lca.fasta

gunzip -c 02.filter_aa/Ribes_nigrum_var_sibiricum_cv_Biryusinka_min100aa.fa.gz | bioawk -c fastx '{ print ">rnis|"$name; print $seq }' > $out_folder/rnis.fasta

gunzip -c 02.filter_aa/Rubus_idaeus_cv_Octavia_min100aa.fa.gz | bioawk -c fastx '{ print ">rido|"$name; print $seq }' > $out_folder/rido.fasta

gunzip -c 02.filter_aa/Rubus_vagabundus_min100aa.fa.gz | bioawk -c fastx '{ print ">rva|"$name; print $seq }' > $out_folder/rva.fasta

gunzip -c 02.filter_aa/Vaccinium_corymbosum_min100aa.fa.gz | bioawk -c fastx '{ print ">vac|"$name; print $seq }' > $out_folder/vac.fasta

4. A single database of proteins was created to blast all the proteins against each other:

Bash

#!/bin/bash

#SBATCH --mem=8Gb

#SBATCH -p RG-Cristobal-Uauy,jic-medium,nbi-medium

#SBATCH -J blast_berries

#SBATCH -N 8

#SBATCH -n 1

#SBATCH --cores-per-socket=8

#SBATCH -o log/blast_%A_%a.out

#SBATCH --time=4-00:00:00

#SBATCH --array=15,17

## SBATCH --array=0-18

## SBATCH --array=2,4

## SBATCH --array=0-12

source blast+-2.9.0

i=$SLURM_ARRAY_TASK_ID

input_folder=03.adjusted/blast

output_folder=04.blast/

db="03.adjusted/my_prot_blast_db"

berries=(ach bbu cal lca rge rido ridp rnib rnis rva umo vac vul vvi sly ptr osa gma ath)

#i=12

b=${berries[i]}

echo $b

input=$input_folder/$b.fasta.gz

output=$output_folder/$b.tab.gz

echo $input

echo $output

gunzip -c $input | blastp -evalue 1e-5 -num_threads 16 -db $db -outfmt 6 -out - -query - | gzip -c > $output

5. All the results from the blast searches were concatenated:

gunzip -c ach.tab.gz ath.tab.gz bbu.tab.gz cal.tab.gz gma.tab.gz lca.tab.gz osa.tab.gz ptr.tab.gz rge.tab.gz rido.tab.gz ridp.tab.gz rnib.tab.gz rnis.tab.gz rva.tab.gz sly.tab.gz umo.tab.gz vac.tab.gz vul.tab.gz vvi.tab.gz atr.tab.gz > all_berries_blast.tab

6. The similarities were calculated:

Bash

perl orthomclBlastParser all_berries_blast.tab blast > similarSequences.txt

7. The data was loaded to the MySQL database:

Bash

perl orthomclInstallSchema ../local_mysql_config.config

mysql -u XXXX -p --local-infile orthomcl

SQL

SET GLOBAL local_infile = 1;

LOAD DATA

LOCAL INFILE "similarSequences.txt"

REPLACE INTO TABLE SimilarSequencesorthomcl

FIELDS TERMINATED BY '\t';

8. The two following scripts from OrthoMCL were run to find the pairs and to export them to OrthoMCL:

Note: Before running OrthoMCL, the buffer size needs to be increased as follows:

SQL

SET GLOBAL innodb_buffer_pool_size=2147483648;

Then OrthoMCL can be run:

Bash

perl orthomclPairs local_mysql_config.config ./log/orthomcl_pairs.log cleanup=yes

perl orthomclDumpPairsFiles ../orthomclSoftware-v2.0.9/local_mysql_config.config

9. Running MCL:

Bash

mcl mclInput --abc -I 1.5 -o mclOutput -te 4

10. Extracting the groups:

Bash

perl orthomclMclToGroups berries_cluster_ 100000 < /07.dump_pairs/mclOutput > groups.txt

**Preparing the trees**

The amino acid sequences were prepared to produce the multiple sequence alignment of each group. The steps were:

1. Read all the groups and only keep the groups that have 20 sequences and all the sequences that belong to a different organism.
2. Read the protein sequences of all the genes that were in the selected groups.
3. Run Muscle v3.8.1551 on the sequence of each group. All the alignments were saved independently in case of further analysis in a particular group.
4. Get the longest block of continuously aligned sequences. This is because regions with insertions/deletions across different samples tend to diverge more, adding noise to the tree creation. This also removes the beginning and the end of genes that may not be completely assembled. The maximum region with gaps is 5 amino acids (aa).
5. Only the groups with the longest block over 20 aa were kept and a "super matrix" of alignments was done by concatenating the sequences. This kept 205 groups.

**ProtTest**

ProTest was used to find the "best" models, use as follow:

Bash

java -jar prottest-3.4.2.jar -i ../07.\ muscle_alignments/concat_proteins.phylip -all-distributions -F -AIC -BIC -tc 0.5 -threads 4

The best models were:

Best model according to BIC: JTT+I+G+F

Best model according to AIC: JTT+I+G+F

**RaXML**

The command to use is:

Bash

raxmlHPC-PTHREADS-AVX -m PROTGAMMAJTT -o atr,osa -T 4 -n 03_osa.otr.out.gammajtt -s ../07.muscle_alignments/concat_proteins.phylip -p 1203

**MCMCtree**

To remove the branch lengths from the tree mcmctree.ctl was used:

seed = -1

seqfile = concat_proteins.phy

treefile = RAxML_bestTree.03_osa.times4.tree

mcmcfile = mcmc.txt

print=1

outfile = test.ages

seqtype = 2

usedata = 2 in.BV

clock = 3

cleandata = 1

burnin = 500000

sampfreq = 50

nsample = 100000

BDparas = 2 2 .1

kappa_gamma = 6 2

alpha_gamma = 1 1

rgene_gamma = 100 1000 2 0 * conditional iid prior for locus rates

sigma2_gamma = 10 100 2 * conditional iid prior for sigma^2 (for clock=2 or 3)

finetune = 1: .1 .1 .1 .1 .1 .1 * auto (0 or 1) : times, rates, mixing...
